# Mortality and morbidity after total intravenous anaesthesia versus inhalational anaesthesia: a systematic review and meta-analysis

**DOI:** 10.1016/j.eclinm.2024.102636

**Published:** 2024-05-14

**Authors:** Jasper M. Kampman, Jeroen Hermanides, Markus W. Hollmann, Coenraad N. Gilhuis, Wouter AH. Bloem, Stefan Schraag, Lorenzo Pradelli, Sjoerd Repping, Nicolaas H. Sperna Weiland

**Affiliations:** a Department of Anaesthesiology, Amsterdam UMC, University of Amsterdam, Amsterdam, the Netherlands; b Amsterdam UMC Centre for Sustainable Healthcare, Amsterdam UMC, Amsterdam, the Netherlands; c Equalis Strategy and Modeling, Utrecht, the Netherlands; d Becton–Dickinson, Winnersh, Wokingham, United Kingdom; e AdRes-Health Economics and Outcome Research, Torino, Italy; f Amsterdam UMC, University of Amsterdam, Amsterdam, the Netherlands; g Healthcare Evaluation and Appropriate Use, National Healthcare Institute, Diemen, the Netherlands

**Keywords:** Total intravenous anaesthesia, Inhalational anaesthesia, Volatile anaesthetics, Postoperative outcome, Environmental sustainability

## Abstract

**Background:**

General anaesthesia is provided to more than 300 million surgical patients worldwide, every year. It is administered either through total intravenous anaesthesia, using only intravenous agents, or through inhalational anaesthesia, using volatile anaesthetic agents. The debate on how this affects postoperative patient outcome is ongoing, despite an abundance of published trials. The relevance of this topic has grown by the increasing concern about the contribution of anaesthetic gases to the environmental impact of surgery. We aimed to summarise all available evidence on relevant patient outcomes with total intravenous anaesthesia versus inhalational anaesthesia.

**Methods:**

In this systematic review and meta-analysis, we searched PubMed/Medline, Embase and Cochrane Central Register of Controlled trials for works published from January 1, 1985 to August 1, 2023 for randomised controlled trials comparing total intravenous anaesthesia using propofol versus inhalational anaesthesia using the volatile anaesthetics sevoflurane, desflurane or isoflurane. Two reviewers independently screened titles, abstracts and full text articles, and assessed risk of bias using the Cochrane Collaboration tool. Outcomes were derived from a recent series of publications on consensus definitions for Standardised Endpoints for Perioperative trials (StEP). Primary outcomes covered mortality and organ-related morbidity. Secondary outcomes were related to anaesthetic and surgical morbidity. This study is registered with PROSPERO (CRD42023430492).

**Findings:**

We included 317 randomised controlled trials, comprising 51,107 patients. No difference between total intravenous and inhalational anaesthesia was seen in the primary outcomes of in-hospital mortality (RR 1.05, 95% CI 0.67–1.66, 27 trials, 3846 patients), 30-day mortality (RR 0.97, 95% CI 0.70–1.36, 23 trials, 9667 patients) and one-year mortality (RR 1.14, 95% CI 0.88–1.48, 13 trials, 9317 patients). Organ-related morbidity was similar between groups except for the subgroup of elderly patients, in which total intravenous anaesthesia was associated with a lower incidence of postoperative cognitive dysfunction (RR 0.62, 95% CI 0.40–0.97, 11 trials, 3834 patients) and a better score on postoperative cognitive dysfunction tests (standardised mean difference 1.68, 95% CI 0.47–2.88, 9 trials, 4917 patients). In the secondary outcomes, total intravenous anaesthesia resulted in a lower incidence of postoperative nausea and vomiting (RR 0.61, 95% CI 0.56–0.67, 145 trials, 23,172 patients), less emergence delirium (RR 0.40, 95% CI 0.29–0.56, 32 trials, 4203 patients) and a higher quality of recovery score (QoR-40 mean difference 6.45, 95% CI 3.64–9.25, 17 trials, 1835 patients).

**Interpretation:**

The results indicate that postoperative mortality and organ-related morbidity was similar for intravenous and inhalational anaesthesia. Total intravenous anaesthesia offered advantages in postoperative recovery.

**Funding:**

Dutch Society for Anaesthesiology (10.13039/100008910NVA).

**Research in context**

**Evidence before this study**

General anaesthesia is maintained either through total intravenous anaesthesia, using only intravenous agents, or through inhalational anaesthesia, using volatile agents. The debate on how this affects postoperative outcome is ongoing, despite an abundance of published trials. The relevance of this debate has grown by an increasing concern about the carbon footprint of surgical care. We searched PubMed, Embase and Web of Science, from January 1, 1985 to August 1, 2023, with no language restrictions, for systematic reviews and meta-analyses using search terms for “volatile anaesthetics” and “propofol”. Results were generally confined to specific domains, like ambulatory, cardiac or neurosurgery, or to specific outcomes, like cognition, pain or surgical complications. Most reviews were performed between five and twenty years ago, while in the last five years alone over 100 eligible randomised controlled trials have been published on this topic. In addition, a core outcome set for perioperative trials was recently established by a group of experts using a Delphi validation process. This outcome set has not been used in any previous systematic review.

**Added value of this study**

We included 317 RCTs in the meta-analysis comprising 51,107 patients. Regarding the primary outcomes of mortality and organ-related morbidity, no difference between total intravenous anaesthesia and inhalational anaesthesia was found. In the subgroup of elderly adults, total intravenous anaesthesia may be associated with reduced short-term cognitive dysfunction after surgery. Overall, quality of recovery was higher after receiving total intravenous anaesthesia and the incidence of emergence delirium and postoperative nausea and vomiting was decreased.

**Implications of all the available evidence**

Total intravenous anaesthesia using propofol and inhalational anaesthesia using volatile anaesthetics are both safe and efficacious. The quality of recovery was higher after total intravenous anaesthesia, reflected by lower incidences of emergence delirium and nausea and vomiting.

## Introduction

General anaesthesia is administered to more than 300 million surgical patients worldwide, every year.^1^ It is maintained either through total intravenous anaesthesia (TIVA), by infusion of propofol, or through inhalational anaesthesia (IA), by inhalation of the volatile anaesthetics desflurane, isoflurane or sevoflurane. Anaesthetic agents have been attributed with organ-protective qualities that could help prevent adverse perioperative events. Acute organ injury in the perioperative period is associated with a 30-day mortality of roughly 1.5% in Europe and North-America.^2^, ^3^, ^4^ In the 1970’s, experimental evidence suggested that volatile anaesthetics may protect against perioperative organ ischaemia-reperfusion injury.^5^^,^^6^ These findings were later substantiated by animal models and initial clinical research.^7^ This confirmed IA as the prevalent way of providing anaesthesia and still influences practice today, in which up to 80% of anaesthesia is maintained using volatile anaesthetics.^8^, ^9^, ^10^ Propofol was introduced in the 1990’s and enables anaesthesia using only intravenous agents. Similar to IA, propofol-based TIVA has been linked to organ-protective qualities. These are attributed to anti-inflammatory and immunomodulatory effects^11^ which may influence pain, cognition and even cancer recurrence after surgical resection.^12^ The potential benefit of anaesthetic agents has been the subject of many randomised controlled trials (RCTs) that compare TIVA and IA. To date, the largest RCTs have failed to establish the superiority of either approach concerning the two most prominent claims: the organ-protective effects of IA in cardiac surgery^13^ and the inhibitory effects of TIVA on cancer recurrence after surgical resection.^14^^,^^15^ Meanwhile, the debate about potential benefit in other relevant clinical outcomes is ongoing.

The importance of this debate has grown in recent years, not because of new medical insights, but by a growing concern about the environmental impact of anaesthetic gases, which are highly potent greenhouse gases (GHGs).^16^, ^17^, ^18^ Healthcare aims to promote health but paradoxically has become an important contributor to pollution and climate change, responsible for 4%–10% of global GHG emissions.^19^^,^^20^ Anaesthetic gases account for up to 63% of operating theatre GHG emissions and contribute roughly 3% of the carbon footprint of the entire health sector,^21^^,^^22^ while the carbon footprint of TIVA is negligible.^23^^,^^24^ Real-world data show that switching from IA to TIVA reduces carbon emissions of anaesthetic care by over 95%.^25^ Reducing the emissions of volatile anaesthetics is an important step in reaching the goals of a carbon net-zero healthcare.^26^ However, caution is warranted as the choice of anaesthetic may impact patient outcomes. Therefore, a thorough assessment is needed to quantify the differences between TIVA and IA.

To summarise the abundance of clinical trials, several meta-analyses have been performed. These were often limited to specific clinical domains, like ambulatory surgery,^27^ cardiac surgery^28^ or neurosurgery,^29^ and those that did cover a general surgical population were mostly confined to specific outcomes, like mortality,^30^ cognitive decline,^31^ postoperative pain^32^ or emergence delirium.^33^ Most of these reviews were performed several years ago, while in the last five years alone over 100 eligible RCTs have been published. Another recent development is the publication of a core and extended outcome set for perioperative trials by the Standardised Endpoints in Perioperative medicine (StEP) expert groups.^34^ This set has not been used in systematic reviews on this topic before and includes critical outcomes on every major organ system, complemented by patient-centred outcomes and resource utilization. The outcome set covers all important perioperative outcomes and creates a standardization that increases consistency of RCTs and validity of pooled results.

We conducted a systematic review and meta-analysis to summarise all available evidence from RCTs and enable informed decision-making on the use of TIVA and IA in anaesthetic practice.

## Methods

The protocol of this systematic review and meta-analysis was published in the PROSPERO registry (CRD42023430492). The Preferred Reporting Items for Systematic Reviews and Meta-Analyses (PRISMA) guidelines were used in the design and reporting.^35^

### Data sources

We searched PubMed/Medline, Embase and Cochrane Central Register of Controlled trials for works published from January 1, 1985 to August 1, 2023 (full search in Appendix 1). References of studies were screened for additional records. We did not search for unpublished studies through funding agencies, trial registries or meeting abstracts.

### Eligibility

We considered RCTs that included two or more groups undergoing a surgical procedure and compared different drug-regimes for anaesthesia maintenance, of which at least one was propofol-based TIVA and one IA using sevoflurane, desflurane or isoflurane. RCTs combining propofol and volatile anaesthetics during the maintenance phase of anaesthesia were excluded. Nitrous oxide was considered outside of the scope of this review. Nitrous oxide is a low-potency anaesthetic gas that is sometimes used as an adjuvant to propofol or volatile anaesthetics with little effect on postoperative outcome.^36^ RCTs were eligible for inclusion as long as the administered fraction of nitrous oxide was the same in both groups. The anaesthetic agents used for the induction of anaesthesia were also considered outside of the scope of this review. No language restrictions were applied.

### Outcomes

Primary outcomes were mortality and organ-related morbidity based on the Standardized Endpoints in Perioperative medicine (StEP) core outcome set.^34^ The main outcomes were 30-day and one-year all-cause mortality. We added in-hospital mortality because it was the most prevalent mortality outcome in the included RCTs. The remaining primary outcomes covered major cardiologic, pulmonary, renal and neurological morbidity. Postoperative cognitive dysfunction (POCD) was reported as either an incidence or a test score, which were collected and meta-analysed separately. Secondary outcomes were also based on the StEP outcome set and covered anaesthetic and surgical morbidity, including the Clavien-Dindo classification for adverse events^37^ and the QoR-40 quality of recovery questionnaire.^38^ Additional outcomes assessed efficiency, including the time to emerge from anaesthesia, hospital resource consumption and cost. Table 1 lists all 21 primary, 14 secondary and 13 efficiency outcome measures included in the meta-analysis (definitions of outcomes in Appendix 2). Due to the large number of prespecified outcomes, we chose to only report meta-analysis results that included at least five RCTs in the main text to improve overall readability of the results. Unabridged results are available in the Supplementary material.

**Table 1:** The 48 prespecified outcome measures.

Category	Outcome measures
Primary outcomes	
Mortality	Mortality in-hospital, mortality 30-day, mortality one-year.
Cardiovascular	Myocardial infarction, myocardial injury, cardiovascular death, non-fatal cardiac arrest, coronary revascularisation, major adverse cardiac event, pulmonary embolism, deep vein thrombosis, atrial fibrillation.
Pulmonary	Atelectasis, pneumonia, acute respiratory distress syndrome, pulmonary aspiration.
Neurological	Cerebrovascular accident/stroke, postoperative delirium, postoperative cognitive dysfunction.
Renal	Acute kidney injury, initiation of new renal replacement therapy.
Secondary outcomes	
Anaesthetic	Postoperative nausea and vomiting, emergence delirium/agitation upon emergence, postoperative shivering, awareness/accidental awakening, QoR-40 questionnaire, QoR-15 questionnaire, first pain <12 h postoperatively, pain 12–24 h postoperatively and pain >24 h postoperatively.
Surgical	Clavien-Dindo classification grades ≥3, major bleeding, surgical site infection, cancer recurrence, WHODAS questionnaire.
Efficiency outcomes	
Anaesthetic	Intraoperative opioid consumption, postoperative opioid consumption, time to extubation, time to awakening, time to follow simple instruction, time to respiratory recovery, time to recovery score, time to orientation, time in post-anaesthesia care unit.
General	Length of hospital stay, unplanned readmission or unplanned admission after day case surgery, unplanned intensive care unit admission, cost analysis.

Definitions are provided in Appendix 2.

### Data screening and extraction

All articles were independently screened on title, abstract and, if necessary, full text by two blinded reviewers out of JK, CG, WB, SS and LP. Conflicts were settled by discussion and, if necessary, by a third reviewer from the same group. The same authors assessed risk of bias and collected the data. Data were extracted on population, anaesthetic exposure and outcomes. Risk of bias was assessed for each RCT using the Cochrane Handbook for Systematic Reviews of Interventions tool.^39^ The covered domains were: random sequence generation, allocation concealment, blinding of participants and personnel, blinding of outcome assessment, incomplete outcome data, and selective reporting. Blinding of the attending anaesthetist was considered impossible due to practical differences of providing TIVA or IA. If other personnel involved was blinded to the group assignment, risk of bias in this domain was considered low.

We used the Grading of Recommendations Assessment, Development and Evaluation (GRADE) tool to appraise the certainty of evidence as either high, moderate, low or very low.^40^ Certainty assessments were based on the following GRADE domains: risk of bias, inconsistency, imprecision, indirectness, publication bias, and other considerations. Funnel plot asymmetry was assessed to detect potential publication bias.

### Data analysis and synthesis

For each prespecified outcome, a separate meta-analysis was performed using Review Manager version 5.4.0 (Cochrane Community, UK). We determined risk ratios (RR), mean differences (MD) and standardised mean differences (SMD), along with corresponding 95% confidence intervals (CI). A two-sided P-value of <0.05 was considered statistically significant. Because of clinical heterogeneity between the included RCTs (population, type of surgery, definition of outcome measures), a random-effects model was used for all meta-analysis calculations. We used the Mantel-Haenszel approach to derive risk ratios for binary incidence data, and the inverse-variance approach for continuous data. When continuous data were reported in different forms, i.e. either in sample means with standard deviation or medians with range or interquartile range, data were transformed using the methods suggested by Wan and colleagues (example calculation in Appendix 3).^41^

Our main subgroup analysis separated cardiac surgery versus non-cardiac surgery. Cardiac surgery is a small but distinct part of all surgical procedures. During cardiopulmonary bypass, the heart and all other organs are at high risk of ischaemia-reperfusion damage.^5^ Cardiac surgery is also the only field in which anaesthetic choice is incorporated in clinical guidelines (recommending IA)^42^ and in which the largest RCT has been performed (reporting equivalent outcomes for TIVA and IA).^13^ Therefore, it is in this setting that potential organ-protective advantages of volatile anaesthetics would be most visible.

Other subgroup analyses compared outcomes between i. TIVA versus each of the individual volatile agents (sevoflurane, desflurane and isoflurane), ii. the age of the included population (children <18, adults or elderly adults) and iii. the type of surgery (pulmonary, intracranial or vascular surgery). We conducted a post hoc sensitivity analysis for the primary outcomes in which only RCTs were included categorised as low risk of bias in every one of the six domains. No trial sequential analysis was performed because evidence-based thresholds for clinical importance were not available for the included outcomes.

### Patient and public perspective

The findings were discussed by one of the authors (JK) at a dedicated 45-min meeting of the Amsterdam UMC client council. This council consists of patients and former patients with relevant (occupational) experience.^43^ The aim of the council is to protect and promote the quality of healthcare in the broadest sense, and to provide the patient perspective. They are consulted by the hospital board on a wide range of hospital matters, and their advice is an official part of any significant decision-making process.

For this study, council members were asked about priorities in the perioperative period relevant to patients. This included perioperative safety and adverse events, but also the environmental impact of care, and whether this should be part of the regular shared-decision making process. Feedback from this meeting was used in the interpretation of the results and writing of the manuscript. Minutes of the meeting are provided in Appendix 4.

### Ethics statement

No ethical approval was required for this study.

### Role of the funding source

Funding was provided by the Dutch Society for Anaesthesiology. The funder had no role in the study design, data collection, data analysis, data interpretation, or writing of the report.

## Results

**Fig. 1:**
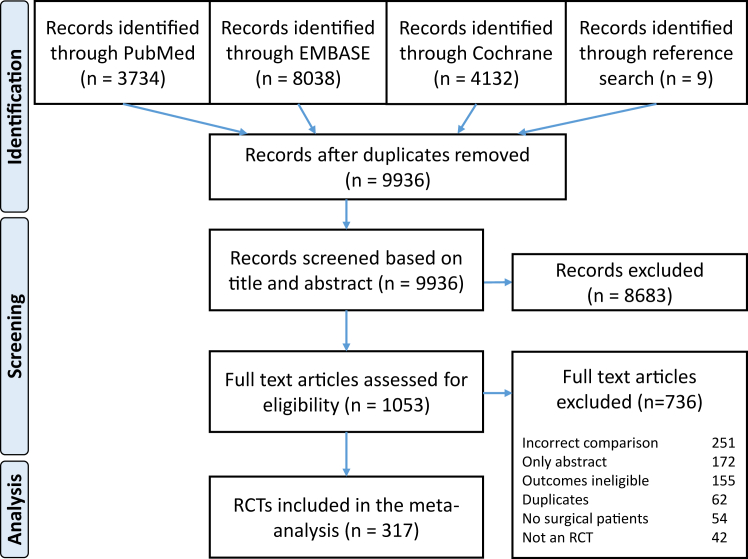
PRISMA flow chart of study selection.

Of 15,913 records identified, 5977 duplicates were excluded, 9936 titles and abstracts screened, and 1053 full text articles assessed. A total of 317 randomised controlled trials were included in the meta-analysis, comprising 51,107 patients (Fig. 1 and Table 2). The majority of RCTs covered non-cardiac surgery (267 RCTs, 84%), included adult patients (292 RCTs, 92%), and compared TIVA versus IA with sevoflurane (211 RCTs, 67%), desflurane (67 RCTs, 21%) and/or isoflurane (57 RCTs, 18%). The first RCT was published in 1988 and the number of RCTs has since exponentially increased, with 108 RCTs (34%) published in the last five years (2019–2023). Risk of bias was overall moderate, mainly due to uncertainty about blinding. A full list of RCTs, including risk of bias assessment, is provided in Appendix 8. Out of 48 prespecified outcome measures, 35 were reported by five or more RCTs, including twelve primary outcomes, eleven secondary outcomes and twelve efficiency measures. Results for outcomes reported in less than five RCTs are provided in Appendix 7.

**Table 2:** Characteristics of randomised controlled trials (RCTs) included in meta-analysis.

Characteristic	No of RCTs	No of patients
Total No of RCTs	317	51,107
Type of anaesthetic		
Sevoflurane	211	13,942
Desflurane	67	3174
Isoflurane	57	3301
Unspecified	5	5074
Propofol	317	26,046
Population		
Adults (including elderly)	292	49,093
Children	25	2014
Elderly adults	25	7533
Year of publication		
1985–1993	3	136
1994–1998	18	1169
1999–2003	20	2646
2004–2008	36	7966
2009–2013	45	6051
2014–2018	87	10,717
2019–August 1, 2023	108	22,422
Type of surgery		
Cardiac	50	12,113
Non-cardiac	267	38,994

A full list of included trials is provided in Appendix 8.

### Primary outcomes

**Fig. 2:**
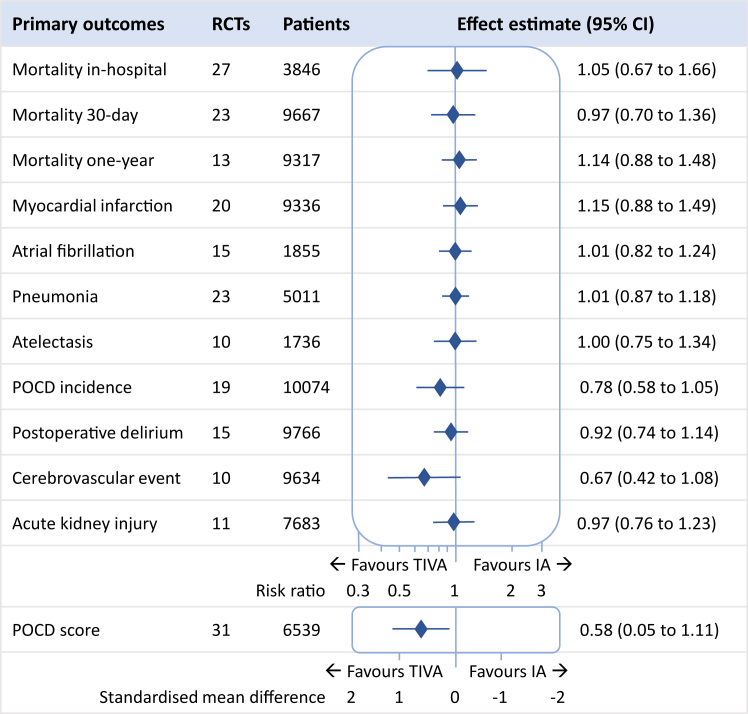
Primary mortality and morbidity findings. Effect estimates are risk ratios, except postoperative cognitive dysfunction (POCD) score which is a standardised mean difference (SMD). TIVA: total intravenous anaesthesia, IA: inhalational anaesthesia.

Fig. 2 and Table 3 summarise the overall results for the primary outcomes. Forest plots are provided in Appendix 10. The meta-analyses revealed similar results between TIVA and IA for in-hospital mortality (RR 1.05, 95% CI 0.67–1.66, 27 trials, 3846 patients, low certainty), 30-day mortality (RR 0.97, 95% CI 0.70–1.36, 23 trials, 9667 patients, moderate certainty) and one-year mortality (RR 1.14, 95% CI 0.88–1.48, 13 trials, 9317 patients, low certainty). Similar mortality findings were seen for the main subgroup analysis of cardiac versus non-cardiac surgery, resulting in risk ratios for, respectively, in-hospital mortality of 1.01 (95% CI 0.45–2.27) versus 1.07 (95% CI 0.62–1.86), 30-day mortality of 0.95 (95% CI 0.67–1.35) versus 1.24 (95% CI 0.43–3.59) and one-year mortality of 1.17 (95% CI 0.95–1.45) versus 1.43 (0.73–2.77) (see Appendix 9 for full subgroup analyses).

**Table 3:** Primary mortality and morbidity findings.

Primary outcome measures	Incidence TIVA	Incidence IA	Effect estimate (95% CI)	Risk difference (95% CI) per 100 TIVA cases	Preference	Certainty	I^2^	P-value
Mortality								
Mortality in-hospital	34/1792	34/2054	1.05 (0.67–1.66)	0 cases (−1 to 0)	None	Low	0%	0.82
Mortality 30-day	66/4726	67/4941	0.97 (0.70–1.36)	0 cases (−1 to 0)	None	Moderate	0%	0.87
Mortality one-year	231/4683	188/4634	1.14 (0.88–1.48)	+1 cases (0 to +2)	None	Low	23%	0.32
Morbidity—cardiovascular								
Myocardial infarction	118/4611	105/4725	1.15 (0.88–1.49)	0 cases (0 to +1)	None	Moderate	0%	0.31
Atrial fibrillation	142/883	151/972	1.01 (0.82–1.24)	0 cases (−3 to +2)	None	Moderate	0%	0.96
Morbidity—pulmonary								
Pneumonia	269/2420	299/2591	1.01 (0.87–1.18)	0 cases (−2 to +1)	None	Moderate	0%	0.86
Atelectasis	91/778	96/958	1.00 (0.75–1.34)	0 cases (−2 to +3)	None	Moderate	6%	0.99
Morbidity—neurological								
POCD score	31 RCTs	6539 patients	0.58 (0.05–1.11)^a^	Not applicable	TIVA	Very low	99%	0.03
Subgroup								
Elderly	9 RCTs	4917 patients	1.68 (0.47–2.88)^a^	Not applicable	TIVA	Low	100%	0.006
Non-elderly	22 RCTs	1622 patients	0.16 (−0.11 to 0.43)^a^	Not applicable	None	Very low	86%	0.32
POCD incidence	359/5007	529/5067	0.78 (0.58–1.05)	−4 cases (−10 to +1)	None	Very low	77%	0.11
Subgroup								
Elderly	172/1895	369/1939	0.62 (0.40–0.97)	−9 cases (−14 to −3)	TIVA	Moderate	81%	0.04
Non-elderly	187/3112	160/3128	1.07 (0.78–1.47)	+3 cases (−5 to +10)	None	Very low	54%	0.68
Postoperative delirium	310/4889	337/4877	0.92 (0.74–1.14)	0 cases (−2 to +1)	None	Moderate	41%	0.49
Cerebrovascular event	28/4815	43/4819	0.67 (0.42–1.08)	0 cases (0–0)	None	Moderate	0%	0.10
Morbidity—renal								
Acute kidney injury/AKI	169/3832	175/3851	0.97 (0.76–1.23)	0 cases (−2 to +2)	None	Moderate	27%	0.78

Risk difference is expressed per 100 patients. All calculations are performed using a random-effects model. Outcomes that include at least five RCTs are reported, all other outcomes are listed in Appendix 7. Unabridged subgroup analyses are provided in Appendix 9. Forest plots are provided in Appendix 10. GRADE certainty of evidence levels range from high to moderate, low and very low. TIVA: total intravenous anaesthesia, IA: inhalational anaesthesia.

a Effect estimates are risk ratio, except postoperative cognitive dysfunction (POCD) score which is standardised mean difference.

For organ-related morbidity—cardiovascular, pulmonary, neurological and renal—no differences were identified between TIVA and IA, except for postoperative cognitive dysfunction (POCD), for which patients receiving TIVA had better test scores (standardised mean difference 0.63, 95% CI 0.08–1.18, 29 trials, 6355 patients, very low certainty).

The subgroup analysis revealed that POCD test scores were only significantly better for the subgroup of elderly patients receiving TIVA versus IA (standardised mean difference 1.68, 95% CI 0.47–2.88, 9 trials, 4917 patients, low certainty). For RCTs that reported a nominal POCD incidence, the subgroup of elderly patients had a lower risk of POCD after receiving TIVA (RR 0.62, 95% CI 0.40–0.97, 11 trials, 3834 patients, low certainty, risk difference of 9 cases per 100 patients). For the remaining neurological outcomes—postoperative delirium and stroke—the risk did not differ between TIVA and IA for the subgroup of elderly patients.

For all other primary outcomes, subgroup analyses for population age, type of volatile anaesthetic or type of surgery did not deviate from the overall results (see Appendix 9 for full subgroup analyses). The sensitivity analysis in which only RCTs at low risk of bias were included did not reveal a difference between TIVA and IA in any of the primary outcomes.

### Secondary outcomes

**Fig. 3:**
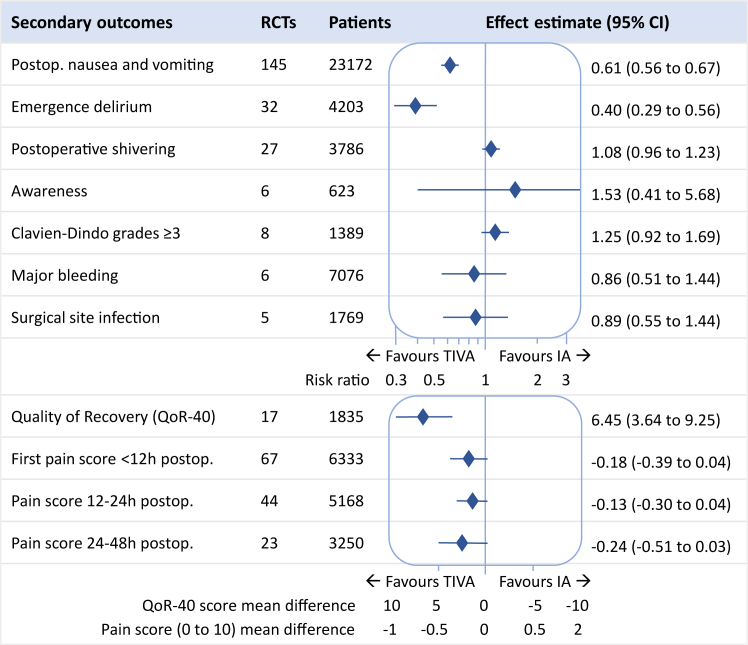
Secondary morbidity findings. Effect estimates are risk ratio, except QoR-40 score and pain scores which are mean differences (MD). TIVA: total intravenous anaesthesia, IA: inhalational anaesthesia.

The secondary outcomes covered anaesthetic and surgical morbidity. For most outcomes, no difference was seen between TIVA and IA (see Fig. 3 and Table 4). A difference was seen in favour of TIVA with regard to postoperative nausea and vomiting (RR 0.61, 95% CI 0.56–0.67, 145 trials, 23,172 patients, low certainty, risk difference of 11 cases per 100 patients) and emergence delirium (RR 0.40, 95% CI 0.29–0.56, 32 trials, 4203 patients, low certainty, risk difference of 17 cases per 100 patients). In addition, patients receiving TIVA scored higher on a quality of recovery questionnaire (QoR-40 mean difference 6.45, 95% CI 3.64–9.25, 17 trials, 1835 patients, low certainty).

**Table 4:** Secondary morbidity findings.

Secondary outcome measures	Incidence TIVA	Incidence IA	Effect estimate (95% CI)	Risk difference (95% CI) per 100 TIVA cases	Preference	Certainty	I^2^	P-value
Morbidity—anaesthetic and surgical								
Postop. nausea and vomiting	2629/12,158	3244/11,014	0.61 (0.56–0.67)	−11 cases (−13 to −9)	TIVA	Low	53%	<0.001
Subgroup								
Cardiac surgery	20/175	23/175	0.86 (0.50,1.49)	−1 cases (−7 to +5)	None	Moderate	0%	0.60
Non-cardiac	2609/11,983	3221/10,839	0.61 (0.56,0.66)	−11 cases (−13 to −9)	TIVA	Low	54%	<0.001
Emergence delirium	139/2097	400/2106	0.40 (0.29–0.56)	−17 cases (−24 to −11)	TIVA	Low	59%	<0.001
Subgroup								
Children (<18)	44/495	177/499	0.27 (0.15–0.50)	−27 cases (−44 to −9)	TIVA	Low	63%	<0.001
Adults (≥18)	95/1602	223/1607	0.50 (0.34–0.73)	−12 cases (−18 to −6)	TIVA	Low	51%	<0.001
Postoperative shivering	333/1820	342/1966	1.08 (0.96–1.23)	+1 cases (−1 to +3)	None	Moderate	0%	0.21
Awareness	5/312	3/311	1.53 (0.41–5.68)	0 cases (−1 to + 2)	None	Very low	0%	0.52
QoR-40/Quality of Recovery	17 RCTs	1835 patients	6.45 (3.64–9.25)^a^	Not applicable	TIVA	Low	95%	<0.001
Subgroup								
Cardiac surgery	1 RCTs	95 patients	0.95 (−6.41,8.31)^a^	Not applicable	None	Low	NA	0.80
Non-cardiac	16 RCTs	1740 patients	6.74 (3.85,9.63)^a^	Not applicable	TIVA	Low	95%	<0.001
First pain score <12 h	67 RCTs	6333 patients	−0.18 (−0.39 to 0.04)^a^	Not applicable	None	Very low	95%	0.10
Pain score 12–24 h	44 RCTs	5168 patients	−0.13 (−0.30 to 0.04)^a^	Not applicable	None	Very low	95%	0.14
Pain score 24–48 h	22 RCTs	3250 patients	−0.24 (−0.51 to 0.03)^a^	Not applicable	None	Very low	97%	0.09
Clavien-Dindo grades ≥3	67/607	67/782	1.25 (0.92–1.69)	+1 cases (−1 to +3)	None	Moderate	0%	0.15
Major bleeding	71/3489	77/3587	0.86 (0.51–1.44)	−1 cases (−3 to +1)	None	Moderate	28%	0.56
Surgical site infection	31/886	35/883	0.89 (0.55–1.44)	−1 cases (−2 to +1)	None	Moderate	0%	0.64

Risk difference is expressed per 100 patients. All calculations are performed using a random-effects model. Outcomes that include at least five RCTs are reported here, all other outcomes are listed in Appendix 7. Unabridged subgroup analyses are provided in Appendix 9. Forest plots are provided in Appendix 10. GRADE certainty levels range from high to moderate, low and very low. TIVA: total intravenous anaesthesia, IA: inhalational anaesthesia.

a Effect estimates are risk ratio, except QoR-40 score and pain scores (0–10 scale) which are mean differences.

The subgroup analysis found that emergence delirium was more common in children, and that the difference in favour of TIVA was much more distinct in this population (TIVA versus IA in children 44/495 (9%) versus 177/499 (35%), risk difference of 27 cases per 100 patients).

### Efficiency outcomes

The full efficiency results are listed in Appendix 6. The meta-analysis revealed no difference between TIVA and IA with regard to the time it took to emerge from anaesthesia, the time until tracheal extubation, the length of hospital stay and the rate of hospital readmission or unplanned admission after day case surgery. IA was associated with a lower consumption of intraoperative opioids (standardised mean difference 0.35, 95% CI 0.18–0.52, 80 trials, 11,309 patients), while postoperative opioid consumption was similar for TIVA and IA (standardised mean difference 0.02, 95% CI -0.15–0.20, 37 trials, 6614 patients).

The time it took patients to follow a simple instruction after anaesthesia was ceased was shorter after receiving IA in the desflurane subgroup (1.66 min, 95% CI 0.49–2.83), but not for sevoflurane and isoflurane (see subgroup analysis in Appendix 9). The time spent in de post-anaesthesia care unit was slightly shorter after TIVA (mean difference −1.59 min, 95% CI −3.15 to −0.03).

## Discussion

In this systematic review and meta-analysis of RCTs comparing total intravenous anaesthesia with inhalational anaesthesia, mortality and organ-related morbidity was similar between the two groups. TIVA offered several advantages in secondary outcomes, including a higher quality of recovery score (QoR-40 questionnaire), less postoperative nausea and vomiting and lower incidences of emergence delirium and cognitive dysfunction in elderly patients.

The large number of outcomes included in the analysis constitutes both benefits and risks. While it reduces the risk of missing relevant information, it also increases the chance of false positive findings. It is therefore crucial to note that identified differences between TIVA and IA should ideally be sufficiently large, clinically relevant and based on pathophysiological understanding.

The present meta-analysis totalled over 51,000 randomised patients. The main finding is that TIVA and IA are similarly safe and efficacious with regard to critical measures like mortality and organ-related morbidity. In the secondary outcomes, several differences between TIVA and IA were identified which were generally in favour of TIVA.

Only one difference was identified in the primary outcomes. This concerned the subgroup of elderly patients in the two POCD outcomes (test scores and incidence). These subgroups included a combined total of 6588 unique elderly patients and revealed that TIVA led to a reduced incidence of POCD and better POCD test scores compared to IA. Similar findings were recently reported by two large population-based studies^44^^,^^45^ and were hypothesized by a Cochrane review, although evidence at the time was not conclusive.^31^ An explanation of our findings may lie in the effects of volatile anaesthetics on neurophysiology, including caspase activation, apoptosis and β-amloid aggregation.^46^ Patients with pre-existing mild amnestic cognitive impairment may be particularly vulnerable.^47^ However, our findings are limited by several factors. First, different cognitive tests were pooled and tests were performed at different time points, ranging from 30 min to three months after surgery. The majority of POCD tests was performed within the first few postoperative days, meaning that is uncertain whether TIVA influences long term cognitive recovery. Second, the gold standard for diagnosing POCD is an extensive face-to-face neurophysiological assessment, which is often not feasible in practice. In the included RCTs, and in the real-world perioperative setting, simplified tests are used which are only weakly correlated with this gold standard.^48^

Patients that received TIVA scored higher on a validated quality of recovery questionnaire (QoR-40), which consists of the following five domains: physical comfort, emotional state, physical independence, psychological support and pain.^38^ The mean difference score of 6.45 is above the value previously established as clinically relevant.^49^ This finding may be explained by the lower incidences of emergence delirium and postoperative nausea and vomiting, which are reflected in this score. The anti-emetogenic effect of propofol has been known since its introduction in the 1990’s.^50^ Lower incidences of postoperative nausea and vomiting and emergence delirium have been suggested by previous systematic reviews.^33^^,^^51^ In the present review, the difference in emergence delirium was especially pronounced in children receiving TIVA (9%, 44/495) compared with children receiving IA (35%, 177/499), resulting in an average risk difference of 27 cases per 100 patients.

Reducing opioid use is a prominent ambition of modern medical practice. For both volatile anaesthetics and propofol, analgesic properties have been described that might influence postoperative opioid needs.^32^^,^^52^ In the present meta-analysis, postoperative opioid use was similar for TIVA and IA, as well as the postoperative pain scores. Contrarily, opioid use during anaesthesia was slightly lower for IA, although it is unclear whether this holds clinical relevance.

The liveliness of the ongoing debate about TIVA versus IA is illustrated by the large number of eligible RCTs published in the last five year alone (108 trials). The largest of these is the MYRIAD trial that tested the hypothesis that IA would reduce mortality in 5400 patients undergoing coronary-artery bypass grafting (CABG), but demonstrated equivalence for the primary outcome of one-year mortality and for all secondary outcomes.^13^

A recent meta-analysis compared mortality after TIVA versus IA in operating rooms and intensive care units and reported an increased mortality for TIVA.^30^ The difference in results may be explained by several issues in the analysis. First, the authors used a fixed-effects model in their meta-analysis, while a random-effects model would have been more appropriate considering the substantial clinical heterogeneity between the included RCTs. When a random-effects model was applied, statistical significance was lost (risk ratio 1.05, 95% CI 0.98–1.13, P = 0.17). Second, the subgroup analysis revealed that mortality was only increased in cardiac surgery patients, in which the entire mortality difference is accounted for by one outlier RCT with a one-year mortality of 25% after low-risk CABG in the TIVA group.^53^ In the US and the UK, one-year mortality averages below 3%.^13^^,^^54^ The authors only offer “poor adherence to cardiac medication” as explanation. If this trial was left out, the pooled mortality risk for the remaining 46 cardiac surgery RCTs would have been similar in both groups. Third, the aforementioned MYRIAD trial is missing from the analysis. This RCT would have doubled the number of included cardiac surgery patients in the meta-analysis and would have reiterated that mortality was similar for TIVA and IA. Several compelling letters have been written in response to this review,^54^^,^^55^ further emphasising the liveliness of the ongoing debate. In the present meta-analysis, no difference between TIVA and IA was identified with regard to the three mortality outcomes, even though the same outlier RCT has been included. This RCT did influence the overall results and may explain the slightly unbalanced one-year mortality risk ratio of 1.14 (95% CI 0.88–1.48) compared to in-hospital and 30-day mortality (risk ratio respectively 1.05 and 0.97). An extra analysis without this outlier resulted in a more balanced risk ratio of 1.06 (95% CI 0.78–1.44).

Our meeting with the local client council confirmed that the primary priority for patients is the safety of the anaesthetic care they receive. Another important aspect was comfort, as perioperative patients are often in a vulnerable position and may have no voice of their own. In this regard, the council considered the reduction of emergence delirium and postoperative nausea and vomiting as significant advantages of TIVA. In addition, environmental sustainability was seen as a core value of high-quality healthcare. Patients appreciated to be informed about the environmental impact of the care they receive and feel these considerations should be a part of the shared decision-making process.

Previous research showed that the sustainable healthcare agenda remains largely unknown to patients and the public.^56^ Patients focus mainly on their health goals, but when consulted on the issue, made it clear that the environment mattered to them. They wanted to make informed decisions on this topic, although only 8% reported being engaged in shared-decision making about their anaesthetic care.^57^

IA has some inherent advantages that were outside the scope of this review. The main one is that IA does not require intravenous (IV) access. This allows mask induction of anaesthesia in individuals in whom placing IV access is complicated, for example in children. Similarly, IA can be an important safety option to deliver anaesthesia when IV access is accidentally lost intraoperatively. IA triggers alarms from the ventilator, whereas TIVA requires the anaesthesia team to be alert for problems with IV administration. The ventilator reports the concentration of the anaesthetic in the exhaled air, which is a useful indication of the actual effect size concentration in the patient’s circulation. A similar measurement does not exist for TIVA. It is important to add that none of the above are absolute indications that could not be substituted by TIVA, and the use of IA may be largely dictated by familiarity and habit.^58^ Physicians unfamiliar with TIVA may be uncomfortable with its use and concerned about adverse events, like awareness. To resolve this, a recent study tested the effects of skills training in TIVA, a combination of theory and bedside supervision. This significantly increased the uptake of TIVA and reduced anaesthetic gas use by 90%.^59^

Choices in healthcare are traditionally based on aspects related to quality, safety and cost. We believe that environmental sustainability should be considered as an additional pillar, especially when quality, safety and cost are similar between two care options. In sustainable development, the ‘triple bottom line’ is used, which consists of people, planet and profit. For people (patients), the findings of this meta-analysis suggest that TIVA and IA are similarly safe and efficacious. With regard to the planet, TIVA may be preferred over IA because currently available volatile anaesthetics are all potent greenhouse gases.^17^^,^^18^ The global warming potential on a 20-year horizon (GWP_20_) represents the relative impact to CO_2_, which for sevoflurane, isoflurane and desflurane is, respectively, 505, 1920 and 6930.^60^ Life cycle assessments (LCAs), the gold standard to assess the environmental impact of an entire process (cradle-to-grave), established that switching from IA to TIVA can reduce anaesthesia-related carbon emissions by 99%.^61^ When anaesthetic gases are used, they account for the majority of the carbon footprint of surgical care.^22^^,^^62^ The same LCA found that on other environmental outcomes (ecotoxicity, carcinogenic effects, etc.), the impact of anaesthesia is small for both TIVA and IA.^62^ Concerning profit, a recent analysis suggested that TIVA may be associated with an increased drug expenditure of about 1.10 EUR per hour (1.20 USD or 0.95 GPB).^63^ However, when the protective effects of TIVA were added to the analysis, e.g. the lower risk of nausea and vomiting, TIVA was shown to be cost-saving.^64^

Climate crisis mitigation policy has been formulated in the European Union to ban desflurane, the most potent GHG anaesthetic, and similar policy has already come into effect in Scotland.^65^^,^^66^ The continued availability of other volatile anaesthetics is important since there may always remain valid arguments for its use in clinical practice. Since current inhalational anaesthetics all have substantial environmental effects, future pharmacological research could focus on developing a molecule that does not have significant global warming potential. Meanwhile, adopting a sustainable approach that reserves volatile anaesthetics to indicated situations may best prevent further regulation.

The current meta-analysis is limited by prevailing heterogeneity among the large number of included outcomes and RCTs, which were performed over an extensive period of time and covered various surgical disciplines. Disparities in induction medication and co-analgesics were common, and different RCTs sometimes assessed outcomes at different time points. Several measures were taken to address the heterogeneity, including the use of random-effects models, subgroup analyses and GRADE assessment for certainty of evidence. For this assessment, funnel plots were visually assessed for asymmetry, which can be difficult, especially when the number of included studies is low. Strengths of the current meta-analysis include the extensive list of assessed outcomes that follow the StEP core outcome set. This enables comparison with future findings and can inform clinical decision-making.

In conclusion, this systematic review and meta-analysis found that postoperative mortality and organ-related morbidity was similar between patients receiving total intravenous anaesthesia versus inhalational anaesthesia. Total intravenous anaesthesia offered advantages in postoperative recovery, including a higher quality of recovery score (QoR-40 questionnaire), less nausea and vomiting and lower incidences of emergence delirium and postoperative cognitive dysfunction in elderly patients.

The overall results suggest that TIVA and IA are both safe and efficacious. This means that secondary considerations may be taken into account, including the environmental impact of volatile anaesthetics.

## Contributors

All authors were involved in the design of the study. JK, JH and NSW conceived the idea for the systematic review. JK, CG, WB, SS and LP screened records, extracted and analysed data, and assessed risk of bias. JK drafted the initial manuscript. NSW, JH, MH and SR provided critical revisions of the manuscript, and NSW supervised the project. JK and NSW accessed and verified the data. JK and NSW are the guarantors and attest that all authors meet authorship criteria and that no others have been omitted.

## Data sharing statement

The corresponding author (Jasper Kampman) can be contacted for any requests to share all collected data at j.m.kampman@amsterdamumc.nl.

## Declaration of interests

NSW and SR are both frequent speakers at public and private events on sustainability in healthcare, for which they have received travel reimbursements, but never any other financial payment. NSW chairs the Sustainability Taskforce of the Dutch Society for Anaesthesiology and is a member of the Sustainability Committee of the European Society for Anaesthesiology and Intensive Care (ESAIC). He is a section editor (anaesthesiology and intensive care) for the Amsterdam Medical Student Journal (AMSj). SR is a member of the advisory committee about sustainability at the University of Amsterdam. MH is section editor for Anaesthesia & Analgesia, Journal of Clinical Medicine and Frontiers of Physiology, and has received research support and honorariums from BBraun, Fresenius, IDD Pharma and PAION. The other authors declare no relationships or activities that could appear to have influenced the submitted work.
