# Supplementary material for: Mortality and morbidity after total intravenous anaesthesia versus inhalational anaesthesia: a systematic review and meta-analysis
Source: eClinicalMedicine. 2024 May 14;72:102636. doi: 10.1016/j.eclinm.2024.102636 (PMC11106536; doi:10.1016/j.eclinm.2024.102636)

**Supplementary Content**

*Title*

Mortality and morbidity after total intravenous anaesthesia versus inhalational anaesthesia: a systematic review and meta-analysis

*Authors*

Jasper M Kampman, Jeroen Hermanides, Markus W Hollmann, Coenraad N Gilhuis, Wouter AH Bloem, Stefan Schraag, Lorenzo Pradelli, Sjoerd Repping, Nicolaas H Sperna Weiland

**Content**

**Appendix 1: Search strategy** page 2

**Appendix 2: Definition of outcomes**  page 3

**Appendix 3: Example calculation conversion tool** page 7

**Appendix 4: Translated minutes of the client council meeting** page 8

**Appendix 5: GRADE assessment substantiation** page 9

**Appendix 6: Meta-analysis results of efficiency outcomes with ≥5 RCTs** page 18

**Appendix 7: Meta-analysis results of outcomes including <5 RCTs** page 20

**Appendix 8: List of included RCTs, including risk of bias assessment** page 22

**Appendix 9: Subgroup analyses** page 43

**Appendix 10: Forest and funnel plot for each outcome** page 56

**Appendix 1: Search strategy**

**Search Pubmed / Medline**

(anaesthe*[tiab] OR anesthe*[tiab])

AND

(volatile[tiab] OR inhal*[tiab] OR gas[tiab] OR gases[tiab] OR (“Anesthetics, Inhalation”[MeSH Terms] OR “Anesthetics, Inhalation”[Pharmacological Action] OR "Anesthesia, Inhalation"[Mesh]) OR ("sevoflurane"[Supplementary Concept]) OR "Isoflurane"[Mesh] OR "desflurane"[Supplementary Concept] OR sevoflurane[tiab] OR isoflurane[tiab] OR desflurane[tiab])

AND

("Propofol"[Mesh] OR propofol[tiab])

AND

("randomized controlled trial"[ptyp] OR "randomised controlled trial"[tiab] OR "randomized controlled trial"[tiab] OR random*[tiab])

AND

("1985/01/01"[Date - Publication] : "2023/08/01"[Date - Publication])

**Search Cochrane**

| #1 anaesthe*:ti,ab OR anesthe*:ti,ab  #2 volatile:ti,ab OR inhal*:ti,ab OR gas:ti,ab OR gases:ti,ab OR sevoflurane:ti,ab OR isoflurane:ti,ab OR desflurane:ti,ab |
| --- |

#3 MeSH descriptor: [Anesthetics, Inhalation] explode all trees

#4 MeSH descriptor: [Isoflurane] explode all trees

#5 MeSH descriptor: [Desflurane] explode all trees

#6 MeSH descriptor: [Sevoflurane] explode all trees

#7: #2 OR #3 OR #4 OR #5 OR #6

#8 propofol:ti,ab

#9 MeSH descriptor: [Propofol] explode all trees

#10: #8 OR #9

#11 ("randomized controlled trial"):pt OR random*:ti,ab

#12: #1 AND #7 AND #10 AND #11

**Search Embase**

(anaesthe*:ti,ab OR anesthe*:ti,ab)

AND

(volatile:ti,ab OR inhal*:ti,ab OR gas:ti,ab OR gases:ti,ab

OR

‘inhalation anesthetic agent’/exp

OR sevoflurane/exp OR isoflurane/exp OR desflurane/exp

OR

‘inhalation anesthetic’:ti,ab OR sevoflurane:ti,ab OR isoflurane:ti,ab OR desflurane:ti,ab)

AND

(propofol/exp OR propofol:ti,ab)

AND

(("randomized controlled trial"):pt OR ("randomised controlled trial"):pt OR ("randomized controlled trial"):ti,ab OR ("randomised controlled trial"):ti,ab OR random*:ti,ab )

AND [1985-2023]/py

**Appendix 2: Definition of outcomes**

*Supplementary Table 1: Definitions of the 48 prespecified outcome measures.*

| Outcome | Definition |
| --- | --- |
| Primary outcomes – mortality and organ-related morbidity | |
| Mortality in-hospital | Incidence of all-cause mortality during the postoperative hospital stay. |
| Mortality 30-day | Incidence of all-cause mortality at 30 days postoperatively. |
| Mortality one-year | Incidence of all-cause mortality at 1 year postoperatively. |
| Myocardial infarction | Incidence of myocardial infarction with clinical evidence, time point of assessment and exact definition of outcome as described in the original RCT publication. |
| Myocardial injury | Incidence of myocardial injury not leading to myocardial infarction. This endpoint is generally assessed using biomarkers, e.g. troponin. The time point of assessment and exact definition of the endpoint are as described in the original RCT publication. |
| Cardiovascular death | Incidence of death due to cardiovascular causes, time point of assessment and definition as described in the original RCT publication. |
| Non-fatal cardiac arrest | Incidence of non-fatal cardiac arrest, time point of assessment and definition as described in the original RCT publication. |
| Coronary revascularisation | Incidence of coronary revascularisation, time point of assessment and definition as described in the original RCT publication. |
| Major adverse cardiac event (MACE) | Incidence of MACE, as defined and described in the original RCT publication. |
| Pulmonary embolism | Incidence of pulmonary embolism, time point of assessment and definition as described in the original RCT publication. |
| Deep vein thrombosis | Incidence of deep vein thrombosis, time point of assessment and definition as described in the original RCT publication. |
| Atrial fibrillation | Incidence of atrial fibrillation, time point of assessment and definition as described in the original RCT publication. |
| Atelectasis | Incidence of pulmonary atelectasis, time point of assessment and definition as described in the original RCT publication. |
| Pneumonia | Incidence of pneumonia, time point of assessment and definition as described in the original RCT publication. |
| Acute respiratory distress syndrome (ARDS) | Incidence of ARDS, time point of assessment and definition as described in the original RCT publication. |
| Pulmonary aspiration | Incidence of perioperative pulmonary aspiration, time point of assessment and definition as described in the original RCT publication. |
| Cerebrovascular accident / stroke | Incidence of cerebrovascular events, time point of assessment and definition as described in the original RCT publication. |
| Postoperative delirium | Incidence of postoperative delirium, time point of assessment and definition as described in the original RCT publication. |
| Postoperative cognitive dysfunction (POCD) | Incidence of POCD, reported as either POCD incidence or POCD test score. Time point of assessment of assessment and definition of POCD as described in the original RCT publication. |
| Acute kidney injury (AKI) | Incidence of AKI, time point of assessment and definition as described in the original RCT publication. |
| Renal replacement therapy | Initiation of new renal replacement therapy not present before the surgical procedure. |
| Secondary outcomes – anaesthetic and surgical morbidity | |
| Postoperative nausea and vomiting (PONV) | Incidence of PONV, time point of assessment and definition as described in the original RCT publication. |
| Emergence delirium | Incidence of agitation upon emergence, time point of assessment and definition as described in the original RCT publication. |
| Postoperative shivering | Incidence of shivering, time point of assessment and definition as described in the original RCT publication. |
| Awareness | Incidence of awareness (recall of specific intraoperative moments) , time point of assessment and definition as described in the original RCT publication. |
| Quality of Recovery (QoR-40) questionnaire | Postoperative score on the QoR-40 questionaire, time point of assessment as described in the original RCT publication. |
| Quality of Recovery (QoR-15) questionnaire | Postoperative score on the QoR-15 questionaire, time point of assessment as described in the original RCT publication. |
| First pain <12 hours | First documented pain score, at least within 12 hours of awakening. |
| Pain 12-24 hours | Earliest documented pain score that is between 12 and 24 hours of awaking from anaesthesia. |
| Pain 24-48 hours | Earliest documented pain score that is between 24 and 48 hours of awaking from anaesthesia. Long term pain scores were included based on a meta-analysis by Peng and collegues (PMID: 27636574) and Qiu and colleagues (PMID: 27506326) that demonstrated reduced pain at 24 hours postoperatively. |
| Clavien-Dindo | Number of patients with a Clavien-Dindo classification of three or higher, which constitutes a complication that requires a surgical, endoscopic or radiological intervention. |
| Major bleeding | Incidence of major bleeding of the surgical site, definition of ‘major’ as described in the original RCT publication. |
| Surgical site infection (SSI) | Incidence of SSI, definition as described in the original RCT publication. |
| Cancer recurrence | Incidence of cancer recurrence after resective surgery, time point of assessment and definition as described in the original RCT publication. |
| WHODAS questionnaire | World Health Orginasation Disability Assessment Schedule questionnaire score, time point of assessment as described in the original RCT publication. |
| Efficiency outcomes – Anaestethic and general efficiency | |
| Intraoperative opioid consumption | The total opioid consumption during anaesthesia, type of opioid and metric as reported by the original RCT publication. |
| Postoperative opioid consumption | The total opioid consumption in the postoperative period, type of opioid and length of the ‘postoperative period’ as reported by the original RCT publication. |
| Time to extubation | Time in minutes from anaesthetic administration stop to extubation. |
| Time to awakening | Time in minutes from anaesthetic administration stop to awakening, defined as either eye opening or emergence, whichever is reported by the original RCT publication, or whichever occurs first. |
| Time to follow simple instruction | Time in minutes from anaesthetic administration stop to responding to a verbal command (e.g. hand movement). |
| Time to respiratory recovery | Time in minutes from anaesthetic administration stop to spontaneous and sufficient respiration. |
| Time to recovery score | Time in minutes from anaesthetic administration stop to reaching a recovery score (e.g. Aldrete score ≥9). |
| Time to orientation | Time in minutes from anaesthetic administration stop to orientation. Definition of orientation as described in the original RCT publication, which was generally either patient name or current date. |
| Time in PACU | Total duration in minutes in the post-anaesthesia care unit. |
| Length of hospital stay | Total duration in days in the hospital postoperatively until discharge. |
| Unplanned readmission | Number of patients that are readmitted (unplanned) after hospital discharge or admitted (unplanned) after day case surgery. |
| Unplanned ICU admission | Number of patients that are admitted to the ICU (unplanned) during the postoperative hospital stay. |
| Cost analysis | The financial cost of the perioperative period, time point of assessment and definition as described in the original RCT publication. |

**Appendix 3: Example calculation conversion tool**

Wan and colleagues (*BMC Med Res Methodol,* 2004, PMID: 25524443) suggested a conversion tool to estimate the sample mean and standard deviation of data reported as a median with either the interquartile range or the minimum and maximum, along with the sample size.

Below is an example calculation of one of the RCTs included in the meta-analysis.

RCT: Chang and colleagues, 2022, JAMA Otolaryngol Head Neck Surg (PMID:36107412)

Outcome: Length of hospital stay

Results VA arm: Median 15 days, interquartile range(Q1 to Q3) 14 to 18

Results TIVA arm: Median 16 days, interquartile range 14 to 22

Sample size (n): Total 70 patients, 35 patients in each arm

**Calculation**

Mean estimation: (Q1 + median + Q3) / 3

Standard deviation: (Q3 - Q1) / (2 * Φ^-1^((n - 0.375) / (n + 0.25))

**Example calculation**

VA arm

Mean estimation: (14 + 15 + 18) / 3 = 15.67

Standard deviation: (18 - 14) / (2 * Φ^-1^((35 - 0.375) / (35 + 0.25)) = 3.09

TIVA arm

Mean estimation: (14 + 16 + 22) / 3 = 17.33

Standard deviation: (22 - 14) / (2 * Φ^-1^((35 - 0.375) / (35 + 0.25)) = 6.18

Φ^-1^ (z) is the inverse function of the cumulative distribution of the standard normal distribution, which means it is the upper zth percentile of the standard normal distribution.

**Appendix 4: Translated summary of minutes from the client council meeting**


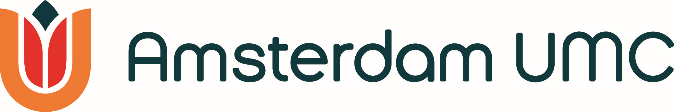


**Client council meeting Amsterdam UMC**

Date & time: 18-09-2023 | 17:15-18:00

Place: AMC – E2-140

Present: Bart, Bauco, Connie, Ellen (chair), Johan, Lizet (vice-chair), Steven, Margret (secretary)

Guest: Jasper Kampman – resident anaesthesiologist, PhD candidate

After a short introduction, Jasper proceeds with a presentation on the results of his research which concerns sustainability as a pillar on which medical decisions can be based, besides the established pillars in the Dutch healthcare system of quality, affordability and accessibility.

The goal of this meeting was to collect the perspective of a representative patient body on the research results, and to determine what place sustainability has in the shared-decision making process.

During the presentation, the different environmental impacts of inhalational anaesthesia (IA) and total intravenous anaesthesia (TIVA) were explained and that the goal of the current research was to quantify the impact on postoperative patient outcome (i.e. patient safety).

The subsequent discussion focused on three themes: i. sustainability as an aspect on which to base medical decisions, ii. patient involvement in the shared-decision making process regarding the sustainability of care options, and iii. the implications of the beneficial effects of TIVA in our secondary outcomes.

**Sustainability as a pillar in medical decisions**

The council members unanimously agree that sustainability is an important factor when deciding between care options, although it does rank behind safety and effectiveness.

**Sustainability as part of the shared-decision making process**

Because of the very large difference in environmental impact between VA and TIVA, council members are surprised that they have never heard about this, even though most have had (several) surgical interventions. On top of that, the majority of worldwide anaesthesia procedures is delivered using VA, without patients being involved in this discussion. This was seen as a lapse in the informed consent process.

**The implications of the secondary outcomes**

Perioperative patients are often vulnerable and may have no voice of their own. Incidences in the postoperative period, like pain but especially nausea and vomiting, significantly increase this vulnerability. Based on the secondary outcomes alone, without regarding sustainability, the council members clearly favored TIVA.

**Appendix 5: GRADE assessment substantiation**

*Supplementary Table 2: GRADE assessment for the primary outcomes that included at least five RCTs. Initial level of certainty for all outcomes is high since only randomized controlled trials were included. Green means no downgrade in certainty, orange means one level downgraded, red means two levels downgraded. Certainty levels range from high to moderate, low and very low. Abbreviations: RR risk ratio, CI confidence interval, SMD standardized mean difference. *Upgraded one level in certainty due to consistent and large effect estimate.*

| Primary outcome measures | Population | Risk of bias | Inconsistency | Indirectness | Imprecision | Publication bias | Certainty |
| --- | --- | --- | --- | --- | --- | --- | --- |
| Mortality |  |  |  |  |  |  |  |
| Mortality in-hospital | Overall | Some trials at moderate risk | No concerns | Diverse but representative populations | Large RR 95%CI range 0.71 to 1.69 | No concerns | Low |
|  | Cardiac | Some trials at moderate risk | No concerns | No concerns | Large RR 95%CI range 0.42 to 2.10 | No concerns | Low |
|  | Non-cardiac | Some trials at moderate risk | No concerns | Diverse but representative populations | Large RR 95%CI range 0.69 to 1.95 | No concerns | Low |
| Mortality 30-day | Overall | Some trials at moderate risk | No concerns | Diverse but representative populations | No concerns | No concerns | Moderate |
|  | Cardiac | Some trials at moderate risk | No concerns | Diverse but representative populations | No concerns | No concerns | Moderate |
|  | Non-cardiac | Some trials at moderate risk | No concerns | No concerns | Very large RR 95%CI range 0.43 to 3.59 | No concerns | Very low |
| Mortality one-year | Overall | Some trials at moderate risk | No concerns | Diverse but representative populations | No concerns | Some concerns | Low |
|  | Cardiac | Some trials at moderate risk | No concerns | No concerns | No concerns | No concerns | Moderate |
|  | Non-cardiac | Overall low risk of bias | High statistical heterogeneity | Diverse but representative populations | Large RR 95%CI range 0.69 to 2.97 | Some concerns | Very low |
| Morbidity – Cardiovascular |  |  |  |  |  |  |  |
| Myocardial infarction | Overall | Some trials at moderate risk | No concerns | No concerns | No concerns | No concerns | Moderate |
|  | Cardiac | Some trials at moderate risk | No concerns | No concerns | No concerns | No concerns | Moderate |
|  | Non-cardiac | Overall low risk of bias | No concerns | No concerns | Large RR 95%CI range 0.31 to 2.59 | No concerns | Moderate |
| Atrial fibrillation | Overall | Some trials at moderate risk | No concerns | No concerns | No concerns | No concerns | Moderate |
|  | Cardiac | Some trials at moderate risk | No concerns | No concerns | No concerns | No concerns | Moderate |
|  | Non-cardiac | No concerns | No concerns | Diverse but representative populations | Very large RR 95%CI range 0.20 to 4.88 | No concerns | Low |
| Morbidity – Pulmonary |  |  |  |  |  |  |  |
| Pneumonia | Overall | Some trials at moderate risk | No concerns | No concerns | No concerns | No concerns | Moderate |
|  | Cardiac | Some trials at moderate risk | No concerns | No concerns | No concerns | No concerns | Moderate |
|  | Non-cardiac | Some trials at moderate risk | No concerns | Diverse but representative populations | No concerns | Some concerns | Low |
| Atelectasis | Overall | Some trials at moderate risk | No concerns | Diverse but representative populations | No concerns | No concerns | Moderate |
|  | Cardiac | Overall low risk of bias | No concerns | No concerns | Very large RR 95%CI range 0.66 to 4.37 | No concerns | Low |
|  | Non-cardiac | Some trials at moderate risk | No concerns | Diverse but representative populations | No concerns | No concerns | Moderate |
| Morbidity – Neurological |  |  |  |  |  |  |  |
| Postoperative cognitive dysfunction score | Overall | Some trials at moderate risk | High statistical heterogeneity | Diverse but representative populations | No concerns | Some concerns | Very low |
|  | Cardiac | Some trials at moderate risk | High statistical heterogeneity | No concerns | Large SMD 95%CI range -1.82 to 1.56 | No concerns | Very low |
|  | Non-cardiac | Some trials at moderate risk | High statistical heterogeneity | Diverse but representative populations | No concerns | Some concerns | Very low |
|  | Elderly | Some trials at moderate risk | High statistical heterogeneity | Diverse but representative populations | No concerns | Some concerns | Low* |
|  | Non-elderly | Some trials at moderate risk | High statistical heterogeneity | Diverse but representative populations | No concerns | Some concerns | Very low |
| Postoperative cognitive dysfunction incidence | Overall | Some trials at moderate risk | High statistical heterogeneity | Diverse but representative populations | No concerns | Some concerns | Very low |
|  | Cardiac | Overall low risk of bias | High statistical heterogeneity | No concerns | Large RR 95%CI range 0.62 to 1.70 | Some concerns | Very low |
|  | Non-cardiac | Some trials at moderate risk | High statistical heterogeneity | Diverse but representative populations | No concerns | Some concerns | Very low |
|  | Elderly | Some trials at moderate risk | High statistical heterogeneity | Diverse but representative populations | No concerns | No concerns | Moderate* |
|  | Non-elderly | Some trials at moderate risk | High statistical heterogeneity | Diverse but representative populations | No concerns | Some concerns | Very low |
| Postoperative delirium | Overall | Overall low risk of bias | No concerns | No concerns | No concerns | Some concerns | Moderate |
|  | Cardiac | Overall low risk of bias | High statistical heterogeneity | No concerns | No concerns | Some concerns | Low |
|  | Non-cardiac | Overall low risk of bias | High statistical heterogeneity | Diverse but representative populations | No concerns | No concerns | Moderate |
| Cerebrovascular event / stroke | Overall | Some trials at moderate risk | No concerns | No concerns | No concerns | No concerns | Moderate |
|  | Cardiac | Some trials at moderate risk | No concerns | No concerns | No concerns | No concerns | Moderate |
|  | Non-cardiac | Some trials at moderate risk | No concerns | Diverse but representative populations | Large RR 95%CI range 0.33 to 2.04 | No concerns | Low |
| Morbidity – Renal |  |  |  |  |  |  |  |
| Acute kidney injury / AKI | Overall | Some trials at moderate risk | No concerns | No concerns | No concerns | No concerns | Moderate |
|  | Cardiac | Some trials at moderate risk | No concerns | No concerns | No concerns | No concerns | Moderate |
|  | Non-cardiac | No concerns | High statistical heterogeneity | Diverse but representative populations | No concerns | Some concerns | Low |

*Supplementary Table 3: GRADE assessment for the secondary outcomes that included at least five RCTs. Initial level of certainty for all outcomes is high since only randomized controlled trials were included. Green means no downgrade in certainty, orange means one level downgraded, red means two levels downgraded. Certainty levels range from high to moderate, low and very low. Abbreviations: RR risk ratio, CI confidence interval, MD mean difference, NA not applicable. *Upgraded one level in certainty due to consistent and large effect estimate.*

| Secondary outcome measures | Population | Risk of bias | Inconsistency | Indirectness | Imprecision | Publication bias | Certainty |
| --- | --- | --- | --- | --- | --- | --- | --- |
| Morbidity |  |  |  |  |  |  |  |
| Postoperative nausea and vomiting | Overall | Some trials at risk | High statistical heterogeneity | Diverse but representative populations | No concerns | Some concerns | Low* |
|  | Cardiac | Some trials at risk | No concerns | No concerns | No concerns | No concerns | Moderate |
|  | Non-cardiac | Some trials at risk | High statistical heterogeneity | Diverse but representative populations | No concerns | Some concerns | Low* |
| Emergence delirium | Overall | Some trials at risk | High statistical heterogeneity | Diverse but representative populations | No concerns | Some concerns | Low* |
|  | Cardiac | Some trials at risk | No concerns | No concerns | Large RR 95%CI range 0.36 to 1.38 | No concerns | Low |
|  | Non-cardiac | Some trials at risk | High statistical heterogeneity | Diverse but representative populations | No concerns | Some concerns | Low* |
|  | Children | Some trials at risk | High statistical heterogeneity | No concerns | No concerns | Some concerns | Low* |
|  | Adults | Some trials at risk | High statistical heterogeneity | Diverse but representative populations | No concerns | Some concerns | Low* |
| Postoperative shivering | Overall | Overall moderate risk of bias | No concerns | Diverse but representative populations | No concerns | No concerns | Moderate |
|  | Cardiac | One trial at high risk | No concerns | No concerns | Large RR 95%CI range 0.81 to 1.75 | No concerns | Very low |
|  | Non-cardiac | Some trials at risk | No concerns | Diverse but representative populations | No concerns | No concerns | Moderate |
| Accidental awakenings / awareness | Overall | Some trials at risk | No concerns | No concerns | Very large RR 95%CI range 0.41 to 5.68 | No concerns | Very low |
|  | Cardiac | NA | NA | NA | NA | NA | NA |
|  | Non-cardiac | Some trials at risk | No concerns | No concerns | Very large RR 95%CI range 0.41 to 5.68 | No concerns | Very low |
| QoR-40 / Quality of Recovery | Overall | Overall low risk of bias | High statistical heterogeneity | Diverse but representative populations | No concerns | Some trials at risk | Low |
|  | Cardiac | No concerns | No concerns | No concerns | Very large mean difference 95%CI | No concerns | Low |
|  | Non-cardiac | Overall low risk of bias | High statistical heterogeneity | Diverse but representative populations | No concerns | Some trials at risk | Low |
| First pain score <12 hours (0-10 score) | Overall | Some trials at risk | High statistical heterogeneity | Diverse but representative populations | No concerns | Some concerns | Very low |
|  | Cardiac | Some trials at risk | High statistical heterogeneity | No concerns | No concerns | Some concerns | Very low |
|  | Non-cardiac | Some trials at risk | High statistical heterogeneity | Diverse but representative populations | No concerns | Some trials at high risk | Very low |
| Pain score 12-24 hours (0-10 score) | Overall | Some trials at risk | High statistical heterogeneity | Diverse but representative populations | No concerns | Some concerns | Very low |
|  | Cardiac | Some trials at risk | No concerns | No concerns | No concerns | No concerns | Moderate |
|  | Non-cardiac | Some trials at risk | High statistical heterogeneity | Diverse but representative populations | No concerns | Some concerns | Very low |
| Pain score 24-48 hours (0-10 score) | Overall | Some trials at risk | High statistical heterogeneity | Diverse but representative populations | No concerns | Some concerns | Very low |
|  | Cardiac | Some trials at risk | No concerns | No concerns | No concerns | Some concerns | Low |
|  | Non-cardiac | Some trials at risk | High statistical heterogeneity | Diverse but representative populations | No concerns | Some trials at high risk | Very low |
| Clavien-Dindo grades ≥3 | Overall | Some trials at risk | No concerns | Diverse but representative populations | No concerns | No concerns | Moderate |
|  | Cardiac | NA | NA | NA | NA | NA | NA |
|  | Non-cardiac | Some trials at risk | No concerns | Diverse but representative populations | No concerns | No concerns | Moderate |
| Major bleeding | Overall | Some trials at risk | No concerns | Diverse but representative populations | No concerns | No concerns | Moderate |
|  | Cardiac | Some trials at risk | No concerns | No concerns | Large RR 95%CI range 0.88 to 2.00 | No concerns | Low |
|  | Non-cardiac | Some trials at risk | No concerns | Diverse but representative populations | No concerns | No concerns | Moderate |
| Surgical site infection | Overall | Some trials at risk | No concerns | Diverse but representative populations | No concerns | No concerns | Moderate |
|  | Cardiac | NA | NA | NA | NA | NA | NA |
|  | Non-cardiac | Some trials at risk | No concerns | Diverse but representative populations | No concerns | No concerns | Moderate |

**Appendix 6: Meta-analysis results of efficiency outcomes with ≥5 RCTs**

*Supplementary Table 4: Effect estimate results of the meta-analysis for cardiac and non-cardiac surgery for the efficiency outcome measures. Prespecified outcomes are reported that include at least five RCTs, all other outcomes are listed in eAppendix 8. Abbreviations: CI confidence interval, RR risk ratio, MD mean difference, SMD standardized mean difference, RE random effect, PACU post anaesthesia care unit.*

| Outcome measures | Population | No of RCTs | No of patients | Statistical model | Effect estimate (95% CI) | Preference | I^2^ | P-value |
| --- | --- | --- | --- | --- | --- | --- | --- | --- |
| Efficiency – Anaesthetic & General |  |  |  |  |  |  |  |  |
| Intraoperative opioid consumption | Cardiac  Non-cardiac | 8  72 | 1145  10,164 | SMD, RE  SMD, RE | 0.25 (0.11,0.38)  0.37 (0.18,0.56) | **Inhalation**  **Inhalation** | 21%  95% | **0.0004**  **0.0001** |
| Postoperative opioid consumption | Cardiac  Non-cardiac | 4  33 | 606  6008 | SMD, RE  SMD, RE | -0.05 (-0.71, 0.62)  0.04 (-0.14, 0.21) | None  None | 93%  84% | 0.89  0.68 |
| Time to extubation (minutes) | Cardiac  Non-cardiac | 12  101 | 1050  10,413 | MD, RE  MD, RE | 13.1 (-24.37,50.53)  0.44 (-0.16,1.03) | None  None | 89%  96% | 0.49  0.15 |
| Time to awakening (minutes) | Cardiac  Non-cardiac | 2  67 | 151  6602 | MD, RE  MD, RE | -0,67 (-1.90,0.56)  0.51 (-0.07,1.09) | None  None | 0%  97% | 0.28  0.09 |
| Time to follow simple instruction (minutes) | Cardiac  Non-cardiac | 0  42 | 0  4056 | MD, RE | Not estimable  0.78 (0.15,1.41) | **Inhalation** | 95% | **0.02** |
| Time to respiratory recovery (minutes) | Cardiac  Non-cardiac | 0  31 | 0  4360 | MD, RE | Not estimable  0.99 (0.04,1.95) | **Inhalation** | 98% | **0.04** |
| Time to reach a recovery score (minutes) | Cardiac  Non-cardiac | 0  28 | 0  3617 | MD, RE | Not estimable  0.38 (-1.72,2.47) | None | 98% | 0.72 |
| Time to orientation (minutes) | Cardiac  Non-cardiac | 0  26 | 0  2254 | MD, RE | Not estimable  2.00 (1.02,2.98) | **Inhalation** | 94% | **<0.0001** |
| PACU time (minutes) | Cardiac  Non-cardiac | 0  48 | 0  5627 | MD, RE | Not estimable  -1.59 (-3.15,-0.03) | **TIVA** | 88% | **0.05** |
| Length of hospital stay / LoS (days) | Cardiac  Non-cardiac | 29  46 | 10,223  8483 | MD, RE  MD, RE | 0.63 (-0.04,1.29)  0.04 (-0.14,0.23) | None  None | 96%  57% | 0.06  0.63 |
| Unplanned hospital (re)admissions | Cardiac  Non-cardiac | 4  3 | 5735  304 | RR, RE  RR, RE | 0.87 (0.54,1.43)  0.63 (0.28,1.42) | None  None | 21%  0% | 0.59  0.26 |
| Unplanned ICU admission | Cardiac  Non-cardiac | 2  3 | 300  1038 | RR, RE  RR, RE | 1.22 (0.23,6.40)  0.89 (0.65,1.23) | None  None | 0%  0% | 0.81  0.49 |

**Appendix 7: Meta-analysis results of outcomes including <5 RCTs**

*Supplementary Table 5: Effect estimate results of the meta-analysis for cardiac and non-cardiac surgery for the morbidity and efficiency outcomes that included less than five RCTs. Abbreviations: RR risk ratio, MD mean difference, RE random effect, NA not applicable.*

| Outcome measures | Population | No of RCTs | No of patients | Statistical model | Effect estimate (95% CI) | Preference | I^2^ | P-value |
| --- | --- | --- | --- | --- | --- | --- | --- | --- |
| Morbidity & Efficiency |  |  |  |  |  |  |  |  |
| Myocardial injury | Cardiac  Non-cardiac | 2  2 | 300  511 | RR, RE  RR, RE | 1.76 (1.27,2.44)  1.16 (0.76,1.77) | **Inhalation**  None | 39%  57% | **0.0007**  0.49 |
| Cardiovascular death | Cardiac  Non-cardiac | 1  2 | 5400  1580 | RR, RE  RR, RE | 1.73 (0.89,3.33)  1.20 (0.41,3.51) | None  None | NA  0% | 0.10  0.74 |
| Pulmonary embolism | Cardiac  Non-cardiac | 0  4 | 0  3106 | RR, RE | Not estimable  1.14 (0.27,4.87) | None | 0% | 0.86 |
| Deep vein thrombosis | Cardiac  Non-cardiac | 0  3 | 0  3099 | RR, RE | Not estimable  0.95 (0.44,2.05) | None | 0% | 0.89 |
| Major adverse cardiac events / MACE | Cardiac  Non-cardiac | 0  2 | 0  559 | RR, RE | Not estimable  1.02 (0.58,1.78) | None | 0% | 0.94 |
| Non-fatal cardiac event | Cardiac  Non-cardiac | 0  0 | 0  0 |  | Not estimable |  |  |  |
| Coronary revascularisation | Cardiac  Non-cardiac | 0  0 | 0  0 |  | Not estimable |  |  |  |
| ARDS | Cardiac  Non-cardiac | 2  2 | 86  80 | RR, RE  RR, RE | 4.50 (0.23,87.61)  3.00 (0.13,69.52) | None  None | NA  NA | 0.32  0.49 |
| Aspiration | Cardiac  Non-cardiac | 0  2 | 0  687 | RR, RE | Not estimable  Not estimable | None | NA | NA |
| Renal replacement therapy / RRT | Cardiac  Non-cardiac | 2  1 | 5500  59 | RR, RE  RR, RE | 0.88 (0.43,1.80)  0.16 (0.02,1.26) | None  None | NA  NA | 0.73  0.08 |
| QoR-15 / Quality of Recovery | Cardiac  Non-cardiac | 1  3 | 489  303 | RR, RE  RR, RE | -0.40 (-1.66,0.86)  6.26 (1.80,10.71) | None  **TIVA** | NA  0% | 0.53  **0.006** |
| Cancer recurrence | Cardiac  Non-cardiac | 0  3 | 0  2206 | RR, RE | 0.93 (0.73,1.19) | None | 0% | 0.58 |
| WHODAS 1.0 or 2.0 | Cardiac  Non-cardiac | 0  0 | 0  0 |  | Not estimable |  |  |  |
| Cost analysis | Cardiac  Non-cardiac | 0  4 | 0  480 | MD, RE | -1,51 (-8.83,5.81) | None | 96% | 0.69 |

**Appendix 8: List of 317 RCT included in the meta-analysis, including risk of bias**

*Supplementary Table 6: Included RCTs, characteristics and risk of bias.*

| Name | No patients | Inhalation anaesthetic | Population | Type of surgery | Overall risk of bias | Random sequence generation | Allocation concealment | Blinding of patients and personnel | Blinding of outcome assessment | Incomplete outcome data | Selective reporting |
| --- | --- | --- | --- | --- | --- | --- | --- | --- | --- | --- | --- |
| Abdeldayem 2021 | 100 | Sevoflurane | Children <18 | Non-cardiac | Moderate | Low | Low | Not reported | Not reported | Low | Low |
| Aditianingsih 2019 | 46 | Sevoflurane | Adults ≥18 | Non-cardiac | Moderate | Low | Low | Not reported | Not reported | Low | Low |
| Aftab 2019 | 183 | Desflurane | Adults ≥18 | Non-cardiac | Moderate | Low | Low | Low | Not reported | Not reported | Low |
| Ahmadzadeh Amiri 2020 | 105 | Isoflurane | Adults ≥18 | Non-cardiac | High | Low | Low | High | High | Low | Low |
| Akdogan 2022 | 60 | Sevoflurane | Adults ≥18 | Non-cardiac | Moderate | Low | Not reported | Not reported | Not reported | Not reported | Low |
| Akkurt 2009 | 60 | Desflurane | Adults ≥18 | Non-cardiac | High | Low | Not reported | Low | High | High | Low |
| Ammar 2016 | 50 | Sevoflurane | Adults ≥18 | Non-cardiac | Low | Low | Low | Low | Low | Low | Low |
| Apfel 2004 | 5161 | Unspecified | Adults ≥18 | Non-cardiac | Low | Low | Low | Low | Low | Low | Low |
| Aribawa 2023 | 60 | Sevoflurane | Adults ≥18 | Non-cardiac | Moderate | Low | Not reported | Not reported | Not reported | Low | Low |
| Ballester 2011 | 38 | Sevoflurane | Adults ≥18 | Cardiac | High | Low | High | Low | Not reported | Not reported | Low |
| Bassuoni 2012 | 126 | Sevoflurane | Adults ≥18 | Non-cardiac: vascular | High | Not reported | High | Not reported | Low | Low | Not reported |
| Beck-Schimmer 2015 | 98 | Sevoflurane | Adults ≥18 | Non-cardiac: pulmonary | Low | Low | Low | Low | Low | Low | Low |
| Beck-Schimmer 2016 | 460 | Desflurane | Adults ≥18 | Non-cardiac: pulmonary | Low | Low | Low | Low | Low | Low | Low |
| Bein 2005 | 50 | Sevoflurane | Adults ≥18 | Cardiac | Moderate | Not reported | Not reported | Low | Not reported | Low | Low |
| Bettex 2014 | 21 | Sevoflurane | Children <18 | Cardiac | Moderate | Low | Not reported | Low | Not reported | Low | Low |
| Bhagat 2021 | 91 | Desflurane | Adults ≥18 | Non-cardiac: intracranial | Low | Low | Low | Low | Low | Low | Low |
| Bhakta 2016 | 60 | Isoflurane | Adults ≥18 | Non-cardiac | High | Not reported | High | High | Low | Not reported | Low |
| Bharadwaj 2020 | 60 | Desflurane | Adults ≥18 | Non-cardiac: intracranial | Moderate | Low | Low | Not reported | Low | Not reported | Low |
| Bhardwaj 2018 | 70 | Desflurane | Adults ≥18 | Non-cardiac: intracranial | Moderate | Low | Low | Not reported | Not reported | Low | Low |
| Bhusari 2022 | 100 | Sevoflurane | Adults ≥18 | Non-cardiac | Moderate | Not reported | Not reported | Not reported | Not reported | Not reported | Low |
| Biboulet 2012 | 30 | Sevoflurane | Elderly | Non-cardiac | Moderate | Not reported | Not reported | Low | Not reported | Not reported | Low |
| Bignami 2011 | 100 | Sevoflurane | Adults ≥18 | Cardiac | Moderate | Not reported | Not reported | Low | Low | Low | Low |
| Bindra 2019 | 80 | Sevoflurane | Adults ≥18 | Non-cardiac | Low | Low | Low | Low | Low | Low | Low |
| Bischoff 1998 | 15 | Isoflurane | Adults ≥18 | Non-cardiac | Moderate | Not reported | Not reported | Not reported | Not reported | Not reported | Not reported |
| Blobner 1994 | 34 | Isoflurane | Adults ≥18 | Non-cardiac | High | Not reported | Not reported | Low | High | High | Low |
| Bocskai 2018 | 119 | Sevoflurane | Adults ≥18 | Non-cardiac | Moderate | Low | Low | Low | Low | Not reported | Low |
| Boisson-Bertrand 1990 | 43 | Isoflurane | Adults ≥18 | Non-cardiac | Moderate | Low | Not reported | Low | Not reported | Not reported | Low |
| Bostek 1992 | 43 | Isoflurane | Adults ≥18 | Non-cardiac | High | Not reported | Not reported | Low | High | High | Low |
| Braun 2005 | 40 | Desflurane | Adults ≥18 | Non-cardiac | High | Not reported | Not reported | Low | High | High | Low |
| Braz 2013 | 30 | Isoflurane | Adults ≥18 | Non-cardiac | High | Not reported | High | Low | Not reported | Not reported | Low |
| Cai 2012 | 2000 | Isoflurane | Elderly | Non-cardiac | Moderate | Low | Not reported | Low | Not reported | Not reported | Low |
| Cao 2023 | 1195 | Sevoflurane | Elderly | Non-cardiac | Low | Low | Low | Low | Low | Low | Low |
| Çaparlar 2017 | 79 | Sevoflurane | Adults ≥18 | Non-cardiac | High | Low | Not reported | High | High | Low | Low |
| Carles 2008 | 20 | Sevoflurane | Adults ≥18 | Non-cardiac | High | Low | Not reported | Low | High | High | Low |
| Carli 2020 | 121 | Sevoflurane | Adults ≥18 | Non-cardiac | High | Low | Low | High | Low | Low | Low |
| Caverni 2005 | 120 | Sevoflurane, desflurane | Adults ≥18 | Non-cardiac: intracranial | High | Not reported | Not reported | Not reported | High | High | Not reported |
| Çelik 2011 | 100 | Sevoflurane | Adults ≥18 | Non-cardiac | High | Not reported | Not reported | Low | High | High | Low |
| Chan 2009 | 75 | Sevoflurane, desflurane | Adults ≥18 | Non-cardiac | High | Not reported | High | Low | High | High | Low |
| Chang 2022 | 70 | Sevoflurane | Adults ≥18 | Non-cardiac | High | Low | Low | High | Not reported | Low | Low |
| Che 2022 | 40 | Sevoflurane | Adults ≥18 | Cardiac | Moderate | Low | Not reported | Not reported | Not reported | Not reported | Low |
| Chen 2013 | 84 | Sevoflurane | Elderly | Non-cardiac | Moderate | Low | Low | Low | Not reported | Low | Low |
| Chen 2018 | 200 | Sevoflurane | Adults ≥18 | Non-cardiac | Moderate | Low | Not reported | Not reported | Not reported | Not reported | Low |
| Chen C 2022 | 83 | Sevoflurane | Adults ≥18 | Non-cardiac | Moderate | Low | Not reported | Low | Low | Not reported | Low |
| Chen P 2022 | 47 | Sevoflurane | Adults ≥18 | Non-cardiac: vascular | Moderate | Low | Low | Low | Low | Not reported | Low |
| Chen Y 2022 | 90 | Sevoflurane | Adults ≥18 | Non-cardiac | Moderate | Low | Not reported | Not reported | Low | Low | Low |
| Cheng 2008 | 40 | Isoflurane | Adults ≥18 | Non-cardiac | High | Low | High | Low | Not reported | Low | Low |
| Cho 2017 | 48 | Sevoflurane | Adults ≥18 | Non-cardiac | High | Low | Low | High | Low | Low | Low |
| Choi 2022 | 48 | Sevoflurane | Adults ≥18 | Non-cardiac | Moderate | Low | Not reported | Low | Low | Low | Low |
| Chung 2000 | 569 | Isoflurane | Adults ≥18 | Non-cardiac | High | Low | High | Low | High | High | Low |
| Chung 2018 | 100 | Sevoflurane | Adults ≥18 | Non-cardiac | High | Low | High | Not reported | Not reported | Not reported | Low |
| Citerio 2012 | 274 | Sevoflurane | Adults ≥18 | Non-cardiac: intracranial | Moderate | Low | Not reported | Low | Not reported | Not reported | Low |
| Cok 2011 | 40 | Isoflurane | Adults ≥18 | Non-cardiac: intracranial | High | Low | Not reported | Low | High | High | Low |
| Conzen 2003 | 20 | Sevoflurane | Adults ≥18 | Cardiac | Moderate | Not reported | Not reported | Low | Not reported | Not reported | Low |
| Cotoia 2018 | 136 | Sevoflurane, desflurane | Adults ≥18 | Non-cardiac | Low | Low | Low | Low | Low | Low | Low |
| Cromheecke 2006 | 30 | Sevoflurane | Adults ≥18 | Cardiac | High | Low | High | Low | Not reported | Not reported | Low |
| Dabir 2015 | 88 | Isoflurane | Adults ≥18 | Non-cardiac | High | Low | High | Low | High | Not reported | Low |
| Dai M 2021 | 116 | Sevoflurane | Adults ≥18 | Non-cardiac | Moderate | Low | Not reported | Not reported | Not reported | Low | Low |
| Dai Z 2021 | 164 | Sevoflurane | Adults ≥18 | Non-cardiac | Low | Low | Low | Low | Low | Low | Low |
| De Conno 2009 | 54 | Sevoflurane | Adults ≥18 | Non-cardiac: pulmonary | High | Not reported | High | Low | Low | Low | Low |
| De Hert 2004 | 240 | Sevoflurane, desflurane | Adults ≥18 | Cardiac | High | Not reported | High | Low | Not reported | Not reported | Low |
| de la Gala 2017 | 174 | Sevoflurane | Adults ≥18 | Non-cardiac: pulmonary | Low | Low | Low | Low | Low | Low | Low |
| De Oliveira 2017 | 75 | Sevoflurane | Adults ≥18 | Non-cardiac | Moderate | Low | Low | Not reported | Low | Low | Low |
| Demirel 2021 | 120 | Desflurane | Adults ≥18 | Non-cardiac | Moderate | Not reported | Not reported | Low | Low | Low | Low |
| Ding DF 2021 | 180 | Sevoflurane | Adults ≥18 | Non-cardiac | Moderate | Not reported | Not reported | Not reported | Not reported | Not reported | Low |
| Ding F 2021 | 130 | Sevoflurane | Elderly | Non-cardiac | Moderate | Low | Not reported | Not reported | Not reported | Low | Low |
| Duan 2023 | 289 | Sevoflurane | Adults ≥18 | Cardiac | Low | Low | Low | Low | Low | Low | Low |
| Dubowitz 2021 | 146 | Sevoflurane | Adults ≥18 | Non-cardiac | Low | Low | Low | Low | Low | Low | Low |
| Dutta 2023 | 33 | Desflurane | Adults ≥18 | Non-cardiac | Moderate | Low | Low | Not reported | Low | Low | Low |
| Ebert 2000 | 52 | Sevoflurane, desflurane | Adults ≥18 | Non-cardiac | Moderate | Not reported | Not reported | Low | Not reported | Not reported | Low |
| Elbakry 2018 | 100 | Desflurane | Adults ≥18 | Non-cardiac | Moderate | Low | Not reported | Low | Low | Low | Low |
| Enlund 2023 | 1670 | Sevoflurane | Adults ≥18 | Non-cardiac | Low | Low | Low | Low | Low | Low | Low |
| Eroglu 2003 | 40 | Sevoflurane | Children <18 | Non-cardiac: intracranial | High | Not reported | High | Not reported | Not reported | Not reported | Not reported |
| Estephan 2023 | 111 | Sevoflurane | Adults ≥18 | Non-cardiac | High | High | Not reported | High | High | Low | Low |
| Ewaldsson 2005 | 29 | Isoflurane | Adults ≥18 | Non-cardiac | High | Not reported | High | Low | Not reported | Not reported | Low |
| Fabregas 1995 | 58 | Isoflurane | Adults ≥18 | Non-cardiac: intracranial | High | Not reported | Not reported | Low | High | High | Low |
| Fang 2021 | 51 | Sevoflurane | Adults ≥18 | Cardiac | Moderate | Low | Low | Not reported | Not reported | Not reported | Low |
| Flier 2010 | 84 | Isoflurane | Adults ≥18 | Cardiac | High | Low | Not reported | Low | High | Low | Low |
| Fung 2008 | 20 | Sevoflurane | Children <18 | Non-cardiac | High | Low | High | Low | High | High | Low |
| Geng 2017 | 150 | Sevoflurane, isoflurane | Elderly | Non-cardiac | Low | Low | Low | Low | Low | Low | Low |
| Ghanem 2021 | 50 | Sevoflurane | Adults ≥18 | Non-cardiac: intracranial | Moderate | Low | Low | Not reported | Not reported | Low | Low |
| Goel 2020 | 100 | Sevoflurane | Adults ≥18 | Non-cardiac | Moderate | Low | Not reported | Not reported | Not reported | Low | Low |
| Goerlich 2000 | 180 | Sevoflurane | Children <18 | Non-cardiac | Moderate | Not reported | Not reported | Low | Not reported | Not reported | Low |
| Gokce 2007 | 40 | Desflurane | Adults ≥18 | Non-cardiac | High | Low | High | Low | High | High | Low |
| Gollapudy 2020 | 47 | Desflurane | Adults ≥18 | Non-cardiac | Moderate | Not reported | Low | Low | Low | Low | Low |
| Gouda 2003 | 40 | Sevoflurane | Children <18 | Non-cardiac | High | Not reported | Not reported | Low | High | High | Low |
| Goyal 2021 | 50 | Sevoflurane | Children <18 | Non-cardiac | Low | Low | Low | Low | Low | Low | Low |
| Gravel 1999 | 30 | Sevoflurane | Adults ≥18 | Cardiac | High | Not reported | High | Low | High | High | Low |
| Grottke 2004 | 36 | Desflurane | Adults ≥18 | Non-cardiac: intracranial | High | Not reported | Not reported | Low | High | Not reported | Low |
| Grundmann 2001 | 50 | Desflurane | Adults ≥18 | Non-cardiac | High | Not reported | Not reported | Low | High | High | Low |
| Guarracino 2006 | 112 | Desflurane | Adults ≥18 | Cardiac | Low | Low | Low | Low | Low | Low | Low |
| Guinot 2020 | 81 | Sevoflurane | Adults ≥18 | Cardiac | High | Low | Not reported | High | Low | Not reported | Low |
| Güler 2014 | 40 | Sevoflurane | Adults ≥18 | Non-cardiac | Moderate | Low | Not reported | Low | Not reported | Not reported | Low |
| Guo 2020 | 234 | Sevoflurane | Elderly | Non-cardiac | Low | Low | Low | Low | Low | Low | Low |
| Haldar 2020 | 90 | Sevoflurane, desflurane | Adults ≥18 | Non-cardiac: intracranial | Moderate | Low | Low | Not reported | Low | Low | Low |
| Han 2020 | 80 | Sevoflurane | Adults ≥18 | Non-cardiac | High | High | Low | Low | Low | Low | Low |
| Hans 2008 | 34 | Sevoflurane | Adults ≥18 | Non-cardiac: intracranial | High | Low | High | Low | Low | Low | Low |
| He 2022 | 514 | Unspecified | Adults ≥18 | Cardiac | Low | Low | Low | Low | Low | Low | Low |
| Hernández-Palazón 2006 | 90 | Sevoflurane | Adults ≥18 | Non-cardiac: intracranial | High | Not reported | Not reported | Low | High | High | Low |
| Höcker 2006 | 103 | Sevoflurane | Adults ≥18 | Non-cardiac | High | Low | High | Low | High | High | Low |
| Hofer C2003 | 301 | Sevoflurane | Adults ≥18 | Non-cardiac | High | Low | Not reported | Low | High | High | Low |
| Hofland 2017 | 299 | Sevoflurane | Adults ≥18 | Cardiac | Low | Low | Low | Low | Low | Low | Low |
| Honca 2017 | 60 | Desflurane | Adults ≥18 | Non-cardiac | High | Not reported | Low | High | High | Low | Low |
| Horng 2008 | 90 | Sevoflurane, desflurane | Adults ≥18 | Non-cardiac | High | Low | High | Low | High | High | Low |
| Hou 2017 | 90 | Sevoflurane | Adults ≥18 | Cardiac | Moderate | Not reported | Not reported | Low | Low | Low | Low |
| Huang 2011 | 30 | Isoflurane | Adults ≥18 | Cardiac | High | Low | High | Low | Not reported | Low | Low |
| Huang 2017 | 64 | Isoflurane | Adults ≥18 | Non-cardiac | Moderate | Not reported | Not reported | Not reported | Not reported | Low | Low |
| Ishii 2016 | 59 | Sevoflurane | Elderly | Non-cardiac | Moderate | Not reported | Not reported | Low | Not reported | Not reported | Low |
| Ivani 1996 | 24 | Isoflurane | Children <18 | Non-cardiac | Moderate | Not reported | Not reported | Low | Not reported | Not reported | Low |
| Jain 2018 | 80 | Desflurane | Children <18 | Non-cardiac | Moderate | Not reported | Not reported | Not reported | Not reported | Low | Low |
| Jellish 1995 | 68 | Isoflurane | Adults ≥18 | Non-cardiac | High | Not reported | Not reported | Low | High | High | Low |
| Jellish 1999 | 79 | Isoflurane | Adults ≥18 | Non-cardiac | High | Not reported | Not reported | Low | High | High | Low |
| Jeong 2012 | 24 | Sevoflurane | Elderly | Non-cardiac | Moderate | Low | Not reported | Not reported | Low | Low | Low |
| Ji 2018 | 60 | Sevoflurane | Adults ≥18 | Non-cardiac | Moderate | Low | Not reported | Low | Low | Low | Low |
| Jia 2015 | 67 | Sevoflurane | Adults ≥18 | Cardiac | High | Not reported | Not reported | High | High | High | High |
| Jiang 2016 | 100 | Sevoflurane | Adults ≥18 | Non-cardiac | Moderate | Not reported | Not reported | Low | Not reported | Not reported | Low |
| Jiang J-L 2023 | 669 | Unspecified | Adults ≥18 | Cardiac | Low | Low | Low | Low | Low | Low | Low |
| Jiang Z 2023 | 103 | Sevoflurane | Adults ≥18 | Non-cardiac: intracranial | Low | Low | Low | Low | Low | Low | Low |
| Jo 2019 | 80 | Sevoflurane | Adults ≥18 | Non-cardiac | Moderate | Low | Not reported | Low | Low | Low | Low |
| Jo 2021 | 72 | Desflurane | Adults ≥18 | Non-cardiac | Moderate | Low | Not reported | Low | Low | Low | Low |
| Joe 2021 | 116 | Desflurane | Adults ≥18 | Non-cardiac | Moderate | Low | Not reported | Low | Low | Low | Low |
| Jokela 2000 | 120 | Sevoflurane | Adults ≥18 | Non-cardiac | High | Not reported | Not reported | Low | High | High | Low |
| Kalimeris 2013 | 44 | Sevoflurane | Adults ≥18 | Non-cardiac: vascular | Moderate | Not reported | Not reported | Low | Not reported | Not reported | Low |
| Kang 2023 | 95 | Sevoflurane | Adults ≥18 | Cardiac | Moderate | Low | Not reported | Low | Low | Low | Low |
| Kapil 2018 | 75 | Sevoflurane, desflurane | Adults ≥18 | Non-cardiac: intracranial | Moderate | Low | Low | Not reported | Not reported | Low | Low |
| Karam 2023 | 134 | Sevoflurane | Children <18 | Non-cardiac | Low | Low | Low | Low | Low | Low | Low |
| Kawagoe 2022 | 80 | Desflurane | Adults ≥18 | Non-cardiac: pulmonary | Moderate | Low | Low | Not reported | Not reported | Low | Low |
| Kawanishi 2022 | 50 | Desflurane | Adults ≥18 | Non-cardiac: pulmonary | Low | Low | Low | Low | Low | Low | Low |
| Kawano 2016 | 24 | Sevoflurane | Adults ≥18 | Cardiac | Moderate | Low | Not reported | Low | Not reported | Not reported | Low |
| Keller 2005 | 60 | Sevoflurane, isoflurane | Adults ≥18 | Non-cardiac | High | Not reported | High | Low | High | Not reported | Low |
| Khakzad 2019 | 60 | Isoflurane | Adults ≥18 | Non-cardiac | Moderate | Not reported | Not reported | Not reported | Not reported | Low | Low |
| Khanjani 2014 | 90 | Isoflurane | Adults ≥18 | Non-cardiac | Moderate | Low | Not reported | Low | Low | Low | Low |
| Kim 2011 | 94 | Sevoflurane | Adults ≥18 | Cardiac | High | Low | Not reported | Low | High | Low | Low |
| Kim 2012 | 70 | Sevoflurane | Adults ≥18 | Non-cardiac | Moderate | Low | Not reported | Low | Not reported | Not reported | Low |
| Kim 2015 | 100 | Desflurane | Adults ≥18 | Non-cardiac | Moderate | Low | Not reported | Low | Low | Low | Low |
| Kim 2017 | 82 | Sevoflurane | Adults ≥18 | Non-cardiac | Low | Low | Low | Low | Low | Low | Low |
| Kim 2020 | 198 | Sevoflurane | Adults ≥18 | Non-cardiac | Low | Low | Low | Low | Low | Low | Low |
| Kim 2021 | 68 | Desflurane | Adults ≥18 | Non-cardiac | Moderate | Low | Not reported | Low | Low | Low | Low |
| Kim DH 2022 | 101 | Sevoflurane | Adults ≥18 | Non-cardiac: intracranial | Moderate | Low | Not reported | Low | Low | Low | Low |
| Kim JE 2022 | 42 | Sevoflurane | Adults ≥18 | Non-cardiac | Moderate | Not reported | Low | Not reported | Not reported | Low | Low |
| Kim SH 2022 | 120 | Desflurane | Adults ≥18 | Non-cardiac | Moderate | Low | Not reported | Low | Low | Low | Low |
| Kletecka 2018 | 43 | Sevoflurane | Adults ≥18 | Non-cardiac | Moderate | Not reported | Low | Low | Low | Low | Low |
| Ko 2008 | 70 | Desflurane | Adults ≥18 | Non-cardiac | Moderate | Low | Not reported | Not reported | Not reported | Low | Not reported |
| Kocaturk 2018 | 116 | Sevoflurane | Children <18 | Non-cardiac | Moderate | Not reported | Low | Low | Low | Low | Low |
| Kochs 2000 | 553 | Isoflurane | Adults ≥18 | Non-cardiac | High | Low | High | Low | High | High | Low |
| Kotani 1998 | 60 | Isoflurane | Adults ≥18 | Non-cardiac | Moderate | Not reported | Not reported | Low | Not reported | Not reported | Low |
| Kowark 2018 | 343 | Sevoflurane, desflurane | Adults ≥18 | Non-cardiac | Low | Low | Low | Low | Low | Low | Low |
| Krueper 1997 | 60 | Isoflurane | Adults ≥18 | Non-cardiac | Moderate | Not reported | Not reported | Low | Not reported | Not reported | Low |
| Kumar 2016 | 60 | Sevoflurane | Adults ≥18 | Non-cardiac: intracranial | Moderate | Low | Not reported | Low | Not reported | Not reported | Low |
| Kuzkov 2018 | 40 | Sevoflurane | Adults ≥18 | Non-cardiac: vascular | Moderate | Not reported | Not reported | Not reported | Low | Not reported | Low |
| Lan 2021 | 126 | Sevoflurane | Elderly | Non-cardiac | Moderate | Low | Not reported | Not reported | Not reported | Not reported | Low |
| Landoni 2014 | 200 | Sevoflurane | Adults ≥18 | Cardiac | Low | Low | Low | Low | Low | Low | Low |
| Landoni 2019 | 5400 | Unspecified | Adults ≥18 | Cardiac | Low | Low | Low | Low | Low | Low | Low |
| Launo 1994 | 100 | Isoflurane | Adults ≥18 | Non-cardiac | Moderate | Not reported | Not reported | Not reported | Not reported | Not reported | Not reported |
| Lauta 2010 | 302 | Sevoflurane | Adults ≥18 | Non-cardiac: intracranial | High | Low | Not reported | Low | High | Not reported | Low |
| Law-Koune 2006 | 18 | Sevoflurane | Adults ≥18 | Cardiac | High | Low | High | Low | Not reported | Low | Low |
| Ledderose 1988 | 50 | Isoflurane | Adults ≥18 | Non-cardiac | Moderate | Not reported | Not reported | Low | Not reported | Not reported | Low |
| Ledowski 2005 | 43 | Sevoflurane | Adults ≥18 | Non-cardiac | High | Not reported | High | Low | High | Not reported | Low |
| Lee 2011 | 62 | Sevoflurane | Adults ≥18 | Non-cardiac | Moderate | Low | Not reported | Low | Not reported | Not reported | Low |
| Lee 2014 | 76 | Sevoflurane, desflurane | Adults ≥18 | Non-cardiac | Moderate | Low | Not reported | Low | Not reported | Not reported | Low |
| Lee SH 2015 | 416 | Sevoflurane | Adults ≥18 | Non-cardiac | Moderate | Low | Not reported | Low | Low | Low | Low |
| Lee WK 2015 | 76 | Desflurane | Adults ≥18 | Non-cardiac | Low | Low | Low | Low | Low | Low | Low |
| Leslie 2009 | 300 | Desflurane | Adults ≥18 | Non-cardiac | Moderate | Low | Not reported | Low | Not reported | Not reported | Low |
| Li 2012 | 60 | Sevoflurane | Adults ≥18 | Non-cardiac | Moderate | Not reported | Low | Low | Not reported | Not reported | Low |
| Li 2019 | 120 | Sevoflurane | Children <18 | Non-cardiac | Low | Low | Low | Low | Low | Low | Low |
| Li 2023 | 110 | Sevoflurane | Adults ≥18 | Non-cardiac | Low | Low | Low | Low | Low | Low | Low |
| Li S 2021 | 68 | Sevoflurane | Adults ≥18 | Non-cardiac | Moderate | Low | Low | Not reported | Not reported | Low | Low |
| Li XF 2021 | 513 | Sevoflurane, desflurane | Adults ≥18 | Non-cardiac: pulmonary | Low | Low | Low | Low | Low | Low | Low |
| Li Y 2021 | 447 | Sevoflurane | Elderly | Non-cardiac | Low | Low | Low | Low | Low | Low | Low |
| Liang 2022 | 140 | Sevoflurane | Elderly | Non-cardiac | Moderate | Not reported | Not reported | Low | Low | Low | Low |
| Likhvantsev 2016 | 868 | Sevoflurane | Adults ≥18 | Cardiac | Moderate | Low | Not reported | Not reported | Not reported | Low | Low |
| Lin 2019 | 60 | Desflurane | Adults ≥18 | Non-cardiac | Moderate | Not reported | Low | Not reported | Low | Not reported | Low |
| Lindqvist 2014 | 59 | Desflurane | Adults ≥18 | Non-cardiac | High | Low | High | Low | Not reported | Not reported | Low |
| Little 2018 | 30 | Desflurane | Adults ≥18 | Non-cardiac | Moderate | Low | Low | Low | Low | Not reported | Low |
| Liu 2014 | 62 | Isoflurane | Adults ≥18 | Non-cardiac | Moderate | Not reported | Not reported | Not reported | Not reported | Not reported | Low |
| Liu 2017 | 112 | Sevoflurane | Elderly | Non-cardiac | Moderate | Not reported | Not reported | Not reported | Not reported | Not reported | Low |
| Liu 2019 | 80 | Desflurane | Adults ≥18 | Non-cardiac | Moderate | Low | Low | Low | Low | Not reported | Low |
| Longás Valién 2004 | 90 | Sevoflurane | Adults ≥18 | Non-cardiac: vascular | High | Not reported | Not reported | Low | High | High | Low |
| Loop 2002 | 90 | Sevoflurane, desflurane | Adults ≥18 | Non-cardiac | High | Not reported | High | Low | High | High | Low |
| Lopez Alvarez 2001 | 70 | Desflurane | Adults ≥18 | Non-cardiac | High | Not reported | Not reported | Low | High | High | Low |
| Lorsomradee 2006 | 320 | Sevoflurane | Adults ≥18 | Cardiac | High | Low | High | Low | Not reported | Low | Low |
| Luginbuhl 2003 | 160 | Desflurane | Adults ≥18 | Non-cardiac | High | Not reported | High | Low | High | Not reported | Low |
| Luntz 2004 | 64 | Sevoflurane | Elderly | Non-cardiac | High | Low | High | Low | High | High | Low |
| Lurati Buse 2012 | 385 | Sevoflurane | Elderly | Non-cardiac | Moderate | Low | Low | Low | Not reported | Low | Low |
| Mahli 2011 | 40 | Desflurane | Adults ≥18 | Non-cardiac | High | Low | Not reported | Low | High | High | Low |
| Mahmoud 2011 | 50 | Isoflurane | Adults ≥18 | Non-cardiac: pulmonary | Moderate | Low | Not reported | Low | Low | Low | Low |
| Malagon 2005 | 60 | Sevoflurane | Children <18 | Cardiac | Moderate | Low | Not reported | Not reported | Not reported | Low | Low |
| Margarit 2014 | 60 | Isoflurane | Adults ≥18 | Non-cardiac | High | Low | Low | Low | High | Not reported | Low |
| Markovic Bozic 2016 | 40 | Sevoflurane | Adults ≥18 | Non-cardiac: intracranial | Low | Low | Low | Low | Low | Low | Low |
| Martikainen 1998 | 118 | Desflurane, isoflurane | Adults ≥18 | Non-cardiac | High | Not reported | Not reported | Low | High | High | Low |
| Martin-Castro 2008 | 120 | Sevoflurane | Adults ≥18 | Non-cardiac | High | Low | High | Low | Low | Low | Low |
| Mazoti 2013 | 34 | Isoflurane | Adults ≥18 | Non-cardiac | High | Not reported | High | Low | Not reported | Not reported | Low |
| Mei 2014 | 148 | Sevoflurane | Adults ≥18 | Non-cardiac | High | Low | Low | Low | High | Not reported | Low |
| Mei 2020 | 209 | Sevoflurane | Elderly | Non-cardiac | Moderate | Low | Low | Low | Low | Not reported | Low |
| Meng 2021 | 80 | Sevoflurane | Adults ≥18 | Non-cardiac | High | Low | Low | Low | Low | Not reported | High |
| Mohaghegh 2017 | 104 | Isoflurane | Adults ≥18 | Non-cardiac | Moderate | Low | Low | Low | Low | Not reported | Low |
| Monedero 1994 | 42 | Isoflurane | Adults ≥18 | Non-cardiac | High | Not reported | Not reported | Low | High | High | Low |
| Monisha 2023 | 60 | Isoflurane | Adults ≥18 | Non-cardiac | Moderate | Low | Not reported | Not reported | Not reported | Low | Low |
| Moro 2016 | 110 | Sevoflurane | Adults ≥18 | Non-cardiac | High | Low | Low | High | Low | Not reported | Low |
| Mousa 2013 | 40 | Sevoflurane | Adults ≥18 | Non-cardiac | High | Low | High | Low | High | High | Low |
| Mrozinski 2014 | 60 | Sevoflurane | Adults ≥18 | Cardiac | High | Not reported | High | Low | Not reported | Not reported | Low |
| Muralidhar 2008 | 40 | Isoflurane | Adults ≥18 | Cardiac | High | Not reported | High | Low | High | High | Low |
| Mutch 1995 | 27 | Isoflurane | Adults ≥18 | Non-cardiac: vascular | High | Not reported | Not reported | Low | High | High | Low |
| Na 2018 | 83 | Desflurane | Adults ≥18 | Non-cardiac | Low | Low | Low | Low | Low | Low | Low |
| Nakada 2010 | 210 | Sevoflurane, isoflurane | Adults ≥18 | Non-cardiac | Moderate | Not reported | Not reported | Not reported | Not reported | Not reported | Not reported |
| Nieuwenhuijs-Moeke 2017 | 37 | Sevoflurane | Adults ≥18 | Non-cardiac | Moderate | Low | Low | Low | Not reported | Low | Low |
| Niu 2021 | 102 | Sevoflurane | Adults ≥18 | Non-cardiac | Low | Low | Low | Low | Low | Low | Low |
| Nostdahl 2017 | 113 | Desflurane | Adults ≥18 | Non-cardiac | Moderate | Not reported | Low | Low | Not reported | Not reported | Low |
| Oddby-Muhrbeck 1994 | 90 | Isoflurane | Adults ≥18 | Non-cardiac | High | Not reported | High | Low | Not reported | Not reported | Low |
| O'Gara 2022 | 40 | Sevoflurane | Adults ≥18 | Cardiac | Moderate | Low | Low | Low | Not reported | Not reported | Low |
| Ogurlu 2014 | 80 | Sevoflurane | Adults ≥18 | Non-cardiac | Moderate | Not reported | Not reported | Low | Not reported | Not reported | Low |
| Oh 2018 | 201 | Sevoflurane | Adults ≥18 | Non-cardiac | Low | Low | Low | Low | Low | Low | Low |
| Ohtani 2008 | 60 | Sevoflurane | Adults ≥18 | Non-cardiac | High | Low | Not reported | Not reported | High | High | Not reported |
| Oikkonen 1994 | 30 | Isoflurane | Adults ≥18 | Non-cardiac | High | Not reported | Not reported | Low | High | High | Low |
| Omara 2019 | 80 | Sevoflurane | Children <18 | Non-cardiac | High | Low | Not reported | High | Low | Low | Low |
| Orak 2021 | 60 | Sevoflurane | Adults ≥18 | Non-cardiac | Moderate | Not reported | Not reported | Not reported | Not reported | Not reported | Low |
| Oriby 2021 | 84 | Sevoflurane | Children <18 | Non-cardiac | Moderate | Low | Not reported | Not reported | Not reported | Not reported | Low |
| Ortiz 2014 | 74 | Sevoflurane, desflurane, isoflurane | Adults ≥18 | Non-cardiac | High | Low | Low | Low | High | Not reported | Low |
| Ozdemır 2013 | 42 | Sevoflurane | Adults ≥18 | Non-cardiac | High | Low | High | Low | High | High | Low |
| Pan 2021 | 60 | Sevoflurane | Adults ≥18 | Non-cardiac | Moderate | not reported | Not reported | Not reported | Not reported | Not reported | Low |
| Pan 2023 | 102 | Sevoflurane | Children <18 | Non-cardiac | High | Not reported | Not reported | High | High | Low | Not reported |
| Panditrao 2013 | 40 | Sevoflurane | Adults ≥18 | Non-cardiac | Moderate | Low | Not reported | Low | Not reported | Not reported | Low |
| Park 2020 | 80 | Desflurane | Adults ≥18 | Non-cardiac | Low | Low | Low | Low | Low | Low | Low |
| Parker 2004 | 354 | Sevoflurane, isoflurane | Adults ≥18 | Cardiac | High | Low | Not reported | Low | High | Not reported | Low |
| Pieters 2010 | 180 | Sevoflurane | Adults ≥18 | Non-cardiac | High | Low | Low | Low | High | Not reported | Low |
| Poolsuppasit 2018 | 144 | Sevoflurane | Children <18 | Non-cardiac | Low | Low | Low | Low | Low | Low | Low |
| Potocnik 2015 | 36 | Sevoflurane | Adults ≥18 | Non-cardiac: pulmonary | Moderate | Low | Not reported | Low | Low | Low | Low |
| Prasanna 2010 | 60 | Isoflurane | Adults ≥18 | Non-cardiac | Moderate | Not reported | Not reported | Low | Not reported | Not reported | Low |
| Qiao 2015 | 60 | Sevoflurane | Elderly | Non-cardiac | Moderate | Not reported | Not reported | Low | Not reported | Low | Low |
| Qiao 2023 | 63 | Desflurane | Adults ≥18 | Non-cardiac | High | High | High | Low | High | Low | Low |
| Quintao 2023 | 155 | Sevoflurane | Adults ≥18 | Non-cardiac | Low | Low | Low | Low | Low | Low | Low |
| Robert 2021 | 52 | Desflurane | Adults ≥18 | Non-cardiac | Low | Low | Low | Low | Low | Low | Low |
| Rocha 2017 | 38 | Desflurane | Adults ≥18 | Non-cardiac | High | Low | Low | High | Low | Not reported | Low |
| Rohan 2005 | 30 | Sevoflurane | Elderly | Non-cardiac | Moderate | Low | Not reported | Low | Not reported | Low | Low |
| Rowbotham 1998 | 233 | Isoflurane | Adults ≥18 | Non-cardiac | High | Low | High | Low | High | High | Low |
| Sahoo 2019 | 60 | Sevoflurane, desflurane | Children <18 | Non-cardiac | Moderate | Low | Low | Not reported | Not reported | Low | Low |
| Salihoğlu 2001 | 40 | Sevoflurane | Adults ≥18 | Non-cardiac | Moderate | Not reported | Not reported | Low | Not reported | Not reported | Low |
| Salihoğlu 2004 | 40 | Sevoflurane | Adults ≥18 | Non-cardiac | Moderate | Not reported | Not reported | Low | Not reported | Not reported | Low |
| Schmidt 2001 | 120 | Sevoflurane | Children <18 | Non-cardiac | High | Not reported | Not reported | Low | High | High | Low |
| Schneider 2003 | 80 | Isoflurane | Adults ≥18 | Non-cardiac | High | Not reported | Not reported | Low | High | High | Low |
| Schricker 2001 | 12 | Desflurane | Adults ≥18 | Non-cardiac | Moderate | Not reported | Not reported | Low | Not reported | Not reported | Low |
| Sessler 2019 | 2108 | Sevoflurane | Adults ≥18 | Non-cardiac | Low | Low | Low | Low | Low | Low | Low |
| Seyfi 2022 | 80 | Isoflurane | Adults ≥18 | Non-cardiac | Moderate | Not reported | Not reported | Not reported | Not reported | Not reported | Low |
| Shah 2018 | 50 | Sevoflurane | Adults ≥18 | Non-cardiac | Moderate | Low | Not reported | Not reported | Not reported | Not reported | Low |
| Sharma 2020 | 49 | Desflurane | Adults ≥18 | Non-cardiac: intracranial | Moderate | Low | Not reported | Low | Not reported | Low | Low |
| Sheikhzade 2021 | 80 | Sevoflurane | Children <18 | Non-cardiac | Moderate | Low | Not reported | Not reported | Not reported | Low | Low |
| Shen 2014 | 180 | Sevoflurane, desflurane | Adults ≥18 | Non-cardiac | Moderate | Low | Not reported | Low | Not reported | Not reported | Low |
| Shi 2019 | 94 | Sevoflurane | Adults ≥18 | Cardiac | Moderate | Low | Not reported | Not reported | Not reported | Not reported | Low |
| Shin 2010 | 98 | Sevoflurane | Adults ≥18 | Non-cardiac | Moderate | Low | Low | Low | Not reported | Low | Low |
| Shin 2020 | 118 | Desflurane | Elderly | Non-cardiac | Moderate | Low | Low | Not reported | Low | Low | Low |
| Shinn 2011 | 35 | Sevoflurane | Adults ≥18 | Non-cardiac | High | High | High | Low | High | High | Low |
| Singh 2020 | 70 | Sevoflurane | Adults ≥18 | Non-cardiac | Moderate | Low | Not reported | Not reported | Not reported | Low | Low |
| Sirvinskas 2015 | 72 | Sevoflurane | Adults ≥18 | Cardiac | Moderate | Low | Low | Low | Not reported | Not reported | Low |
| Sneyd 2005 | 50 | Sevoflurane | Adults ≥18 | Non-cardiac: intracranial | High | Low | Low | Low | High | Not reported | Low |
| Song 2010 | 100 | Sevoflurane | Adults ≥18 | Non-cardiac | Moderate | Low | Not reported | Low | Not reported | Not reported | Low |
| Song 2013 | 102 | Sevoflurane | Adults ≥18 | Non-cardiac | High | Low | High | Low | Not reported | Not reported | Low |
| Song 2022 | 59 | Sevoflurane | Adults ≥18 | Non-cardiac: pulmonary | Low | Low | Low | Low | Low | Low | Low |
| Sorbara 1995 | 30 | Isoflurane | Adults ≥18 | Cardiac | High | Not reported | Not reported | Low | High | High | Low |
| Spaniolas 2019 | 83 | Unspecified | Adults ≥18 | Non-cardiac | Moderate | Low | Not reported | Not reported | Not reported | Not reported | Low |
| Speicher 1995 | 63 | Isoflurane | Adults ≥18 | Non-cardiac | High | Not reported | Not reported | Low | High | High | Low |
| Stevanovic 2008 | 60 | Sevoflurane | Adults ≥18 | Non-cardiac | High | Low | High | Not reported | High | High | Not reported |
| Struys 2002 | 40 | Sevoflurane | Adults ≥18 | Non-cardiac | High | Not reported | Not reported | Low | High | High | Low |
| Su 2020 | 120 | Sevoflurane | Adults ≥18 | Non-cardiac | Moderate | Not reported | Not reported | Not reported | Not reported | Not reported | Low |
| Sultan 2022 | 60 | Sevoflurane | Adults ≥18 | Non-cardiac | Moderate | Low | Not reported | Not reported | Not reported | Not reported | Low |
| Sung 2020 | 34 | Sevoflurane | Adults ≥18 | Cardiac | Moderate | Low | Low | Low | Not reported | Low | Low |
| Surya Lavanya 2022 | 40 | Isoflurane | Adults ≥18 | Non-cardiac | Moderate | Low | Not reported | Not reported | Not reported | Not reported | Low |
| Suryaprakash 2013 | 84 | Sevoflurane | Adults ≥18 | Cardiac | Moderate | Low | Not reported | Low | Low | Low | Low |
| Takzare 2018 | 40 | Sevoflurane | Children <18 | Cardiac | Moderate | Not reported | Not reported | Not reported | Low | Not reported | Low |
| Talih 2020 | 90 | Sevoflurane | Adults ≥18 | Non-cardiac | Moderate | Low | Not reported | Not reported | Not reported | Not reported | Low |
| Tan 2010 | 80 | Sevoflurane | Adults ≥18 | Non-cardiac | High | Low | Low | Low | High | Not reported | Low |
| Tanaka 2017 | 90 | Desflurane | Elderly | Non-cardiac | Moderate | Low | Not reported | Low | Low | Low | Low |
| Tang 2014 | 200 | Sevoflurane | Elderly | Non-cardiac | Low | Low | Low | Low | Low | Low | Low |
| Tang 2017 | 120 | Sevoflurane | Adults ≥18 | Non-cardiac: vascular | Moderate | Low | Not reported | Not reported | Not reported | Low | Low |
| Tang 2019 | 110 | Sevoflurane | Adults ≥18 | Cardiac | Moderate | Not reported | Not reported | Not reported | Not reported | Not reported | Low |
| Tantri 2021 | 40 | Sevoflurane | Adults ≥18 | Non-cardiac | Moderate | Low | Low | Not reported | Not reported | Low | Low |
| Tian 2017 | 62 | Sevoflurane | Adults ≥18 | Non-cardiac: pulmonary | Moderate | Not reported | Not reported | Not reported | Not reported | Not reported | Low |
| Tilvawala 2017 | 50 | Sevoflurane | Adults ≥18 | Non-cardiac | Moderate | Not reported | Not reported | Not reported | Not reported | Not reported | Low |
| Tramèr 1998 | 77 | Isoflurane | Children <18 | Non-cardiac | High | Low | High | Low | Not reported | Not reported | Low |
| Tritapepe 2007 | 150 | Desflurane | Adults ≥18 | Cardiac | Moderate | Low | Not reported | Low | Low | Low | Low |
| Tsuchiya 2018 | 186 | Sevoflurane | Adults ≥18 | Non-cardiac | Low | Low | Low | Low | Low | Low | Low |
| Van der Linden 2010 | 40 | Sevoflurane | Adults ≥18 | Non-cardiac: vascular | High | Low | High | Low | Not reported | Not reported | Low |
| Vasigh 2017 | 50 | Sevoflurane | Adults ≥18 | Non-cardiac | Low | Low | Low | Low | Low | Low | Low |
| Verma 2022 | 75 | Sevoflurane, desflurane | Adults ≥18 | Non-cardiac | Moderate | Low | Low | Low | Not reported | Low | Low |
| Von Dossow 2007 | 28 | Isoflurane | Adults ≥18 | Non-cardiac | Moderate | Not reported | Not reported | Low | Low | Low | Low |
| Wan Hassan 2017 | 110 | Sevoflurane | Adults ≥18 | Non-cardiac: intracranial | Moderate | Low | Low | Low | Low | Not reported | Low |
| Wang 2017 | 88 | Sevoflurane | Adults ≥18 | Non-cardiac | Moderate | Low | Low | Low | Low | Not reported | Low |
| Wąsowicz 2018 | 127 | Sevoflurane | Adults ≥18 | Cardiac | High | Low | Not reported | High | Not reported | Low | Low |
| Weng 2020 | 120 | Sevoflurane | Children <18 | Non-cardiac | High | Low | Low | High | Not reported | Not reported | Low |
| Wilhelm 1998 | 40 | Desflurane | Adults ≥18 | Non-cardiac | High | Not reported | Not reported | Low | High | High | Low |
| Windpassinger 2016 | 90 | Sevoflurane | Adults ≥18 | Non-cardiac: vascular | Low | Low | Low | Low | Low | Low | Low |
| Wong 2019 | 96 | Sevoflurane | Adults ≥18 | Non-cardiac | Low | Low | Low | Low | Low | Low | Low |
| Wong 2021 | 90 | Sevoflurane | Adults ≥18 | Non-cardiac | Low | Low | Low | Low | Low | Low | Low |
| Wu 2019 | 50 | Desflurane | Adults ≥18 | Non-cardiac | Moderate | Not reported | Low | Not reported | Not reported | Not reported | Low |
| Xia 2006 | 54 | Isoflurane | Adults ≥18 | Cardiac | High | Not reported | High | Low | High | Not reported | Low |
| Xu 2014 | 40 | Sevoflurane | Adults ≥18 | Non-cardiac: pulmonary | High | Not reported | High | Low | Not reported | Not reported | Low |
| Xu 2017 | 80 | Isoflurane | Adults ≥18 | Non-cardiac | Moderate | Low | Not reported | Not reported | Low | Not reported | Low |
| Yan 2019 | 80 | Sevoflurane | Adults ≥18 | Non-cardiac | Moderate | Low | Low | Not reported | Low | Low | Low |
| Yang 2010 | 60 | Isoflurane | Adults ≥18 | Non-cardiac | Low | Low | Low | Low | Low | Low | Low |
| Yang 2021 | 95 | Sevoflurane | Adults ≥18 | Non-cardiac | Moderate | Low | Low | Not reported | Low | Low | Low |
| Yang XL 2017 | 73 | Sevoflurane | Adults ≥18 | Cardiac | High | Low | Not reported | High | Low | Not reported | Low |
| Yang Z 2017 | 60 | Sevoflurane | Children <18 | Non-cardiac | Moderate | Not reported | Not reported | Not reported | Not reported | Not reported | Low |
| Yildrim 2009 | 60 | Sevoflurane, isoflurane | Adults ≥18 | Cardiac | High | Low | High | Low | Low | Low | Low |
| Yoo 2012 | 62 | Desflurane | Adults ≥18 | Non-cardiac | Moderate | Low | Not reported | Low | Not reported | Not reported | Low |
| Yoon 2023 | 140 | Desflurane | Adults ≥18 | Non-cardiac | Low | Low | Low | Low | Low | Low | Low |
| Yu 2017 | 1000 | Sevoflurane | Elderly | Non-cardiac | Moderate | Not reported | Not reported | Not reported | Not reported | Not reported | Low |
| Yu 2021 | 489 | Sevoflurane | Adults ≥18 | Cardiac | Low | Low | Low | Low | Low | Low | Low |
| Zangrillo 2011 | 88 | Sevoflurane | Adults ≥18 | Non-cardiac | Low | Low | Low | Low | Low | Low | Low |
| Zhang 2013 | 80 | Sevoflurane | Adults ≥18 | Non-cardiac | Moderate | Low | Not reported | Low | Not reported | Not reported | Low |
| Zhang 2016 | 80 | Sevoflurane | Elderly | Non-cardiac | High | Low | High | Low | High | High | Low |
| Zhang 2017 | 121 | Sevoflurane | Elderly | Cardiac | High | Low | Low | High | Low | Low | Low |
| Zhang 2018 | 379 | Sevoflurane | Elderly | Non-cardiac | Moderate | Low | Low | Not reported | Low | Low | Low |
| Zhang 2019 | 200 | Sevoflurane | Adults ≥18 | Non-cardiac | Moderate | Low | Not reported | Not reported | Not reported | Not reported | Low |
| Zhao 2017 | 80 | Sevoflurane | Adults ≥18 | Cardiac | High | Low | Not reported | High | High | Not reported | Low |
| Zhao 2018 | 82 | Sevoflurane | Adults ≥18 | Non-cardiac | Moderate | Not reported | Not reported | Not reported | Not reported | Not reported | Low |
| Zhiguo 2019 | 98 | Sevoflurane | Adults ≥18 | Non-cardiac: pulmonary | Moderate | Not reported | Not reported | Not reported | Not reported | Not reported | Low |
| Zhou 2020 | 82 | Sevoflurane | Children <18 | Non-cardiac | High | Low | High | High | Not reported | Not reported | Low |
| Zhou 2021 | 201 | Sevoflurane | Adults ≥18 | Non-cardiac | Low | Low | Low | Low | Low | Low | Low |
| Zhu 2020 | 197 | Desflurane | Adults ≥18 | Non-cardiac | High | Low | Low | High | Low | Low | Low |

**Appendix 9: Subgroup analyses results**

*Supplementary Table 7: Effect estimate results of the subgroup analyses of the meta-analysis. Subgroup analyses are performed for every outcome where at least two subgroup included at least one RCT. Abbreviations: RR risk ratio, MD mean difference, SMD: standardized mean difference, RE random effect, TIVA total intravenous anaesthesia.*

| Outcome measures | No of RCTs | No of patients | Statistical model | Effect estimate (95% CI) | Preference |
| --- | --- | --- | --- | --- | --- |
| Mortality |  |  |  |  |  |
| Mortality in-hospital  Type of anaesthetics   - Sevoflurane vs. propofol - Desflurane vs. propofol - Isoflurane vs. propofol   Age   - General adult population - Elderly adult population   Type of surgery   - Cardiac - Non-cardiac   - Pulmonary   - Intracranial   - Vascular | 27  22  5  2  23  4  12  15  4  3  0 | 3846  3129  597  120  2994  852  1591  2255  662  433  0 | RR, RE | 1.05 (0.67,1.66)  1.14 (0.71,1.82)  0.32 (0.03,3.00)  0.33 (0.01,7.87)  1.13 (0.71,1.81)  0.34 (0.05,2.11)   - 1. (0.45,2.27)   1.07 (0.62,1.86)  0.28 (0.05,1.61)  1.41 (0.52,3.84)  Not estimable | None  None  None  None  None  None  None  None  None  None |
| Mortality 30-day  Type of anaesthetics   - Sevoflurane vs. propofol - Desflurane vs. propofol - Isoflurane vs. propofol - Unspecified vs. propofol   Age   - Children - General adult population - Elderly adult population   Type of surgery   - Cardiac - Non-cardiac   - Pulmonary   - Vascular | 23  15  4  3  3  2  20  1  12  11  2  0 | 9667  2329  591  164  6583  180  9457  30  8147  1520  687  0 | RR, RE | 0.97 (0.70,1.36)  1.15 (0.69,1.93)  1.63 (0.20,13.30)  Not estimable  0.83 (0.53,1.31)  0.33 (0.01,7.87)  0.97 (0.69,1.37)  3.00 (0.13,68.26)  0.95 (0.67,1.35)  1.24 (0.43,3.59)  6.84 (0.33,130.53)  Not estimable | None  None  None  None  None  None  None  None  None  None  None |
| Mortality one-year  Type of anaesthetics   - Sevoflurane vs. propofol - Isoflurane vs. propofol - Unspecified vs. propofol   Age   - General adult population - Elderly adult population   Type of surgery   - Cardiac - Non-cardiac   - Pulmonary   - Vascular | 13  11  1  1  12  1  7  6  2  0 | 9317  3880  84  5353  8979  385  6895  2422  233  0 | RR, RE | 1.14 (0.88,1.48)  1.16 (0.81,1.65)  4.77 (0.24,96.52)  1.06 (0.78,1.45)  1.21 (0.91,1.60)  0.84 (0.50,1.43)  1.17 (0.95,1.45)  1.43 (0.73,2.77)  1.42 (0.76,2.66)  Not estimable | None  None  None  None  None  None  None  None  None |
| Morbidity – Cardiovascular |  |  |  |  |  |
| Myocardial infarction  Type of anaesthetics   - Sevoflurane vs. propofol - Desflurane vs. propofol - Isoflurane vs. propofol - Unspecified vs. propofol   Age   - General adult population - Elderly adult population   Type of surgery   - Cardiac - Non-cardiac | 20  14  5  1  1  16  5  15  5 | 9336  3292  560  84  5400  7425  1911  7382  1954 | RR, RE | 1.15 (0.88,1.49)  1.10 (0.65,1.86)  1.91 (0.97,3.75)  4.77 (0.24, 96.52)  1.01 (0.72,1.41)  1.16 (0.88,1.52)  1.02 (0.41,2.54)  1.17 (0.89,1.53)  0.89 (0.31,2.59) | None  None  None  None  None  None  None  None  None |
| Atrial fibrillation  Type of anaesthetics   - Sevoflurane vs. propofol - Desflurane vs. propofol - Isoflurane vs. propofol   Age   - General adult population - Elderly adult population   Type of surgery   - Cardiac - Non-cardiac | 15  13  2  1  14  1  13  2 | 1855  1501  270  84  1621  234  1533  322 | RR, RE | 1.01 (0.82,1.24)  0.94 (0.72,1.23)  1.09 (0.72,1.64)  1.63 (0.71,3.74)  1.01 (0.81,1.26)  1.00 (0.14,6.98)  1.02 (0.80,1.29)  1.00 (0.20,4.88) | None  None  None  None  None  None  None  None |
| Myocardial injury  Type of anaesthetics   - Sevoflurane vs. propofol - Desflurane vs. propofol - Isoflurane vs. propofol   Type of surgery   - Cardiac - Non-cardiac | 4  3  1  1  2  2 | 811  631  120  60  300  511 | RR, RE | 1.44 (1.00,2.07)  1.45 (0.87,2.41)  1.81 (1.30,2.52)  0.88 (0.36,2.11)  1.76 (1.27,2.44)  1.16 (0.76,1.77) | None  None  **Inhalation**  None  **Inhalation**  None |
| Pulmonary embolism | 4 | 3106 | RR, RE | 1.14 (0.27,4.87) | None |
| Cardiovascular death  Type of surgery   - Cardiac - Non-cardiac | 3  1  2 | 6980  5400  1580 | RR, RE | 1.56 (0.89,2.74)  1.73 (0.89,3.33)  1.20 (0.41,3.51) | None  None  None |
| Deep vein thrombosis | 3 | 3099 | RR, RE | 0.95 (0.44,2.05) | None |
| Major adverse cardiac events | 2 | 559 | RR, RE | 1.02 (0.58,1.78) | None |
| Non-fatal cardiac event | 0 | 0 |  | Not estimable |  |
| Coronary revascularisation | 0 | 0 |  | Not estimable |  |
| Morbidity – Pulmonary |  |  |  |  |  |
| Pneumonia  Type of anaesthetics   - Sevoflurane vs. propofol - Desflurane vs. propofol - Isoflurane vs. propofol - Unspecified vs. propofol   Age   - General adult population - Elderly adult population   Type of surgery   - Cardiac - Non-cardiac   - Pulmonary   - Intracranial   - Vascular | 23  15  5  2  2  20  3  8  15  6  1  1 | 5011  3160  590  78  1183  3169  1842  1725  3286  867  110  40 | RR, RE | 1.01 (0.87,1.18)  1.04 (0.79,1.37)  1.21 (0.76,1.93)  1.52 (0.30,7.65)  0.94 (0.76,1.17)  1.02 (0.87,1.20)  0.83 (0.40,1.73)  0.93 (0.75,1.15)  1.11 (0.89,1.39)  1.12 (0.83,1.51)  1.43 (0.59,3.48)  0.20 (0.01,3.92) | None  None  None  None  None  None  None  None  None  None  None  None |
| Atelectasis  Type of anaesthetics   - Sevoflurane vs. propofol - Desflurane vs. propofol - Isoflurane vs. propofol - Unspecified vs. propofol   Type of surgery   - Cardiac - Non-cardiac   - Pulmonary   - Intracranial | 10  6  3  1  1  2  8  3  1 | 1736  792  380  50  514  100  1636  741  514 | RR, RE | 1.00 (0.75,1.34)  1.03 (0.58,1.84)  1.36 (0.48,3.81)  2.50 (0.53,11.70)  0.89 (0.63,1.26)  1.70 (0.66,4.37)  0.95 (0.69,1.31)  1.15 (0.54,2.46)  0.89 (0.63,1.26) | None  None  None  None  None  None  None  None  None |
| ARDS  Type of surgery   - Cardiac - Non-cardiac | 4  2  2 | 166  86  80 | RR, RE | 3.72 (0.43,32.17)  4.50 (0.23,87.61)  3.00 (0.13,69.52) | None  None  None |
| Aspiration | 2 | 687 |  | Not estimable |  |
| Morbidity – Neurological |  |  |  |  |  |
| Postoperative cognitive dysfunction score  Type of anaesthetics   - Sevoflurane vs. propofol - Desflurane vs. propofol - Isoflurane vs. propofol   Age of patients   - Children <18 years old - General adult population - Elderly adult population   Type of surgery   - Cardiac - Non-cardiac   - Pulmonary   - Intracranial   - Vascular   Type of cognitive test   - MMSE - Other test | 31  20  8  6  1  21  9  2  29  2  2  1  19  12 | 6539  3751  480  2308  60  1562  4917  184  6355  160  140  44  4480  2059 | SMD, RE | 0.58 (0.05,1.11)  0.77 (-0.03,1.58)  0.08 (-0.24,0.40)  0.61 (-0.45,1.68)  0.44 (-0.10,0.99)  0.14 (-0.14,0.43)  1.68 (0.47,2.88)  -0.13 (-1.82,1.56)  0.63 (0.08,1.18)  0.56 (-0.59,1.71)  0.46 (0.07,0.86)  0.40 (-0.20,1.00)  0.56 (-0.13,1.26)  0.59 (-0.10,1.28) | **TIVA**  None  None  None  None  None  **TIVA**  None  **TIVA**  None  **TIVA**  None  None  None |
| Postoperative cognitive dysfunction incidence  Type of anaesthetics   - Sevoflurane vs. propofol - Desflurane vs. propofol - Isoflurane vs. propofol - Unspecified vs. propofol   Age   - General adult population - Elderly adult population   Type of surgery   - Cardiac - Non-cardiac   - Vascular | 19  13  4  2  1  8  11  3  16  1 | 10,074  2338  261  2075  5400  6240  3834  5799  4275  44 | RR, RE | 0.78 (0.58,1.05)  0.85 (0.65,1.10)  1.00 (0.71,1.39)  0.03 (0.00,3.18)  1.11 (0.68,1.81)  1.07 (0.78,1.47)  0.62 (0.40,0.97)  1.03 (0.62,1.70)  0.73 (0.51,1.06)  0.30 (0.09,0.98) | None  None  None  None  None  None  **TIVA**  None  None  **TIVA** |
| Postoperative delirium  Type of anaesthetics   - Sevoflurane vs. propofol - Desflurane vs. propofol - Unspecified vs. propofol   Age   - General adult population - Elderly adult population   Type of surgery   - Cardiac - Non-cardiac   - Vascular | 15  11  2  2  7  8  5  10  1 | 9766  3489  208  6069  7142  2624  6778  2988  120 | RR, RE | 0.92 (0.74,1.14)  0.94 (0.75,1.19)  1.00 (0.43,2.34)  0.81 (0.37,1.79)  0.92 (0.70,1.20)  0.91 (0.62,1.34)  0.87 (0.58,1.30)  0.95 (0.72,1.24)  Not estimable | None  None  None  None  None  None  None  None |
| Cerebrovascular event / stroke  Type of surgery   - Cardiac - Non-cardiac   Age   - General adult population - Elderly adult population | 10  5  5  7  3 | 9634  6301  2333  8030  1604 | RR, RE | 0.67 (0.42,1.08)  0.62 (0.36,1.08)  0.82 (0.33,2.04)  0.60 (0.36,1.03)  1.00 (0.35,2.84) | None  None  None  None  None |
| Morbidity – Renal |  |  |  |  |  |
| Acute kidney injury / AKI  Type of anaesthetics   - Sevoflurane vs. propofol - Desflurane vs. propofol - Unspecified vs. propofol   Age of patients   - Children <18 years old - General adult population - Elderly adult population   Type of surgery   - Cardiac - Non-cardiac   - Pulmonary | 11  7  3  1  1  9  1  6  5  2 | 7683  1881  402  5400  120  6368  1195  5995  1688  233 | RR, RE | 0.97 (0.76,1.23)  0.96 (0.64,1.43)  0.92 (0.56,1.52)  0.91 (0.58,1.41)  1.65 (1.01,2.67)  0.84 (0.66,1.08)  1.05 (0.68,1.64)  0.85 (0.63,1.13)  1.06 (0.69,1.64)  0.78 (0.38,1.57) | None  None  None  None  **Inhalation**  None  None  None  None  None |
| Renal Replacement therapy / RRT | 3 | 5559 | RR, RE | 0.68 (0.29,1.60) | None |
| Morbidity – anaesthetic & surgical |  |  |  |  |  |
| Postoperative nausea and vomiting  Type of anaesthetics   - Sevoflurane vs. propofol - Desflurane vs. propofol - Isoflurane vs. propofol - Unspecified vs. propofol   Age of patients   - Adults ≥18 years old - Children <18 years old   Type of surgery   - Cardiac - Non-cardiac   - Pulmonary   - Intracranial   - Vascular | 145  96  38  21  1  131  14  4  141  3  11  3 | 23,172  12,553  2914  2544  5161  21,905  1267  350  22,822  655  1272  257 | RR, RE | 0.61 (0.56,0.67)  0.58 (0.51,0.66)  0.65 (0.57,0.75)  0.65 (0.50,0.83)  0.81 (0.75,0.88)  0.60 (0.55,0.67)  0.55 (0.36,0.84)  0.87 (0.50,1.49)  0.61 (0.56,0.66)  0.65 (0.43,1.00)  0.48 (0.37,0.62)  0.72 (0.38,1.38) | **TIVA**  **TIVA**  **TIVA**  **TIVA**  **TIVA**  **TIVA**  **TIVA**  None  **TIVA**  None  **TIVA**  None |
| Emergence delirium  Type of anaesthetics   - Sevoflurane vs. propofol - Desflurane vs. propofol - Isoflurane vs. propofol   Age of patients   - General adults population - Children <18 years old   Type of surgery   - Cardiac - Non-cardiac   - Intracranial | 32  29  3  1  20  12  4  28  4 | 4203  3979  200  24  3209  948  248  3955  553 | RR, RE | 0.40 (0.29,0.56)  0.41 (0.29,0.59)  0.30 (0.15,0.60)  0.14 (0.01,2.50)  0.50 (0.34,0.73)  0.27 (0.15,0.50)  0.71 (0.36,1.38)  0.37 (0.26,0.53)  0.71 (0.19,2.66) | **TIVA**  **TIVA**  **TIVA**  None  **TIVA**  **TIVA**  None  **TIVA**  None |
| Postoperative shivering  Type of anaesthetics   - Sevoflurane vs. propofol - Desflurane vs. propofol - Isoflurane vs. propofol   Type of surgery   - Cardiac - Non-cardiac   - Intracranial   - Other | 27  16  5  8  1  26  4  22 | 3786  1970  266  1550  354  3432  732  2700 | RR, RE | 1.08 (0.96,1.23)  1.12 (0.95,1.31)  1.16 (0.76,1.77)  0.91 (0.70,1.18)  1.19 (0.81,1.75)  1.07 (0.94,1.22)  1.36 (0.86,2.17)  1.01 (0.88,1.17) | None  None  None  None  None  None  None  None |
| Accidental awakenings  Type of anaesthetics   - Sevoflurane vs. propofol - Desflurane vs. propofol - Isoflurane vs. propofol   Type of surgery   - Cardiac - Non-cardiac | 6  2  1  3  1  5 | 623  193  160  270  73  550 | RR, RE | 1.53 (0.41,5.68)  Not estimable  Not estimable  1.53 (0.41,5.68)  Not estimable  1.53 (0.41,5.68) | None  None  None |
| QoR-40 / Quality of Recovery  Type of anaesthetics   - Sevoflurane vs. propofol - Desflurane vs. propofol   Type of surgery   - Cardiac - Non-cardiac | 17  11  6  1  16 | 1835  1331  504  95  1745 | MD, RE | 6.45 (3.64,9.25)  6.06 (2.08,10.04)  7.66 (2.56,12.76)  0.95 (-6.41,8.31)  6.74 (3.85,9.63) | **TIVA**  **TIVA**  **TIVA**  None  **TIVA** |
| Qor-15 / Quality of Recovery  Type of anaesthetics   - Sevoflurane vs. propofol - Desflurane vs. propofol - Isoflurane vs. propofol - Unspecified vs. propofol   Type of surgery   - Cardiac - Non-cardiac | 4  1  1  1  1  1  3 | 792  489  140  80  83  489  303 | MD, RE | 4.00 (-1.17,9.18)  -0.40 (-1.66,0.86)  7.00 (-1.41,15.41)  3.90 (-2.34,10.14)  11.00 (1.27,20.73)  -0.40 (-1.66,0.86)  6.26 (1.80,10.71) | None  None  None  None  **TIVA**  None  **TIVA** |
| First pain score <12 hours (0-10 VAS)  Type of anaesthetics   - Sevoflurane vs. propofol - Desflurane vs. propofol - Isoflurane vs. propofol   Age of patients   - Adults ≥18 years old - Children <18 years old   Type of surgery   - Cardiac - Non-cardiac   - Pulmonary   - Intracranial | 67  50  15  7  64  3  3  64  1  2 | 6333  4935  993  405  6073  260  219  6114  36  176 | MD, RE | -0.18 (-0.39,0.04)  -0.18 (-0.45,0.08)  -0.21 (-0.57,0.16)  -0.01 (-0.61,0.60)  -0.16 (-0.38,0.06)  -0.46 (-0.87,-0.06)  -0.35 (-1.35,0.65)  -0.17 (-0.39,0.05)  0.40 (-1.17,1.97)  -0.34 (-1.00,0.33) | None  None  None  None  None  **TIVA**  None  None  None  None |
| Pain score 12-24 hours (0-10 VAS)  Type of anaesthetics   - Sevoflurane vs. propofol - Desflurane vs. propofol - Isoflurane vs. propofol - Unspecified vs. propofol   Type of surgery   - Cardiac - Non-cardiac | 44  33  9  4  1  4  40 | 5168  3594  717  188  669  1337  3831 | MD, RE | -0.13 (-0.30,0.04)  -0.10 (-0.35,0.16)  -0.09 (-0.24,0.05)  -0.59 (-0.76,-0.41)  0.00 (-0.34,0.34)  -0.19 (-0.42,0.04)  -0.11 (-0.30,0.07) | None  None  None  **TIVA**  None  None  None |
| Pain score >24 hours (0-10 VAS)  Type of anaesthetics   - Sevoflurane vs. propofol - Desflurane vs. propofol - Isoflurane vs. propofol - Unspecified vs. propofol   Type of surgery   - Cardiac - Non-cardiac | 22  15  5  2  1  3  20 | 3250  1919  558  104  669  1253  1997 | MD, RE | -0.24 (-0.51,0.03)  -0.22 (-0.68,0.25)  -0.09 (-0.14,-0.05)  -1.21 (-1.44,-0.98)  0.00 (-0.23,0.23)  -0.03 (-0.20,0.14)  -0.28 (-0.59,0.03) | None  None  **TIVA**  **TIVA**  None  None  None |
| Clavien-Dindo grades ≥3  Type of anaesthetics   - Sevoflurane vs. propofol - Desflurane vs. propofol   Type of surgery   - Cardiac - Non-cardiac   - Pulmonary | 8  6  3  0  8  2 | 1389  909  480  0  1389  687 | RR, RE | 1.25 (0.92,1.69)  1.29 (0.93,1.78)  1.00 (0.41,2.42)  Not estimable  1.25 (0.92,1.69)  1.17 (0.71,1.94) | None  None  None  None  None |
| Major bleeding  Type of anaesthetics   - Sevoflurane vs. propofol - Desflurane vs. propofol - Unspecified vs. propofol   Type of surgery   - Cardiac - Non-cardiac | 6  4  2  1  3  3 | 7076  1444  232  5400  5752  1324 | RR, RE | 0.86 (0.51,1.44)  0.58 (0.34,1.00)  0.72 (0.13,3.85)  1.37 (0.89,2.10)  1.33 (0.88,2.00)  0.56 (0.32,0.96) | None  None  None  None  None  **TIVA** |
| Surgical site infection  Type of anaesthetics   - Sevoflurane vs. propofol - Desflurane vs. propofol | 5  4  1 | 1769  1699  70 | RR, RE | 0.89 (0.55,1.44)  0.88 (0.53,1.45)  1.00 (0.22,4.62) | None  None  None |
| Cancer recurrence | 3 | 2204 | RR, RE | 0.93 (0.73,1.19) | None |
| WHODAS 1.0 or 2.0 | 0 | 0 |  | Not estimable |  |
| Efficiency – anaesthetic & general |  |  |  |  |  |
| Intraoperative opioid consumption  Type of anaesthetics   - Sevoflurane vs. propofol - Desflurane vs. propofol - Isoflurane vs. propofol   Age of patients   - Adults ≥18 years old - Children <18 years old   Type of surgery   - Cardiac - Non-cardiac   - Pulmonary   - Intracranial | 80  55  26  8  79  1  8  72  1  10 | 11,309  8747  2004  558  11,309  40  1145  10,164  36  951 | SMD, RE | 0.35 (0.18,0.52)  0.41 (0.18,0.65)  0.32 (0.12,0.51)  0.09 (-0.15,0.34)  0.36 (0.19,0.53)  -0.42 (-1.04,0.21)  0.25 (0.11,0.38)  0.37 (0.18,0.56)  3.48 (2.40,4.55)  0.78 (0.28,1.28) | **Inhalation**  **Inhalation**  **Inhalation**  None  **Inhalation**  None  **Inhalation**  **Inhalation**  **Inhalation**  **Inhalation** |
| Postoperative opioid consumption  Type of anaesthetics   - Sevoflurane vs. propofol - Desflurane vs. propofol - Isoflurane vs. propofol   Age of patients   - Adults ≥18 years old - Children <18 years old   Type of surgery   - Cardiac - Non-cardiac   - Pulmonary   - Intracranial | 37  28  11  1  36  1  4  33  1  2 | 6614  5466  971  177  6514  100  606  6008  36  170 | SMD, RE | 0.02 (-0.15, 0.20)  0.08 (-0.08,0.23)  -0.02 (-0.51,0.47)  -0.95 (-1.28,-0.62)  0.04 (-0.13,0.22)  -0.66 (-1.06,-0.26)  -0.05 (-0.71,0.62)  0.04 (-0.14,0.21)  0.72 (0.05,1.40)  0.00 (-0.31,0.31) | None  None  None  **TIVA**  None  **TIVA**  None  None  **Inhalation**  None |
| Time to extubation (minutes)  Type of anaesthetics   - Sevoflurane vs. propofol - Desflurane vs. propofol - Isoflurane vs. propofol   Age of patients   - Adults ≥18 years old - Children <18 years old   Type of surgery   - Cardiac - Non-cardiac   - Pulmonary   - Intracranial | 113  63  36  25  103  10  12  101  2  13 | 11,433  6317  2438  2708  10,591  872  1050  10,413  142  1172 | MD, RE | 0.53 (-0.07,1.14)  0.28 (-0.64,1.20)  1.11 (0.21,2.01)  0.27 (-0.88,1.42)  0.42 (-0.22,1.06)  1.66 (-0.23,3.54)  13.1 (-24.4,50.5)  0.44 (-0.16,1.03)  -2.13 (-7.20,2.95)  2.36 (0.81,3.91) | None  None  None  None  None  None  None  None  None  **Inhalation** |
| Time to awakening (minutes)  Type of anaesthetics   - Sevoflurane vs. propofol - Desflurane vs. propofol - Isoflurane vs. propofol   Age of patients   - Adults ≥18 years old - Children <18 years old   Type of surgery   - Cardiac - Non-cardiac   - Pulmonary   - Intracranial | 69  43  23  10  66  10  2  67  2  11 | 6753  4539  1695  519  6083  670  151  6602  142  967 | MD, RE | 0.49 (-0.09,1.06)  0.74 (-0.01,1.48)  0.71 (-0.03,1.46)  -2.03 (-3.84,-0.21)  0.30 (-0.30,0.91)  1.86 (-0.17,3.89)  -0,67 (-1.90,0.56)  0.51 (-0.07,1.09)  -2.58 (-8.71,3.55)  1.20 (-0.21,2.61) | None  None  None  **TIVA**  None  None  None  None  None  None |
| Time to follow simple instruction  Type of anaesthetics   - Sevoflurane vs. propofol - Desflurane vs. propofol - Isoflurane vs. propofol   Age of patients   - Adults ≥18 years old - Children <18 years old   Type of surgery   - Cardiac - Non-cardiac   - Pulmonary   - Intracranial | 42  22  14  10  39  3  0  42  1  10 | 4056  1699  913  1444  3896  160  0  4056  62  624 | MD, RE | 0.78 (0.15,1.41)  0.47 (-0.48,1.42)  1.66 (0.49,2.83)  0.29 (-0.80,1.39)  0.86 (0.27,1.45)  -0.32 (-3.23,2.59)  Not estimable  0.78 (0.15,1.41)  -6.00 (-10.30,-1.70)  1.65 (0.01,3.29) | **Inhalation**  None  **Inhalation**  None  **Inhalation**  None  **Inhalation**  **TIVA**  **Inhalation** |
| Time to respiratory recovery  Type of anaesthetics   - Sevoflurane vs. propofol - Desflurane vs. propofol - Isoflurane vs. propofol   Age of patients   - Adults ≥18 years old - Children <18 years old   Type of surgery   - Cardiac - Non-cardiac   - Intracranial | 31  16  10  7  29  2  0  31  6 | 4360  2083  601  1616  4118  182  0  4360  404 | MD, RE | 0.99 (0.04,1.95)  0.64 (-0.79,2.07)  1.89 (0.57,3.20)  0.40 (-0.74,1.55)  0.92 (-0.07,1.90)  2.09 (-0.17,4.34)  Not estimable  0.99 (0.04,1.95)  2.15 (-0.24,4.54) | **Inhalation**  None  **Inhalation**  None  None  None  **Inhalation**  None |
| Time to reach a recovery score  Type of anaesthetics   - Sevoflurane vs. propofol - Desflurane vs. propofol - Isoflurane vs. propofol   Age of patients   - Adults ≥18 years old - Children <18 years old   Type of surgery   - Cardiac - Non-cardiac   - Intracranial | 28  16  11  4  23  5  0  28  3 | 3617  1655  700  1262  3135  482  0  3617  512 | MD, RE | 0.38 (-1.72,2.47)  -0.42 (-3.16,2.32)  0.79 (-2.26,3.84)  2.59 (0.19,4.99)  0.52 (-1.71,2.75)  -0.46 (-8.12,7.12)  Not estimable  0.38 (-1.72,2.47)  3.70 (0.31,7.08) | None  None  None **Inhalation**  None  None  None  **Inhalation** |
| Time to orientation  Type of anaesthetics   - Sevoflurane vs. propofol - Desflurane vs. propofol - Isoflurane vs. propofol   Type of surgery   - Cardiac - Non-cardiac   - Pulmonary   - Intracranial | 26  15  10  5  0  26  1  2 | 2254  1045  835  374  0  2254  80  156 | MD, RE | 2.00 (1.02,2.98)  2.05 (0.91,3.20)  3.73 (1.30,6.17)  -3.36 (-8.04,1.32)  Not estimable  2.00 (1.02,2.98)  0.41 (-1.08,1.90)  6.09 (0.76,11.42) | **Inhalation**  **Inhalation**  **Inhalation**  None  **Inhalation**  None  **Inhalation** |
| PACU time (minutes)  Type of anaesthetics   - Sevoflurane vs. propofol - Desflurane vs. propofol - Isoflurane vs. propofol   Age of patients   - Adults ≥18 years old - Children <18 years old   Type of surgery   - Cardiac - Non-cardiac   - Intracranial | 48  29  16  6  43  5  0  48  2 | 5627  3104  1434  1089  5069  558  0  5627  151 | MD, RE | -1.59 (-3.15,-0.03)  -1.37 (-3.91,1.16)  -2.47 (-5.29,0.35)  0.11 (-2.40,2.63)  -1.89 (-3.41,-0.37)  0.23 (-6.93,7.39)  Not estimable  -1.59 (-3.15,-0.03)  -2.25 (-14.6,10.1) | **TIVA**  None  None  None  **TIVA**  None  **TIVA**  None |
| Length of hospital stay / LoS (days)  Type of anaesthetics   - Sevoflurane vs. propofol - Desflurane vs. propofol - Isoflurane vs. propofol - Unspecified vs. propofol   Age of patients   - Adults ≥18 years old - Children <18 years old   Type of surgery   - Cardiac - Non-cardiac   - Pulmonary   - Intracranial | 75  50  18  7  3  72  3  29  46  7  5 | 18,706  9706  2001  416  6583  18,445  261  10,223  8483  931  392 | MD, RE | 0.31 (0.05,0.57)  0.35 (-0.15,0.84)  0.00 (-0.21,0.21)  0.47 (-0.25,1.19)  0.00 (-0.16,0.16)  0.32 (0.05,0.58)  0.08 (-1.64,1.79)  0.63 (-0.04,1.29)  0.04 (-0.14,0.23)  0.34 (-1.94,2.36)  0.71 (0.28,1.13) | **Inhalation**  None  None  None  None  **Inhalation**  None  None  None  None **Inhalation** |
| Unplanned hospital (re)admissions  Type of anaesthetics   - Sevoflurane vs. propofol - Desflurane vs. propofol - Isoflurane vs. propofol - Unspecified vs. propofol   Type of surgery   - Cardiac - Non-cardiac | 7  4  1  1  1  4  3 | 6039  495  60  84  5400  5735  304 | RR, RE | 0.89 (0.65,1.22)  0.61 (0.34,1.10)  0.33 (0.04,3.03)  0.64 (0.11,3.61)  1.10 (0.77,1.57)  0.87 (0.54,1.43)  0.63 (0.28,1.42) | None  None  None  None  None  None  None |
| Unplanned ICU admission  Type of anaesthetic   - Sevoflurane vs. propofol - Desflurane vs. propofol   Age of patients   - Adults ≥18 years old - Children <18 years old   Type of surgery   - Cardiac - Non-cardiac | 5  5  2  4  1  2  3 | 1338  958  380  1278  60  300  1038 | RR, RE | 0.90 (0.66,1.24)  0.89 (0.65,1.23)  1.40 (0.23,8.46)  0.91 (0.66,1.25)  0.33 (0.01,7.87)  1.22 (0.23,6.40)  0.89 (0.65,1.23) | None  None  None  None  None  None  None |
| Cost analysis (Euro) | 4 | 480 | MD, RE | -1.51 (-8.83,5.81) | None |

**Appendix 10: Forest plots and funnel plots for each prespecified outcome**

Forest and funnel plots are provided for each prespecified outcome, including the main subgroup analysis that compares patients undergoing cardiac surgery with patients undergoing non-cardiac surgery. The forest plots for additional subgroups are provided if their results deviated significantly from the overall results.

**Contents**

1. Mortality in-hospital
2. Mortality 30-day
3. Mortality one-year
4. Myocardial infarction
5. Atrial fibrillation
6. Myocardial injury
7. Cardiovascular death
8. Major adverse cardiac events / MACE
9. Pulmonary embolism
10. Deep vein thrombosis
11. Pneumonia
12. Atelectasis
13. Acute respiratory distress syndrome / ARDS
14. Aspiration
15. Postoperative cognitive dysfunction / POCD score
16. Postoperative cognitive dysfunction / POCD incidence
17. Postoperative delirium
18. Cerebrovascular event / stroke
19. Acute kidney injury / AKI
20. Renal replacement therapy / RRT
21. Postoperative nausea and vomiting / PONV
22. Agitation upon emergence
23. Postoperative shivering
24. Awareness / accidental awakening
25. Qor-40
26. QoR-15
27. First pain score <12 hours postoperatively
28. Pain score 12-24 hours postoperatively
29. Pain score >24 hours postoperatively
30. Clavien-Dindo grades ≥3
31. Major bleeding
32. Surgical site infection
33. Cancer recurrence
34. Intraoperative opioid consumption
35. Postoperative opioid consumption
36. Time to extubation
37. Time to awakening
38. Time to follow simple instruction
39. Time to respiratory recovery
40. Time to reach a recovery score
41. Time to orientation
42. PACU time
43. Length of hospital stay / LoS
44. Unplanned hospital (re)admissions
45. Unplanned ICU admissions
46. Cost analysis
47. Mortality in-hospital


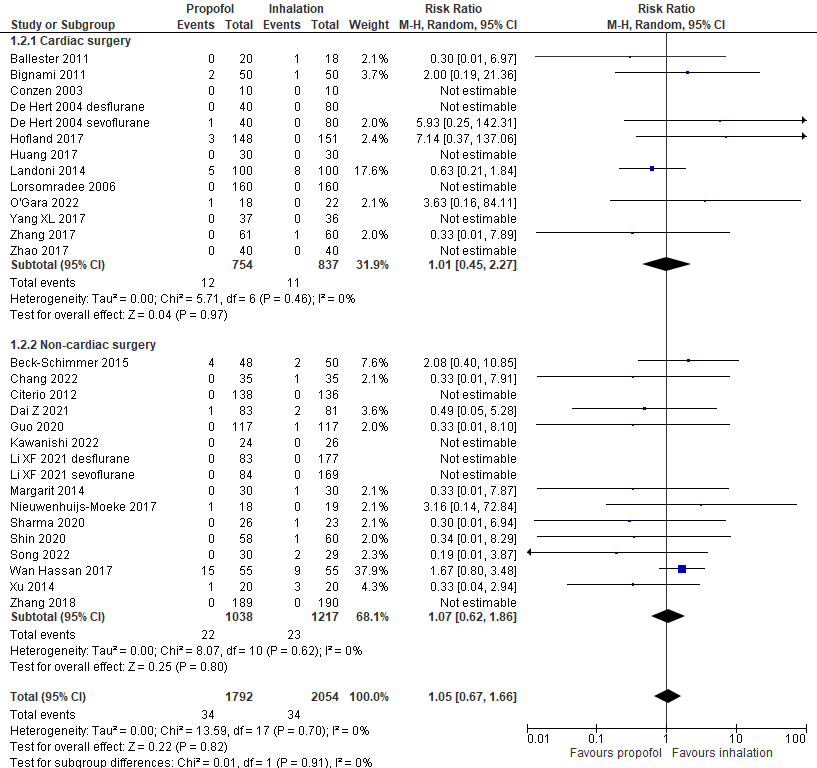


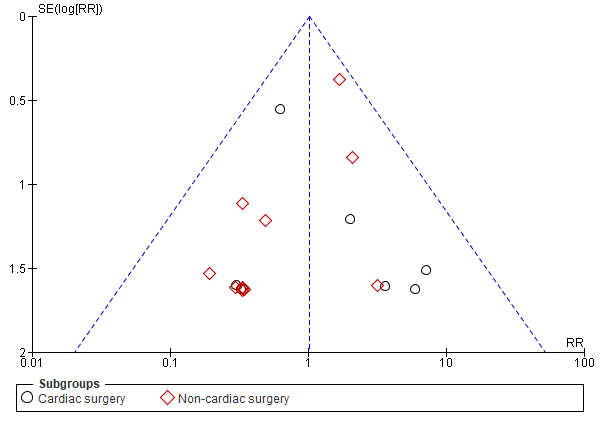


1. Mortality 30-day


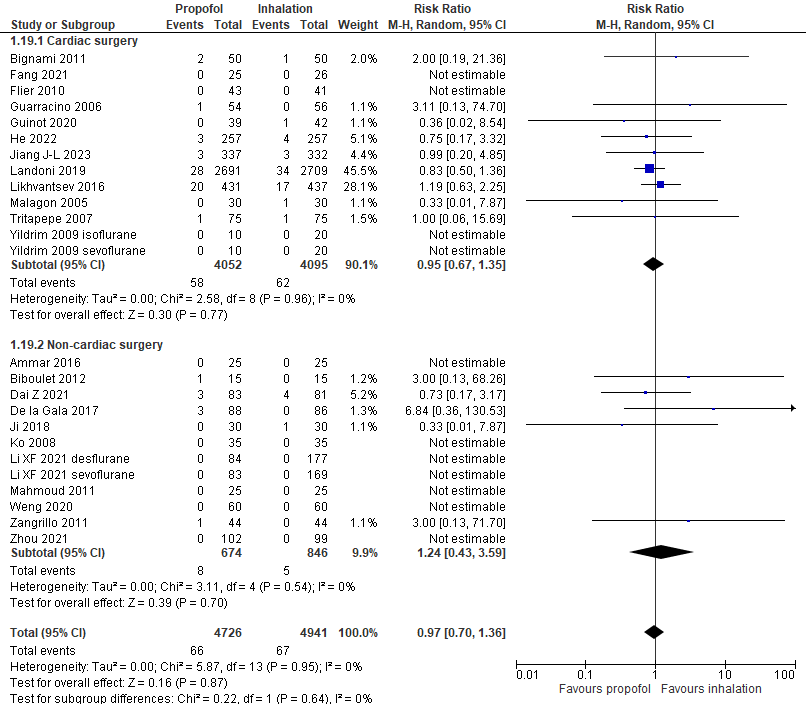


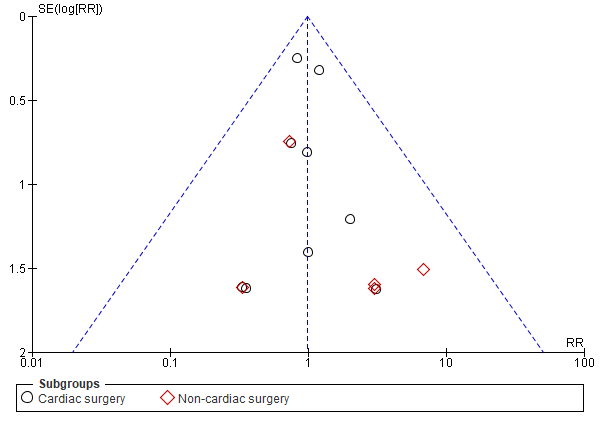


1. Mortality one-year


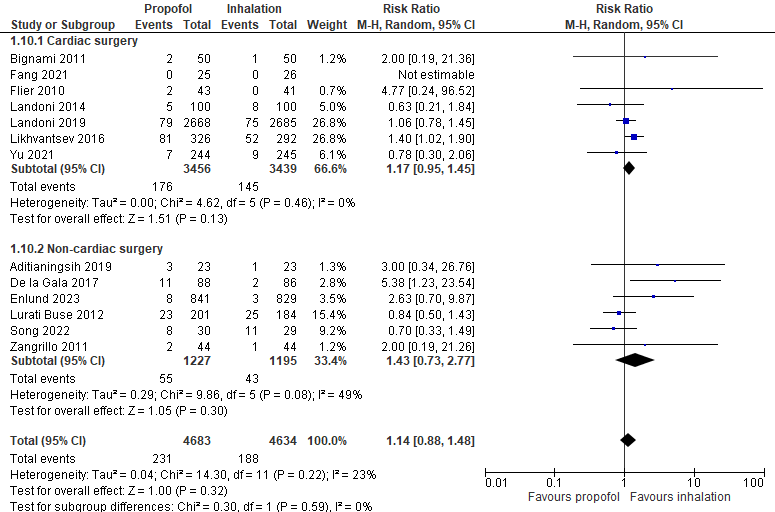


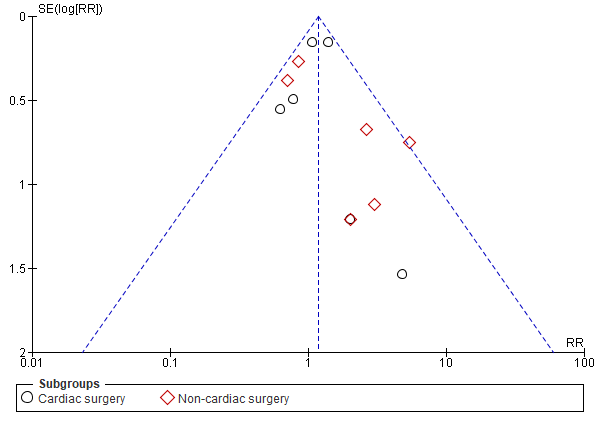


1. Myocardial infarction


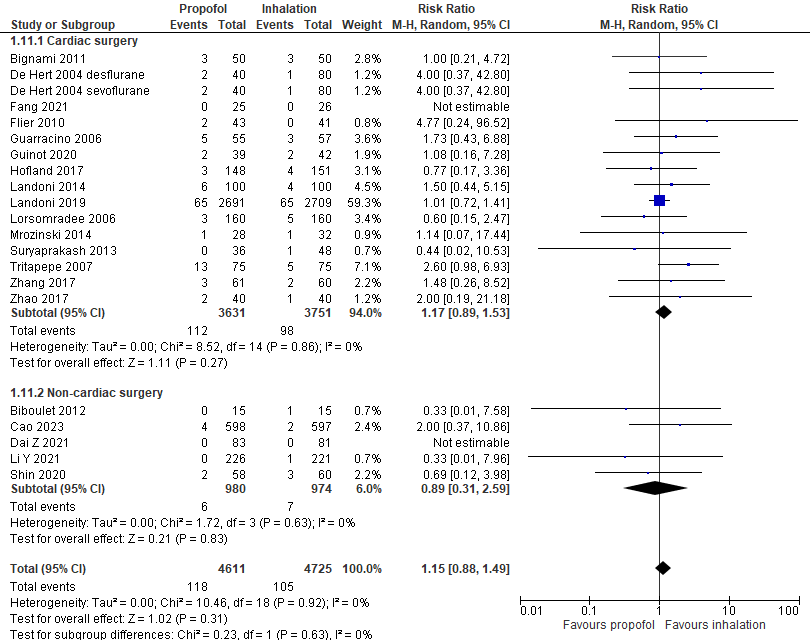


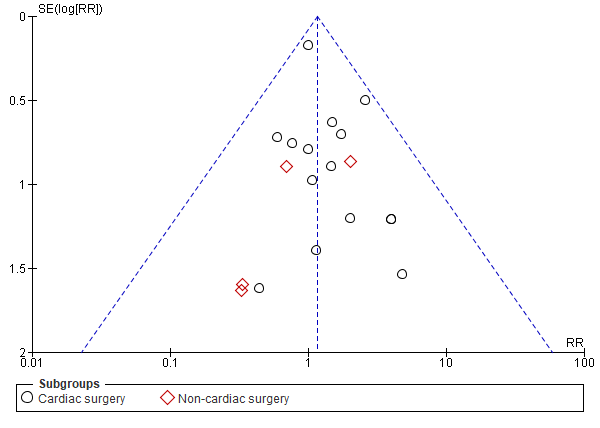


1. Atrial fibrillation


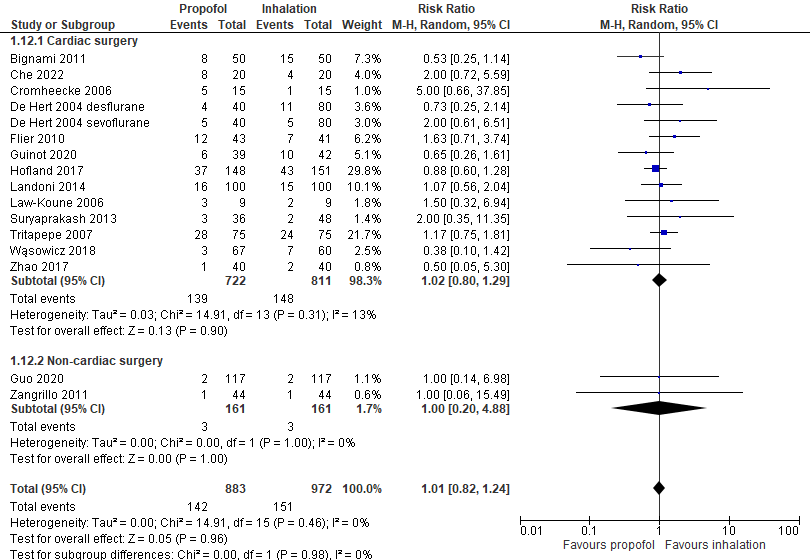


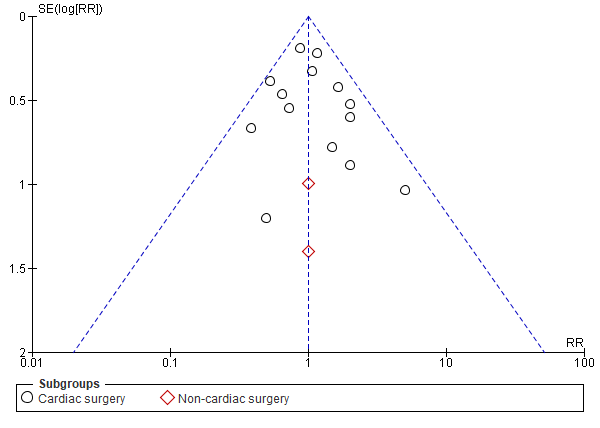


1. Myocardial injury


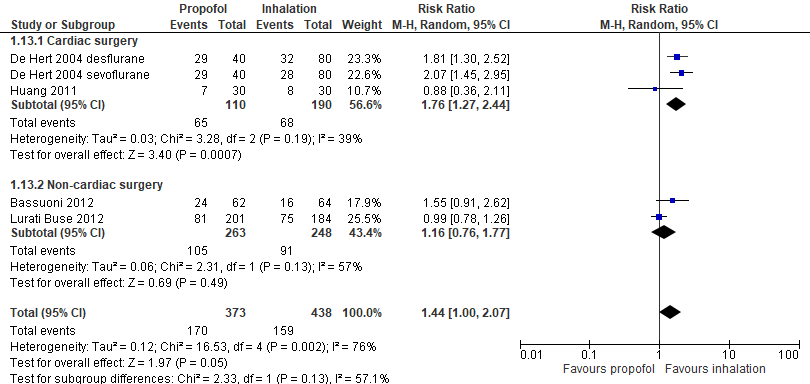


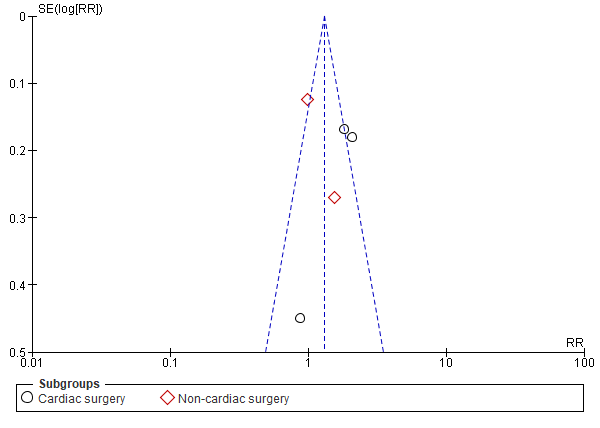


1. Cardiovascular death


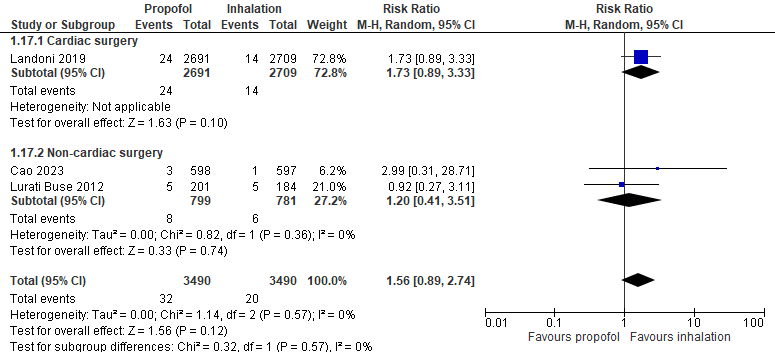


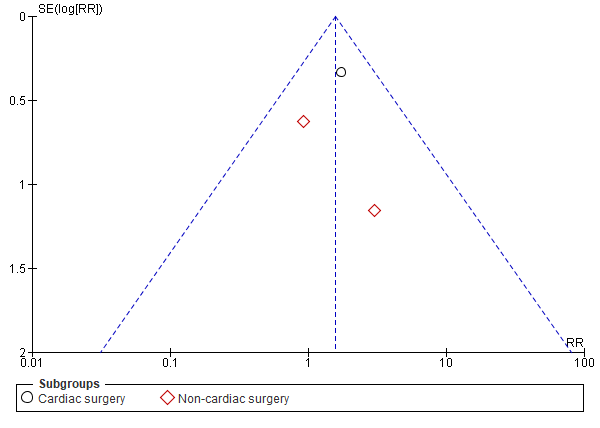


1. Major adverse cardiac events / MACE


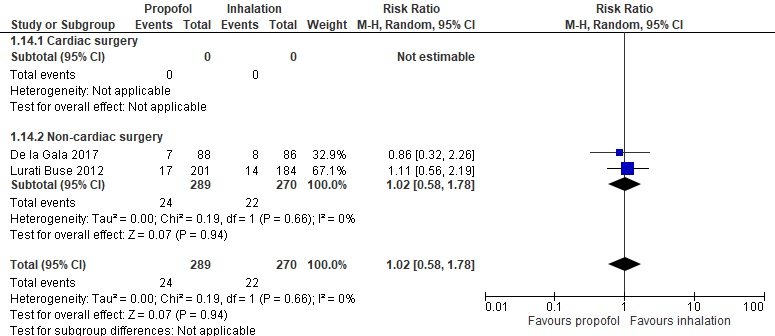


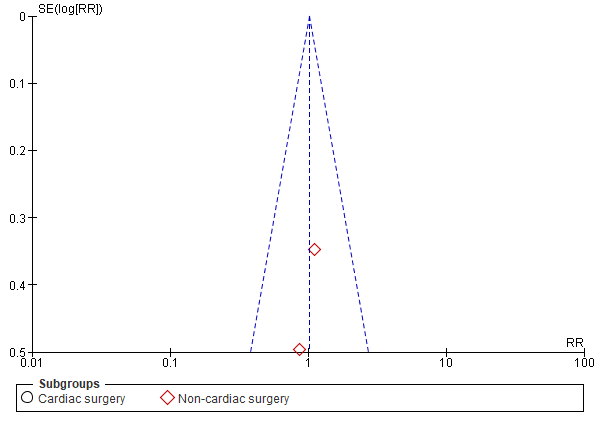


1. Pulmonary embolism


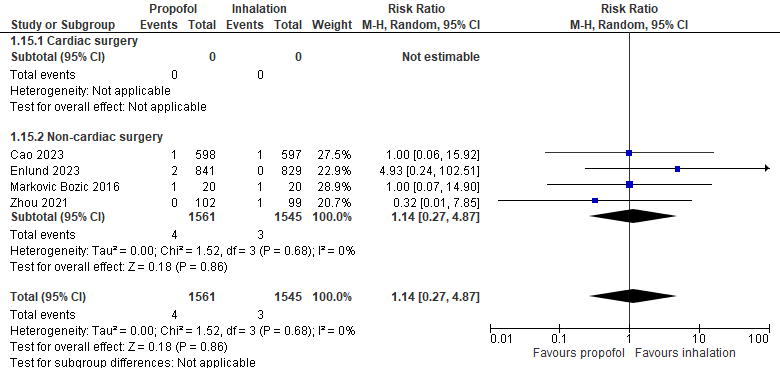


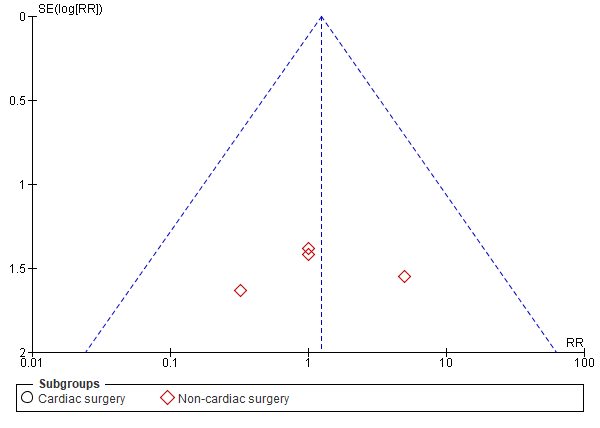


1. Deep vein thrombosis


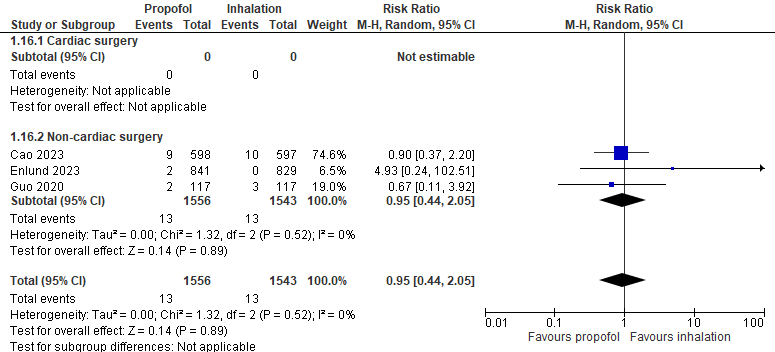


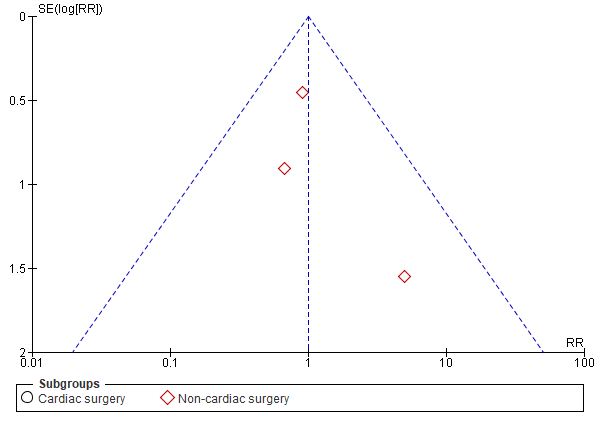


1. Pneumonia


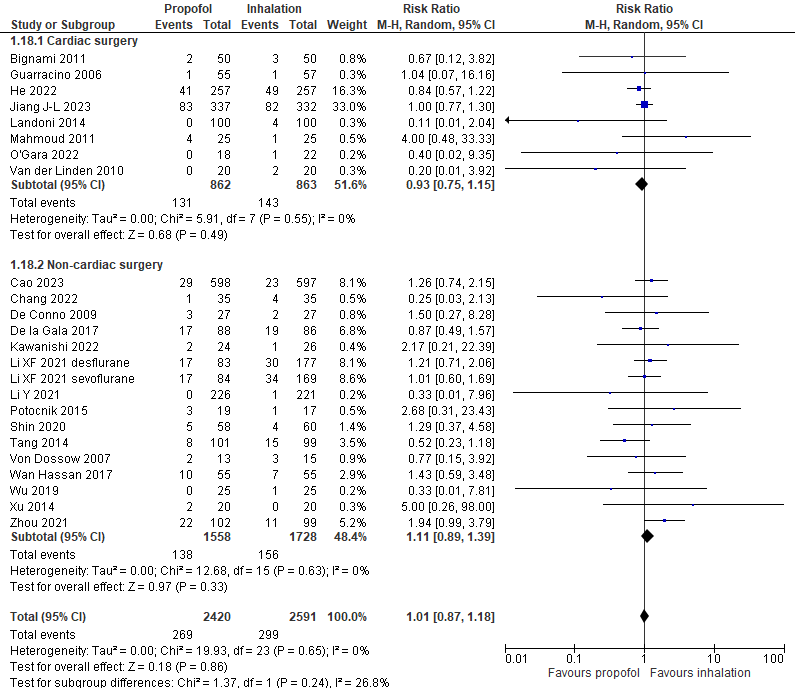


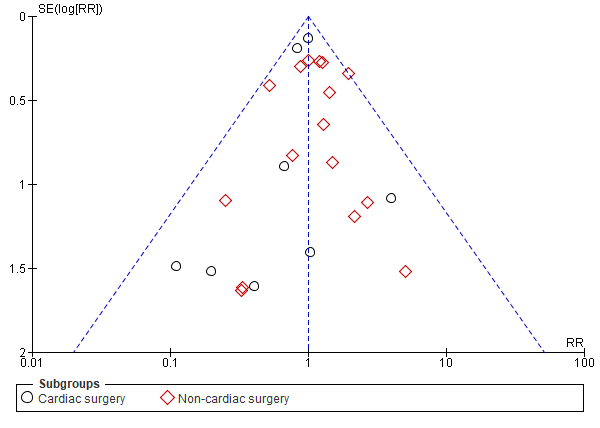


1. Atelectasis


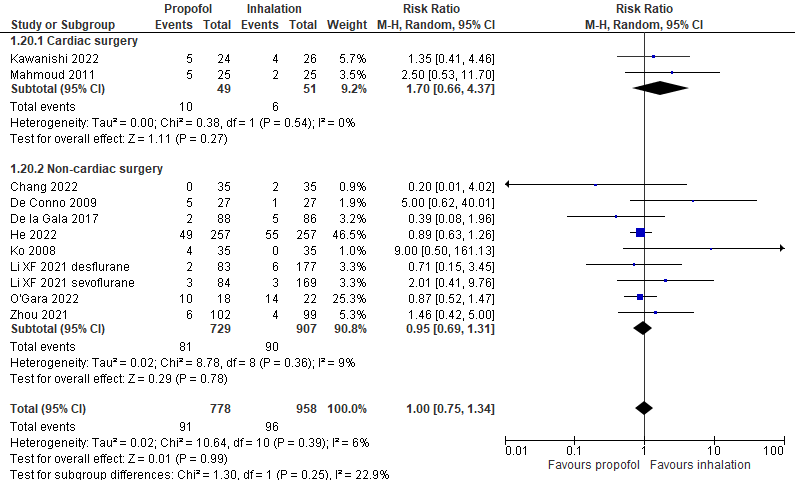


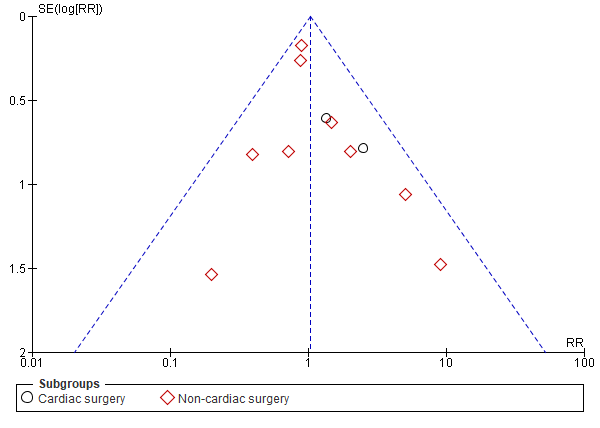


1. ARDS


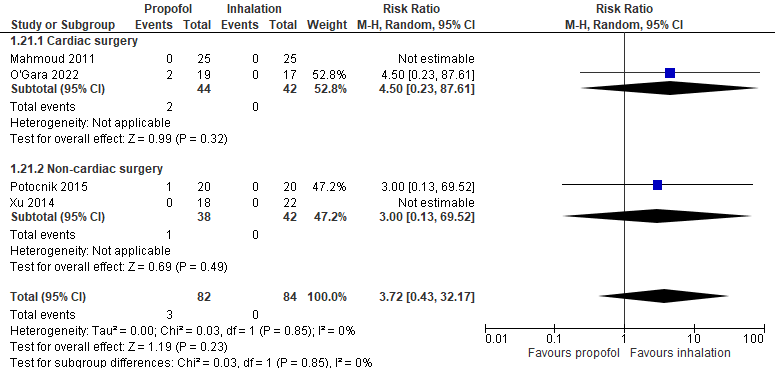


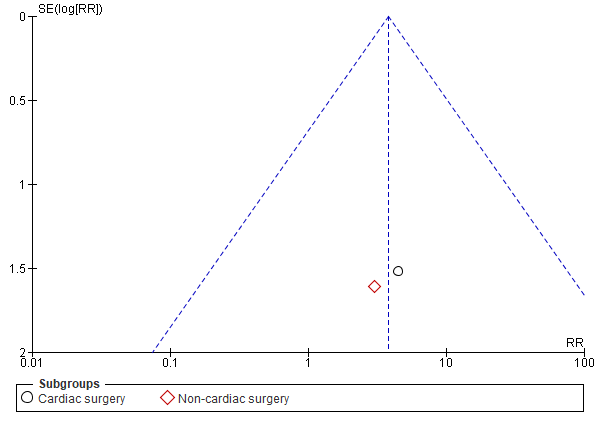


1. Aspiration


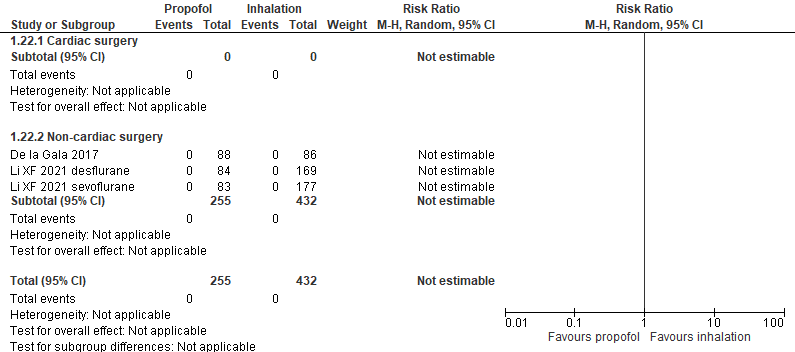


1. Postoperative cognitive dysfunction / POCD score


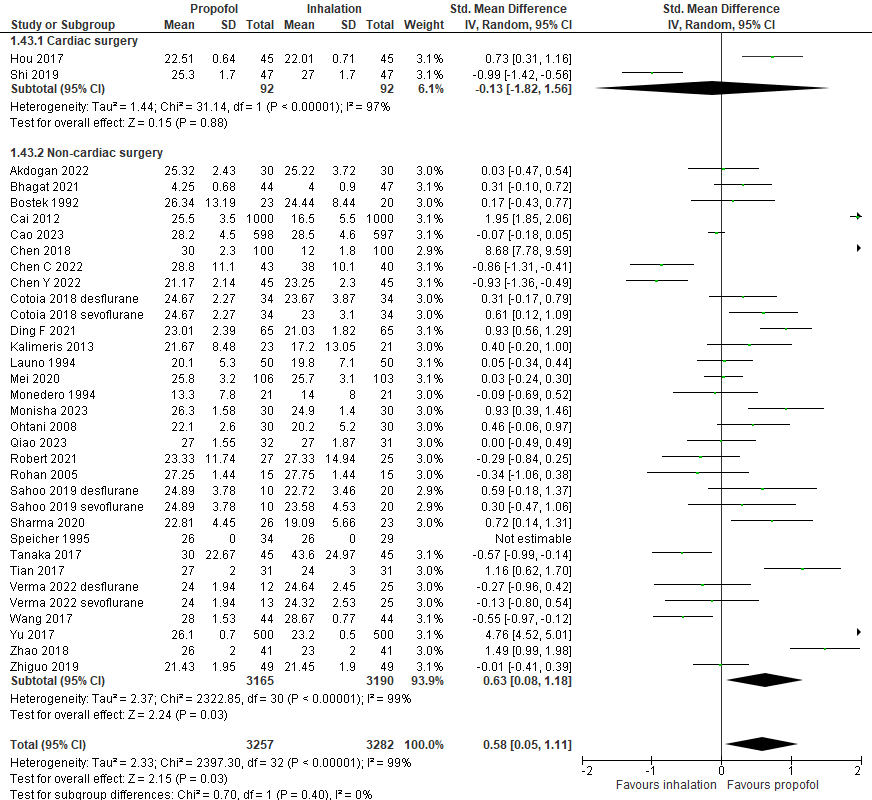


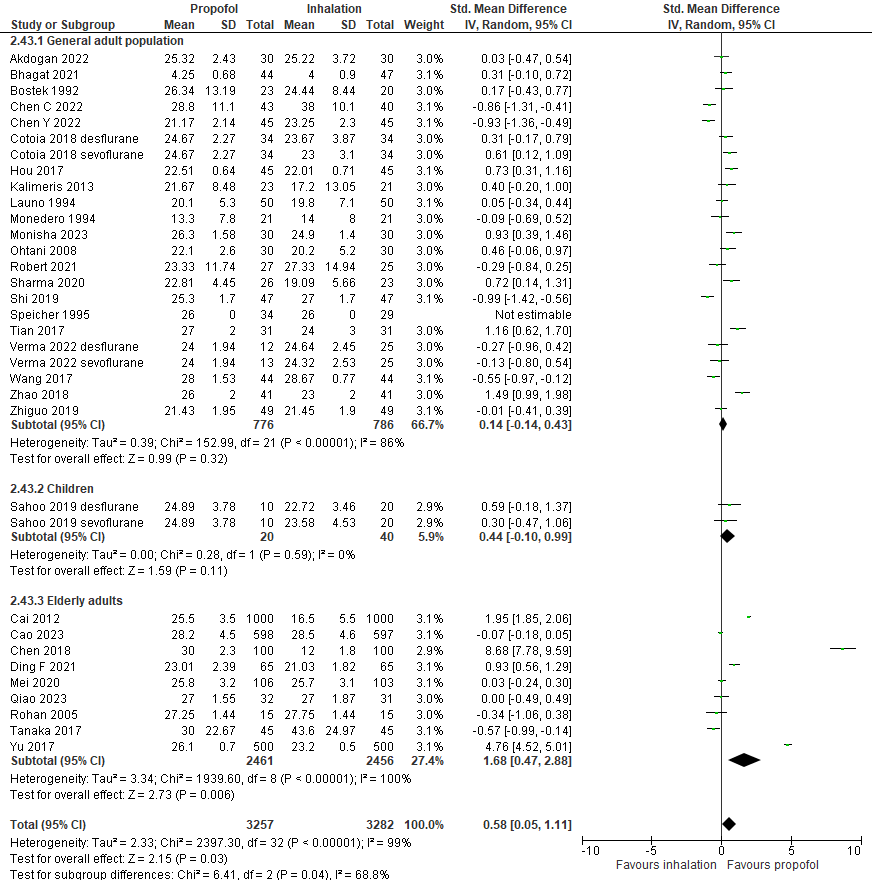


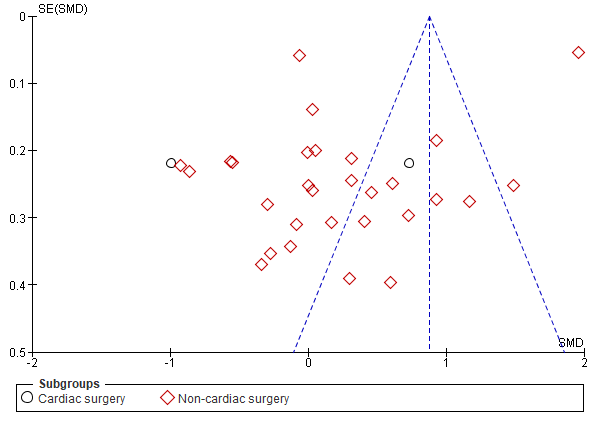


1. Postoperative cognitive dysfunction / POCD incidence


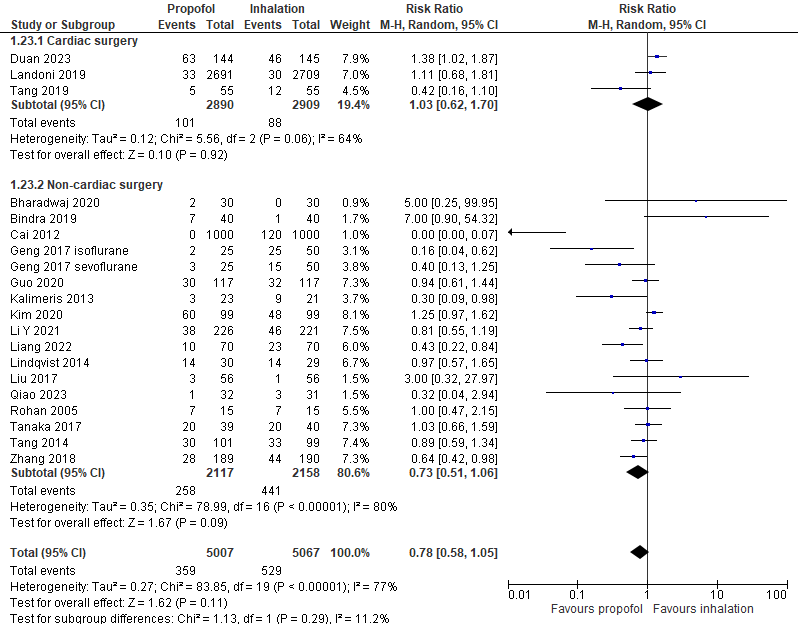


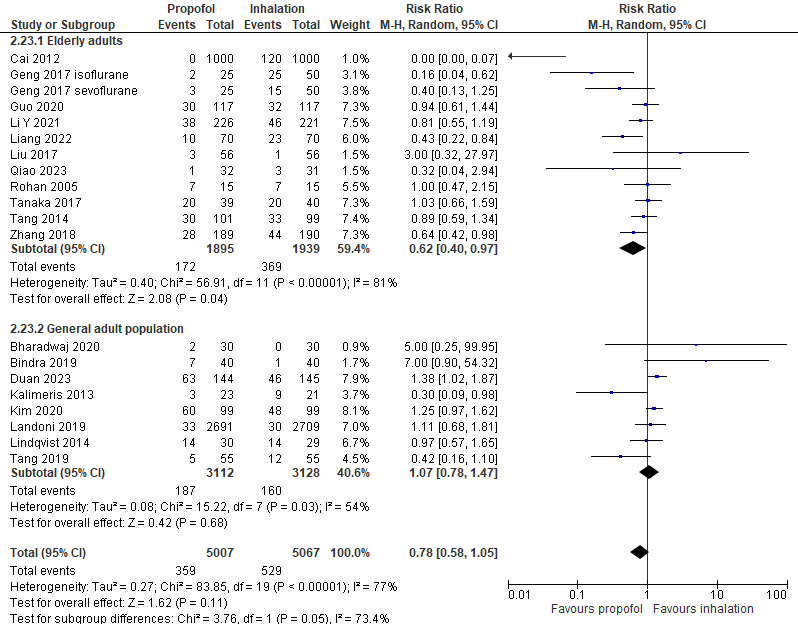


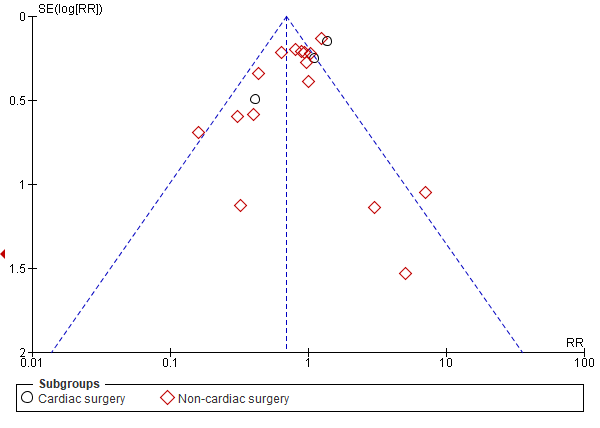


1. Delirium


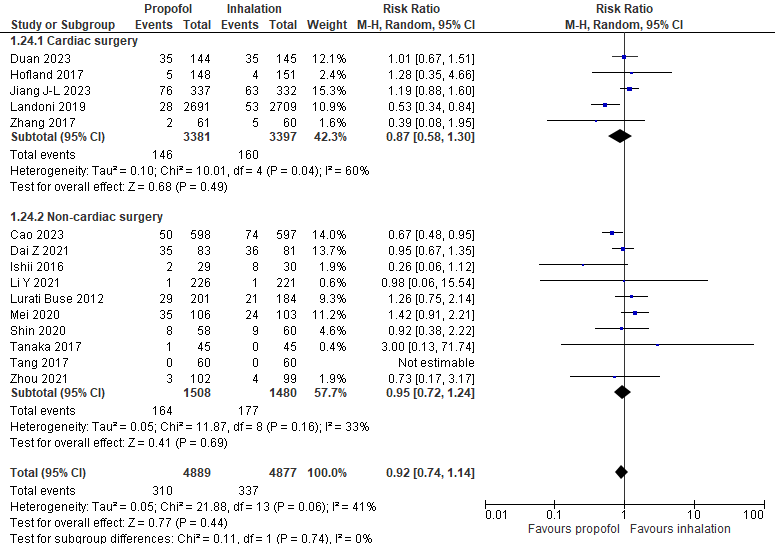


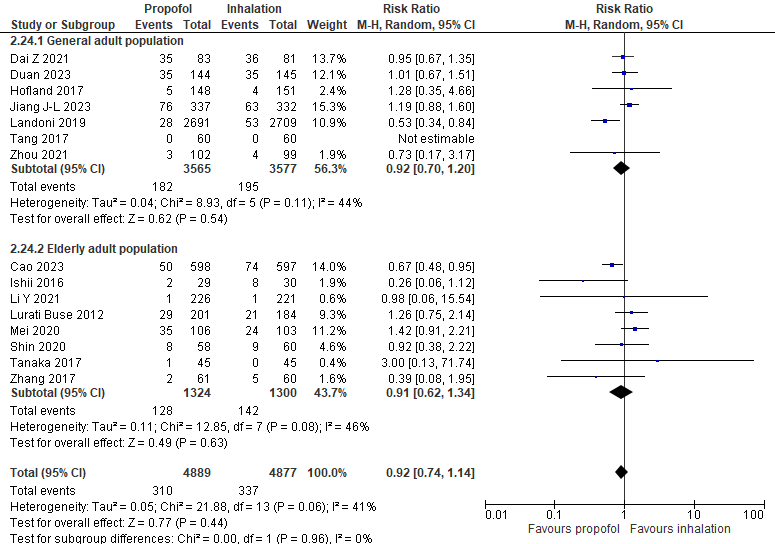


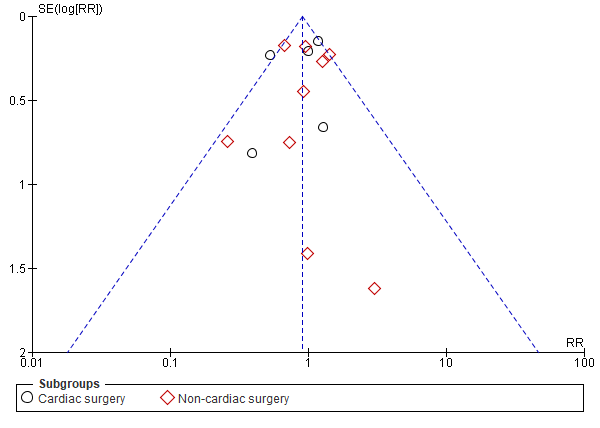


1. Cerebrovascular event / stroke


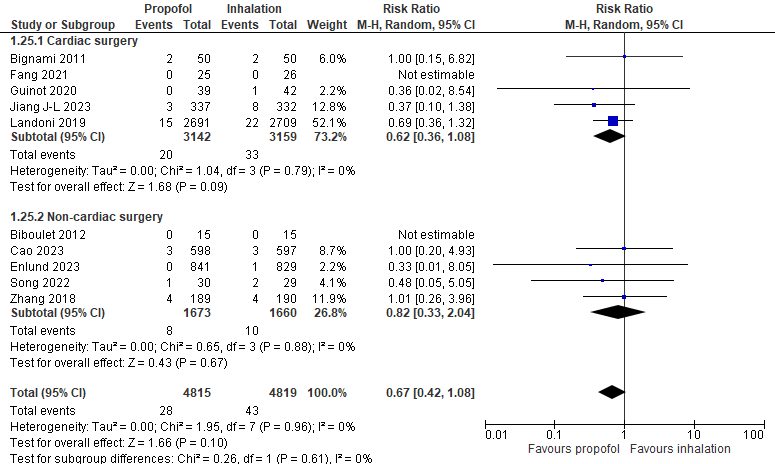


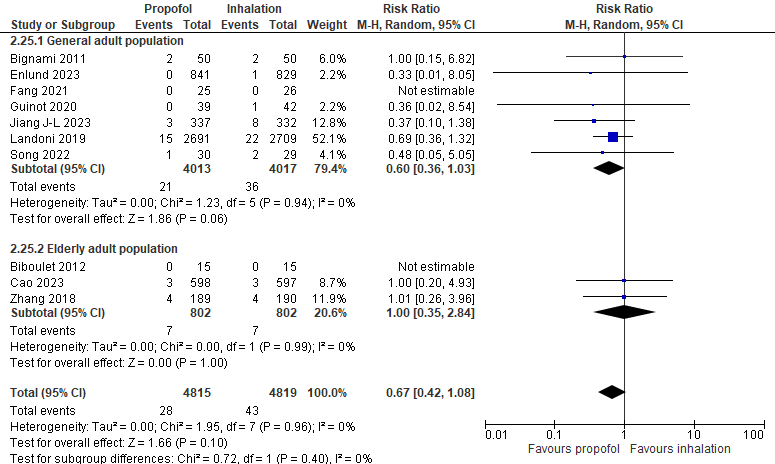


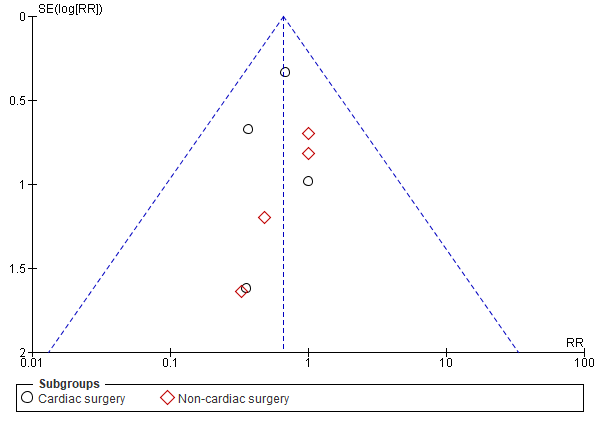


1. Acute kidney injury / AKI


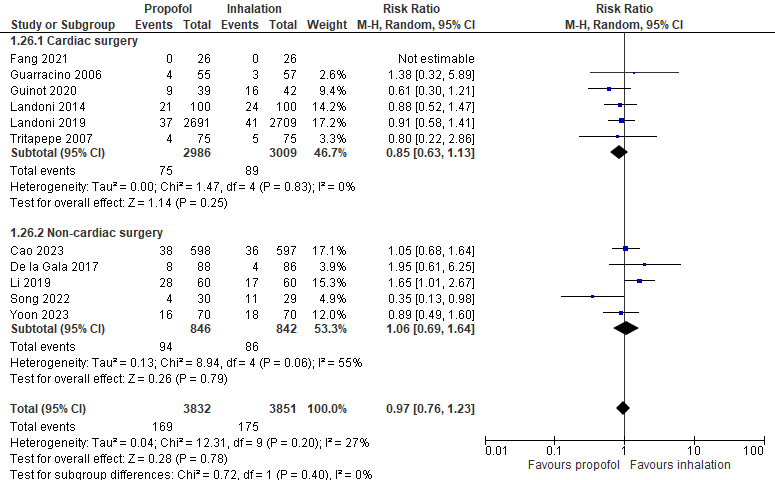


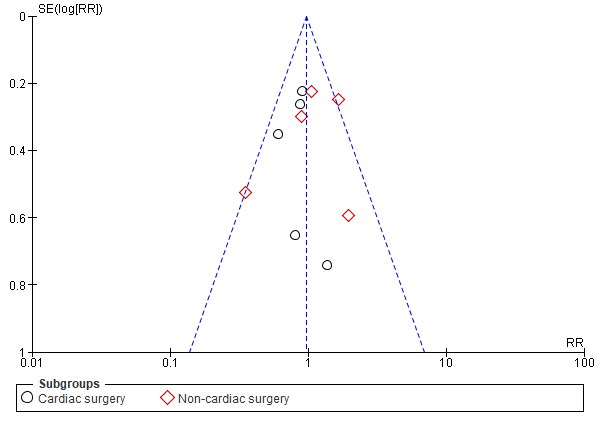


1. Renal replacement therapy / RRT


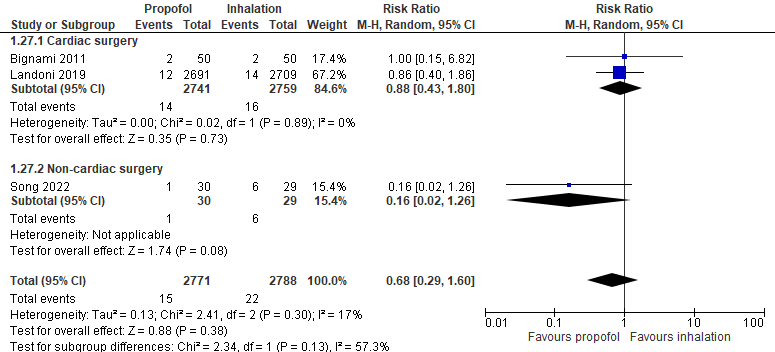


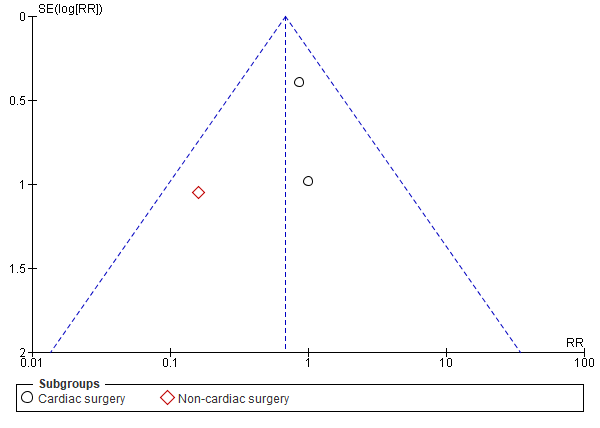


1.
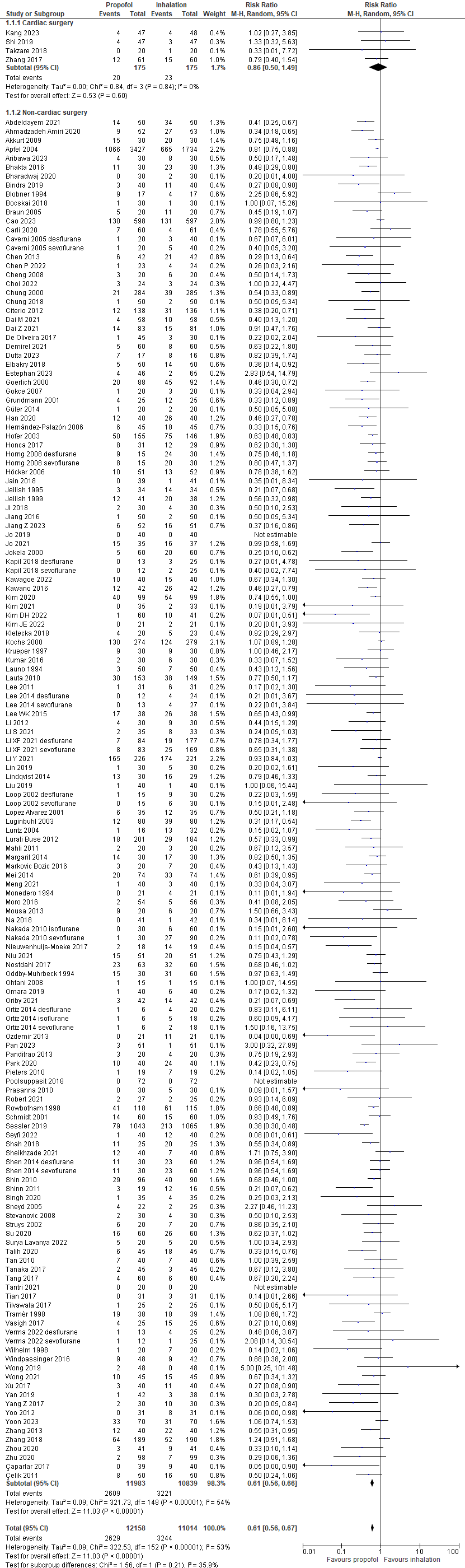
PONV


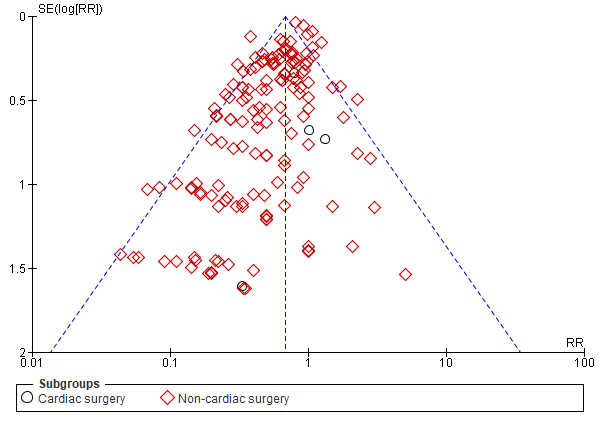


1. Agitation upon emergence


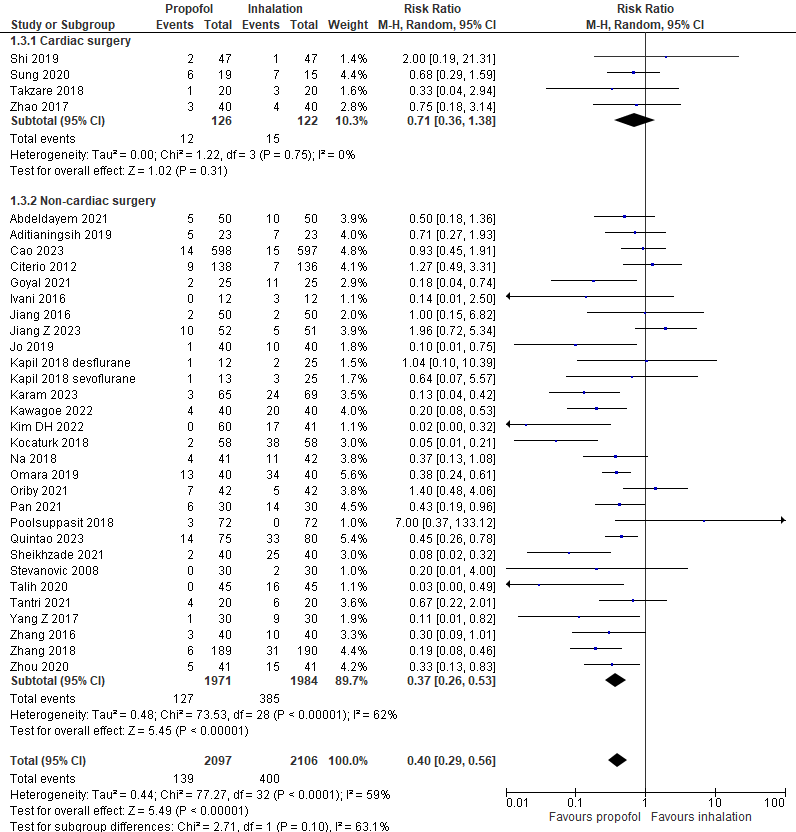


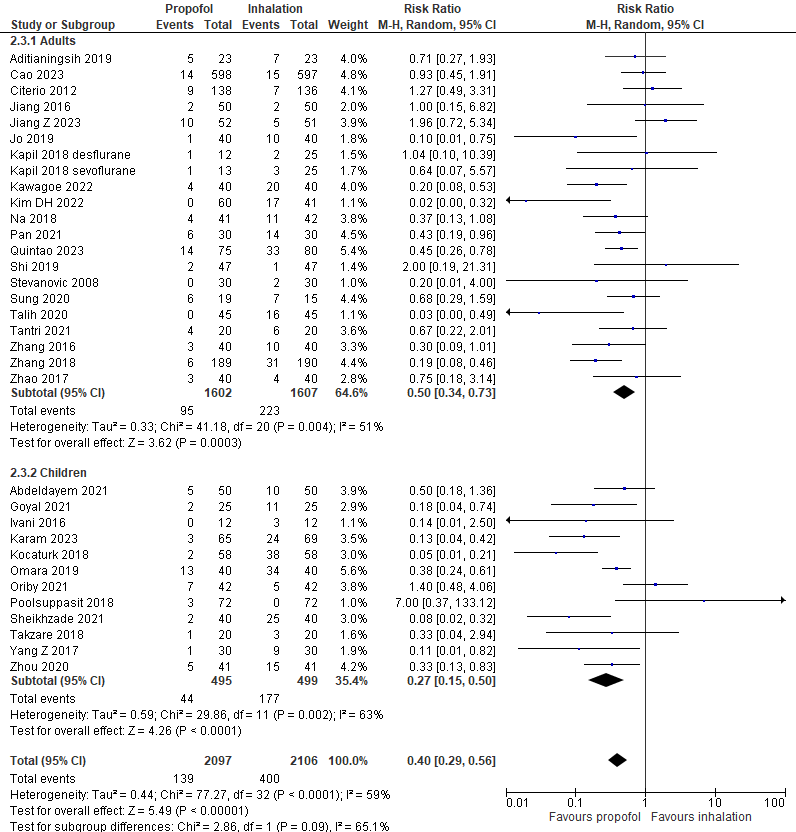


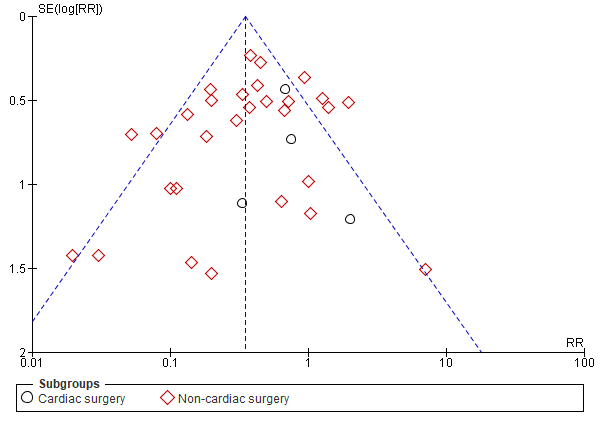


1. Postoperative shivering


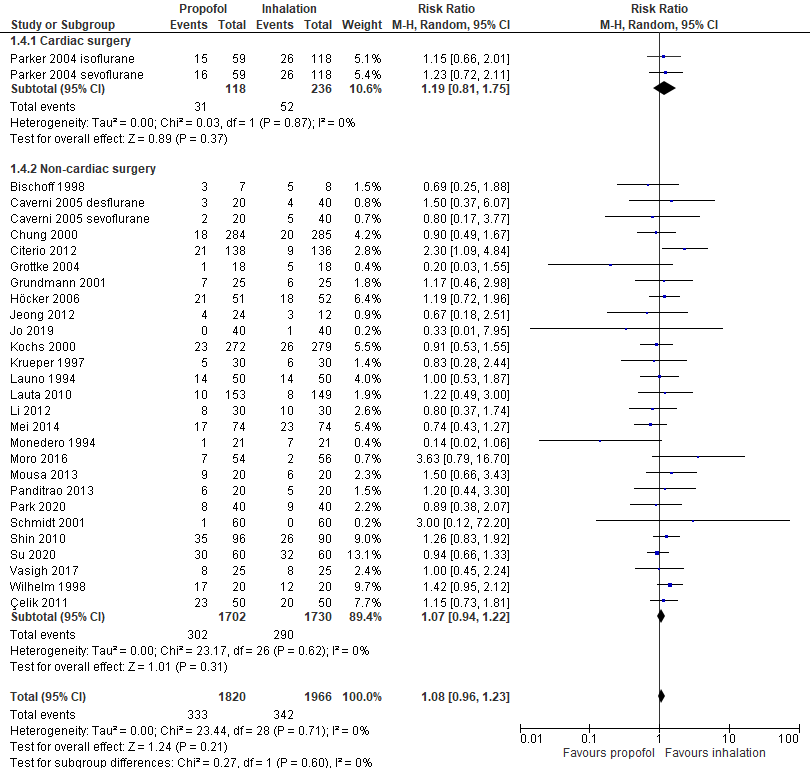


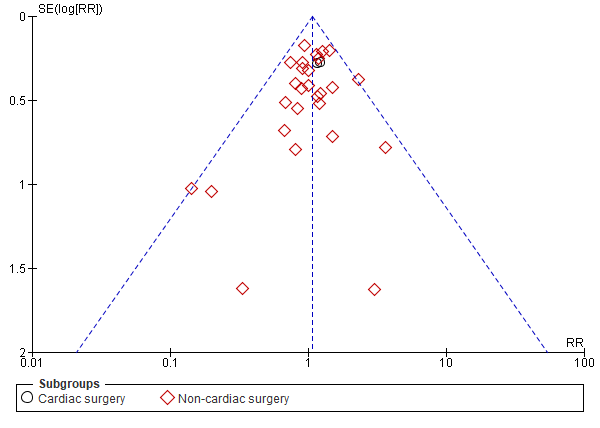


1. Awareness / accidental awakening


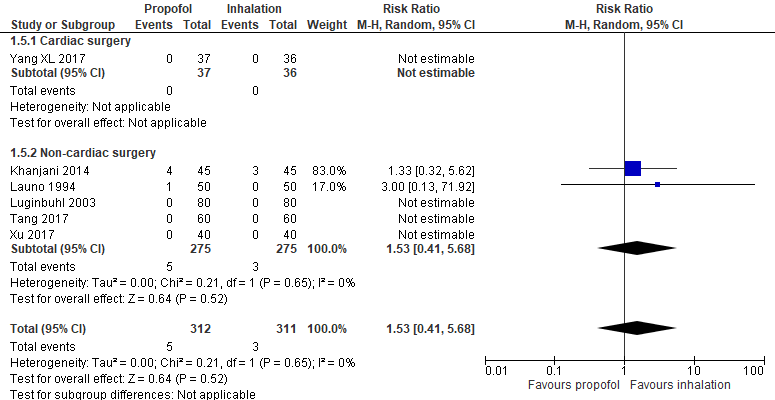


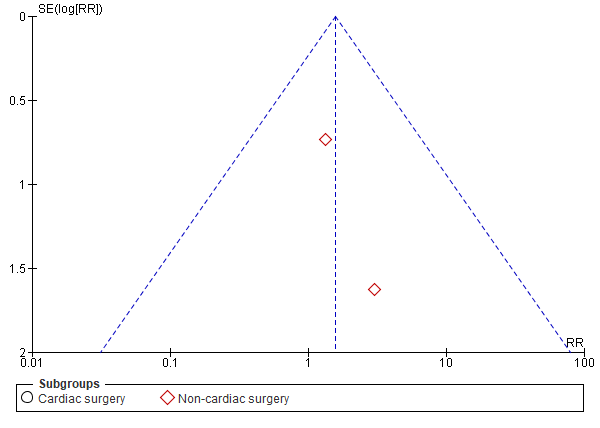


1. Qor-40


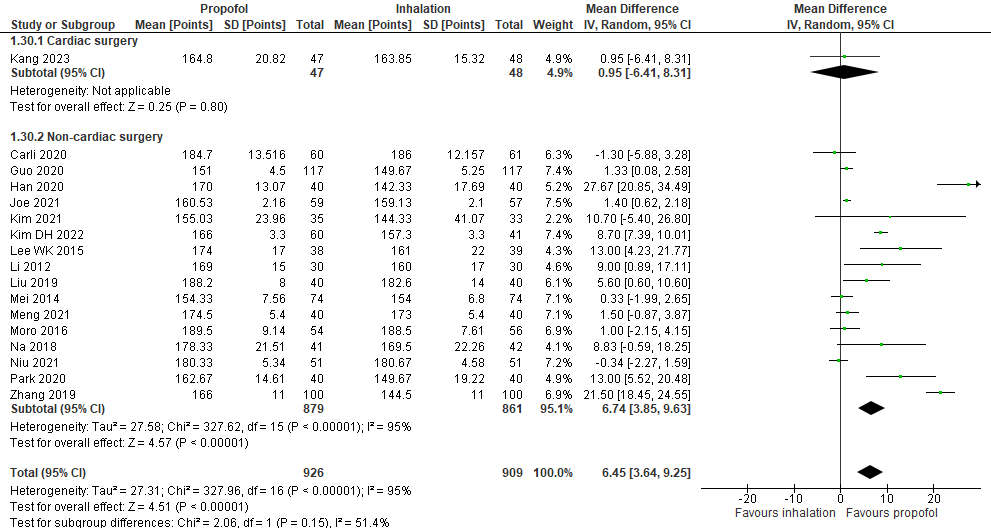


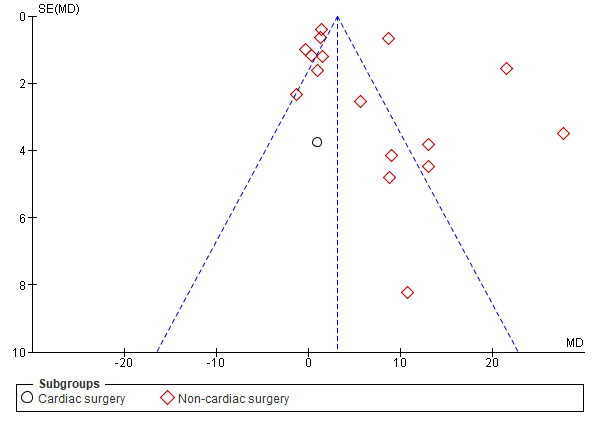


1. QoR-15


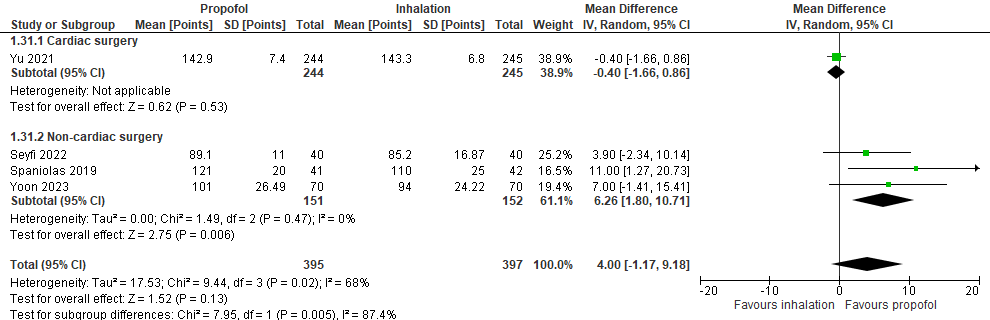


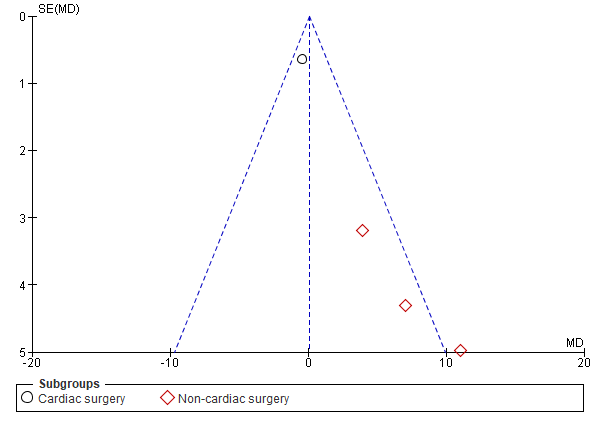


1. First pain score <12 hours postoperatively


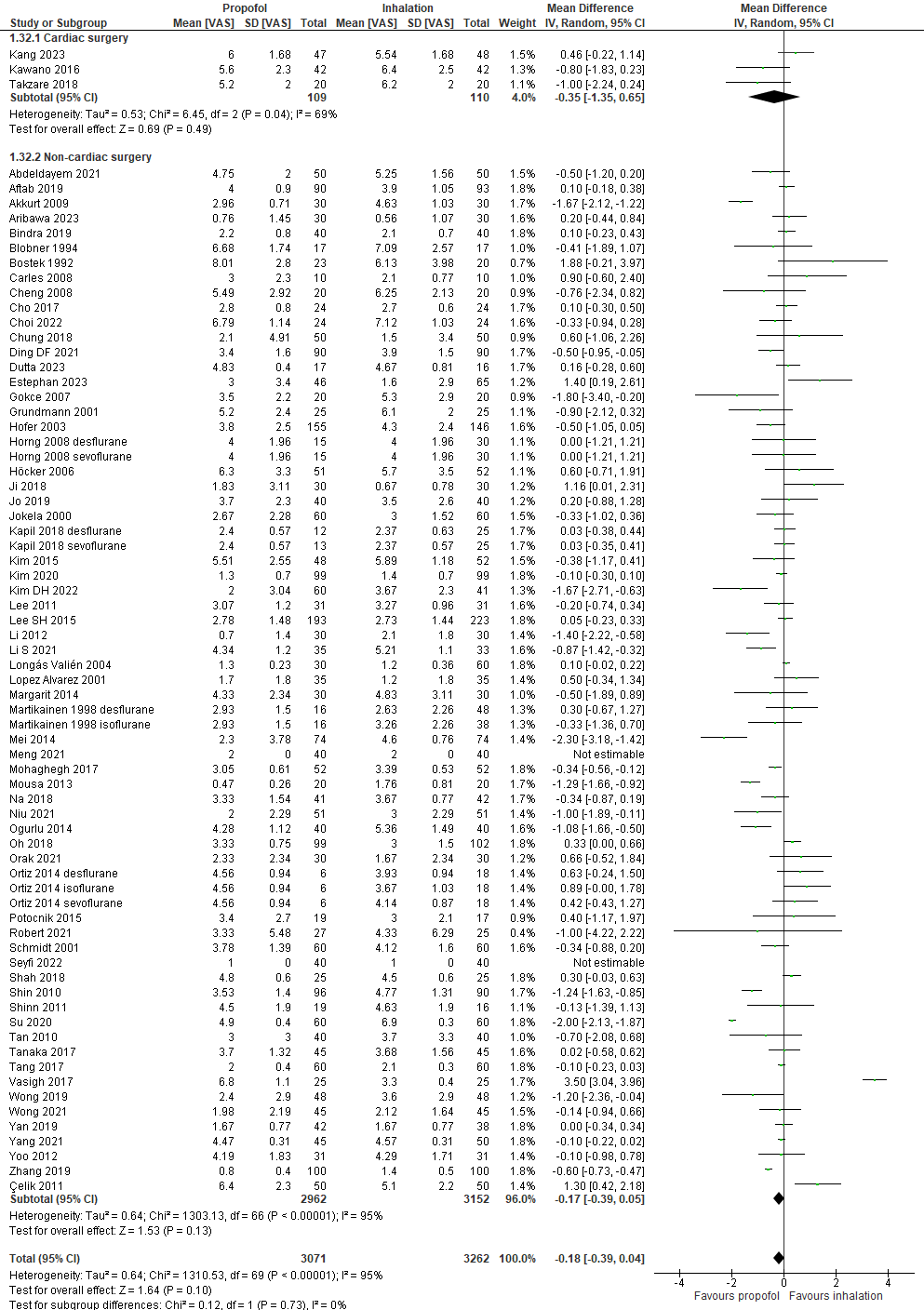


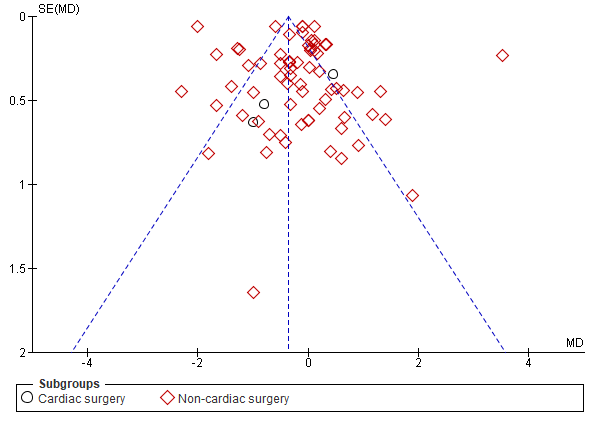


1. Pain score 12-24 hours postoperatively


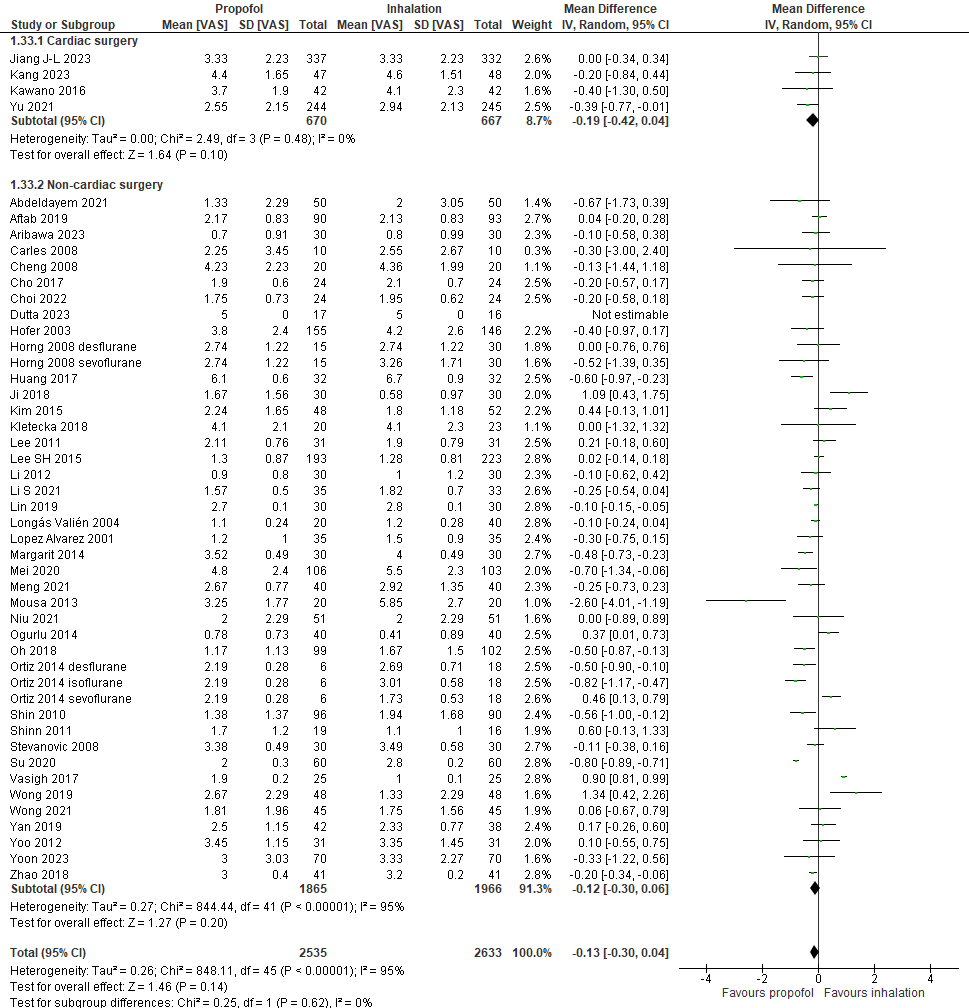


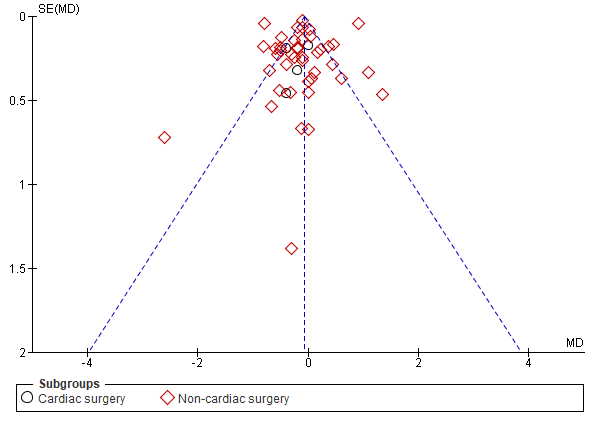


1. Pain score >24 hours postoperatively


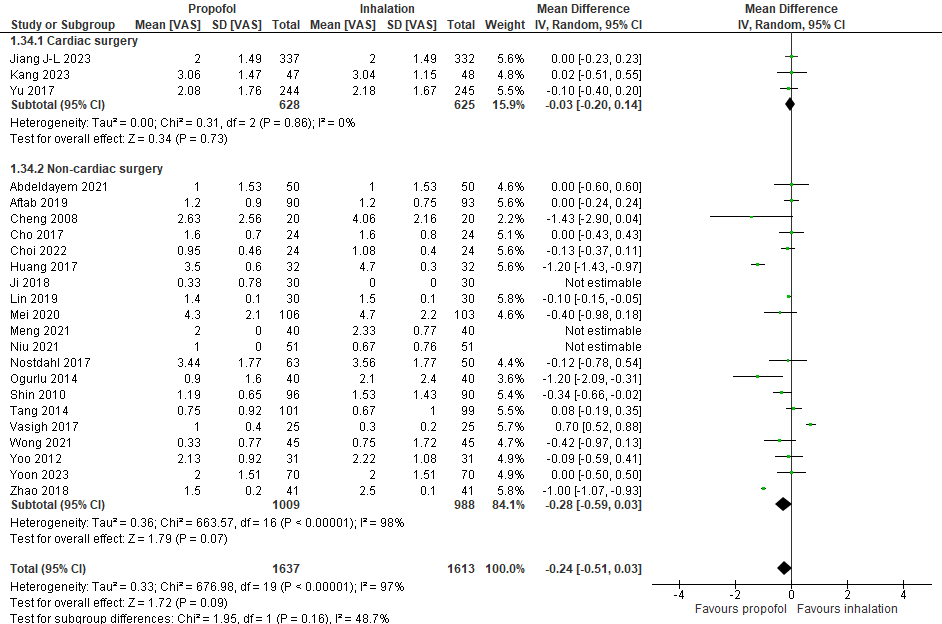


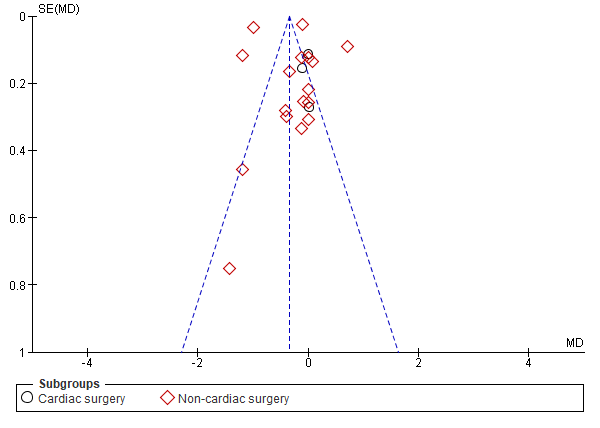


1. Clavien-Dindo grades ≥3


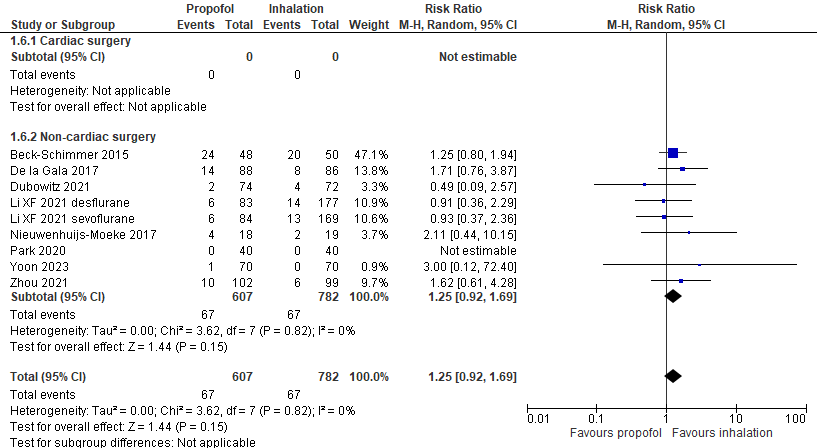


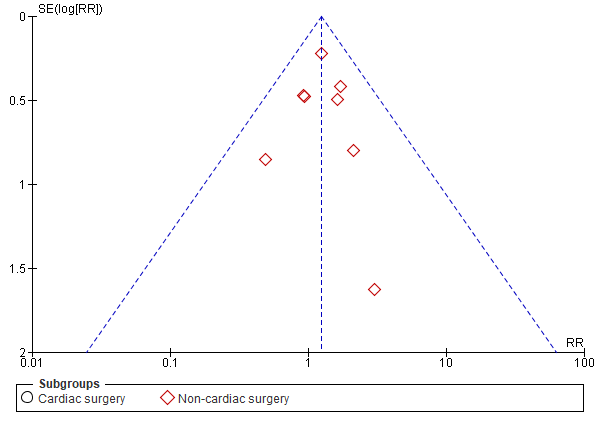


1. Major bleeding


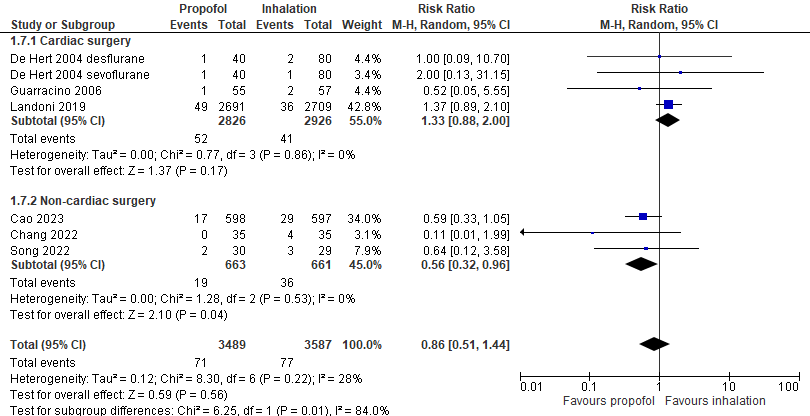


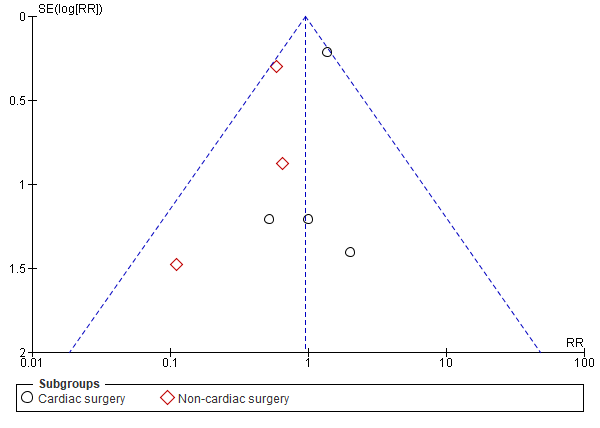


1. Surgical site infection


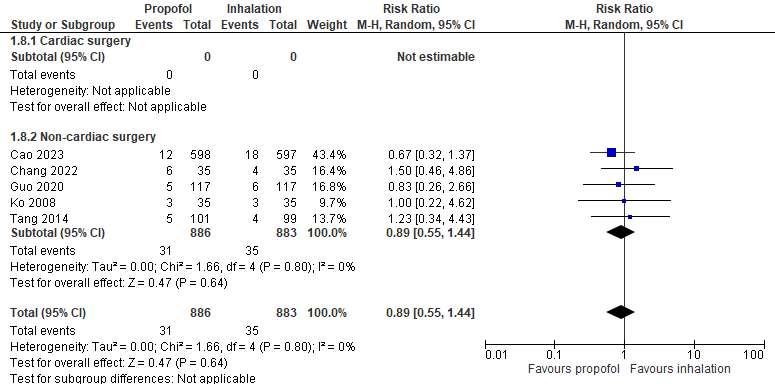


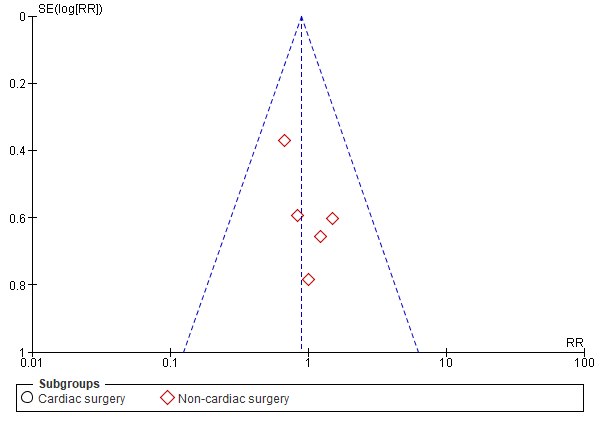


1. Cancer recurrence


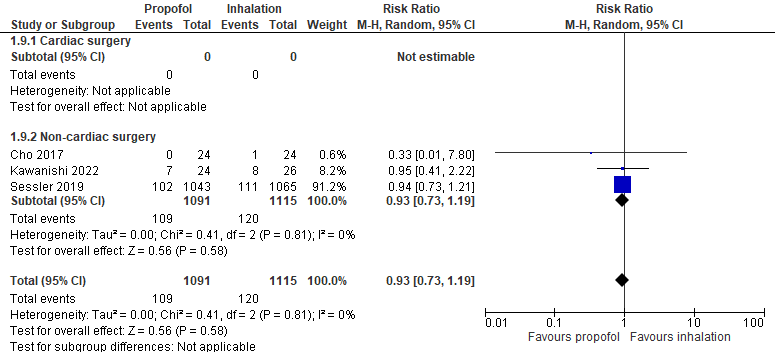


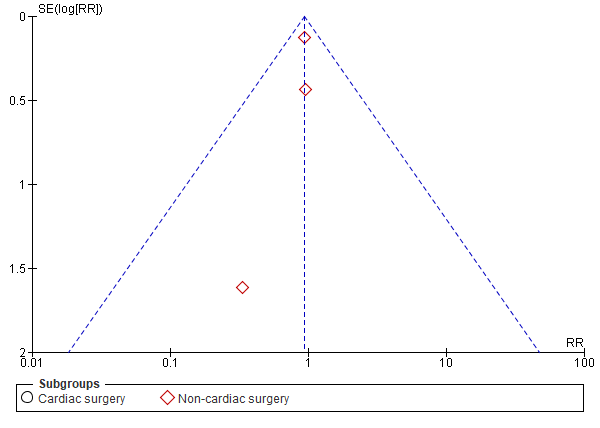


1. Intraoperative opioid consumption


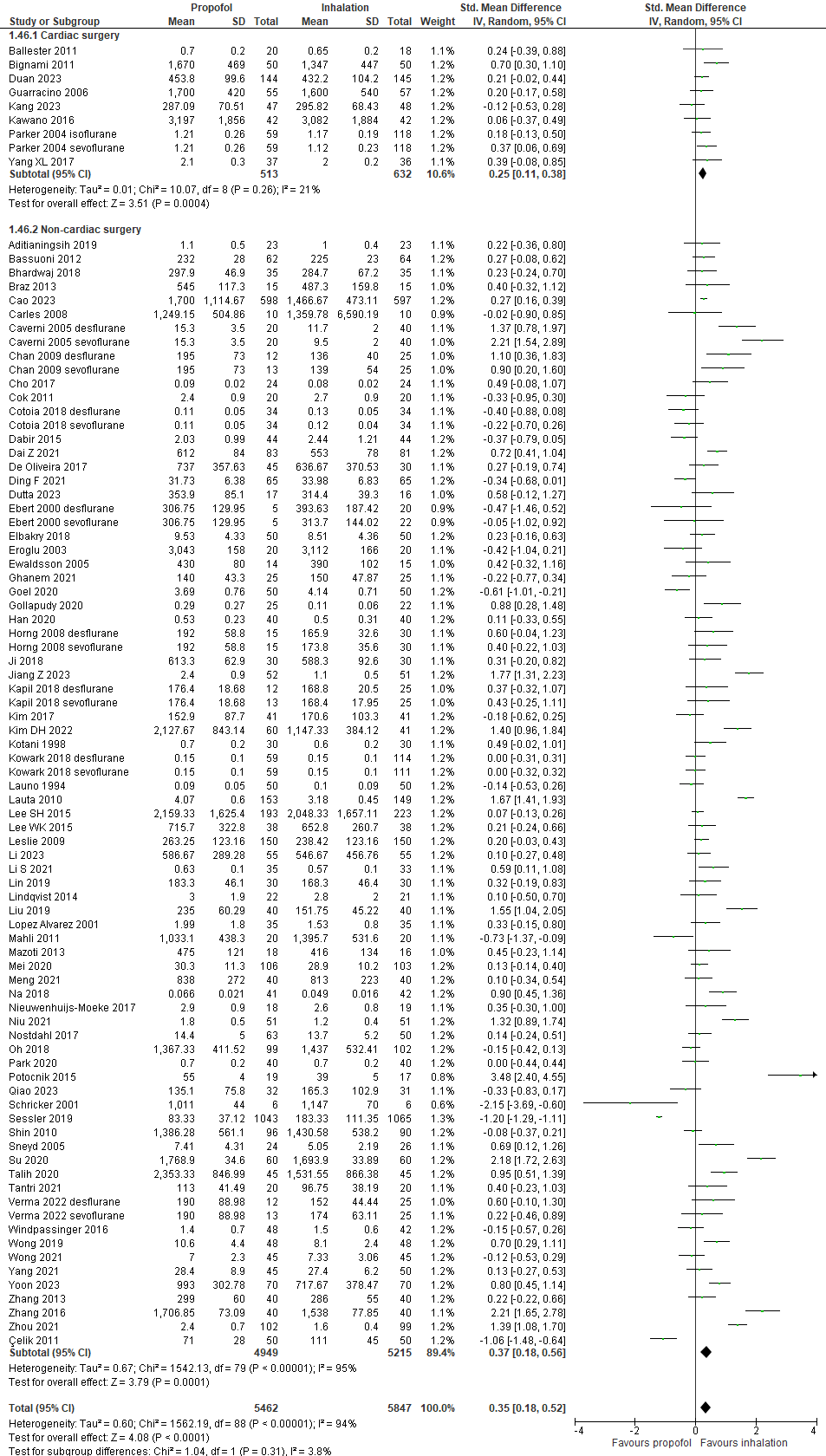


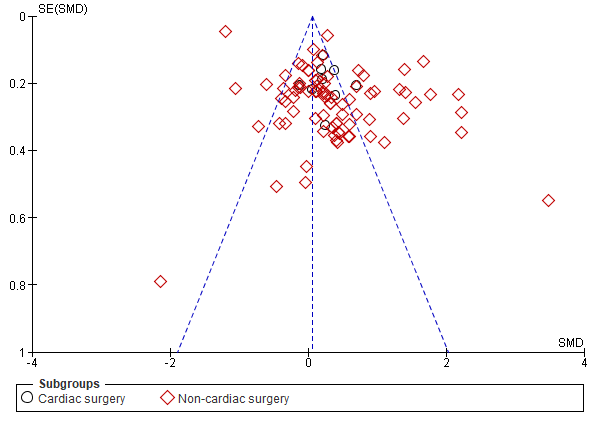


1. Postoperative opioid consumption


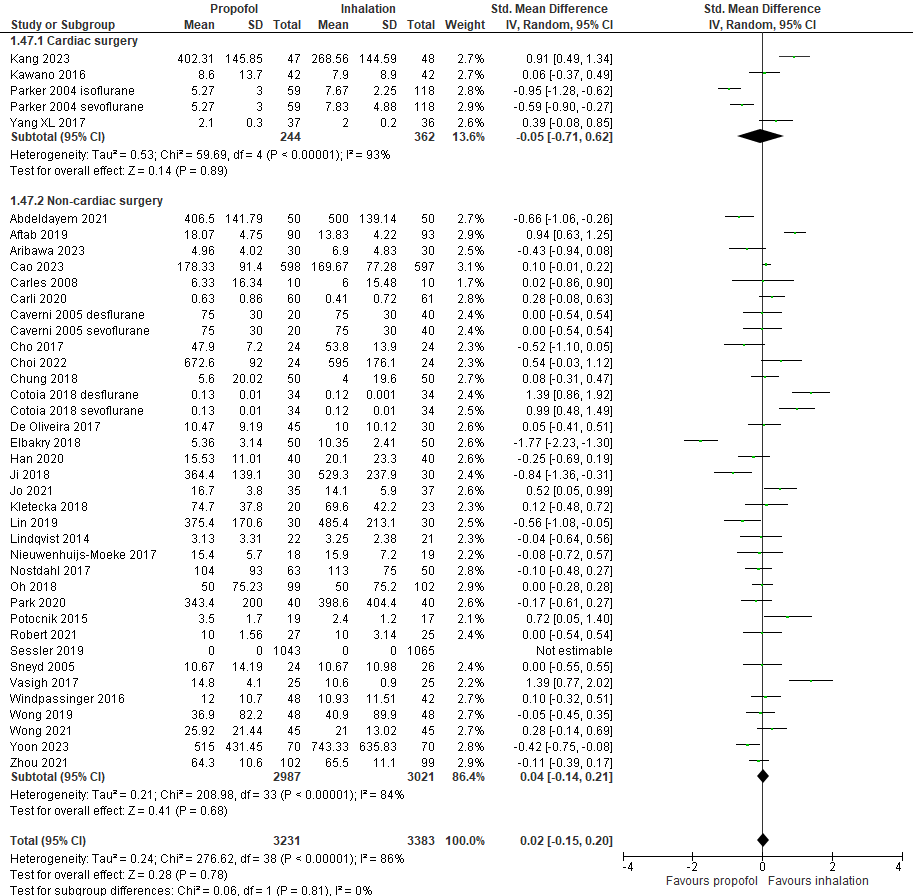


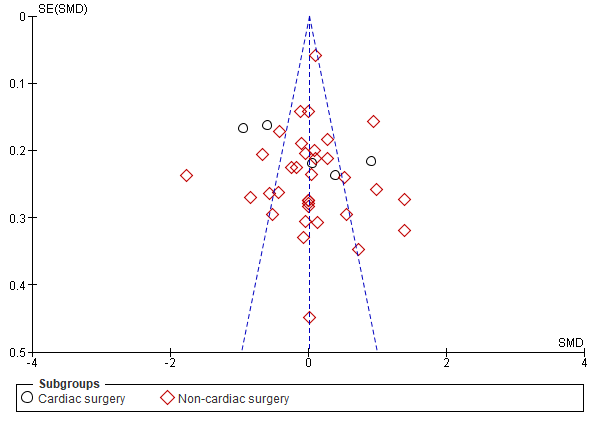


1.
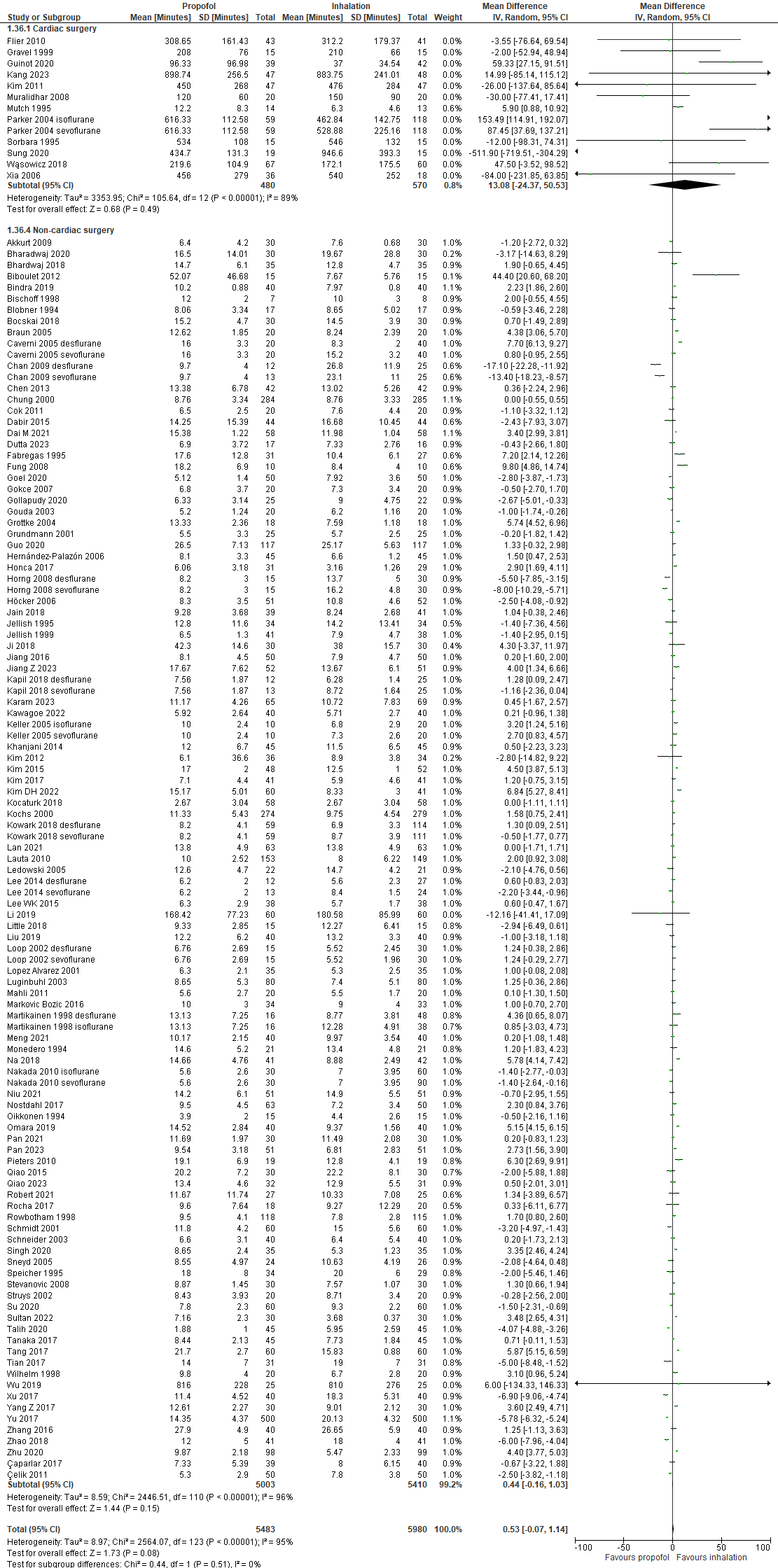
Time to extubation


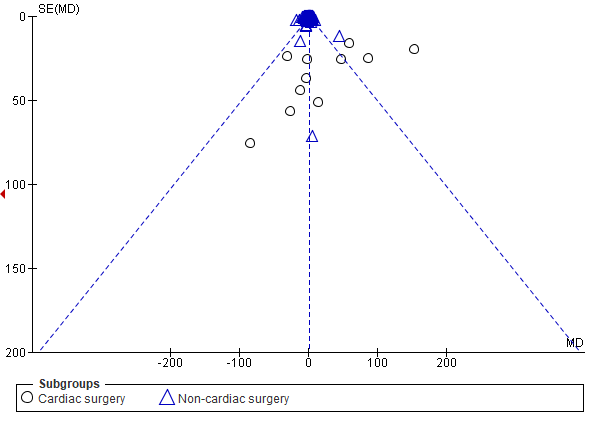


1. Time to awakening


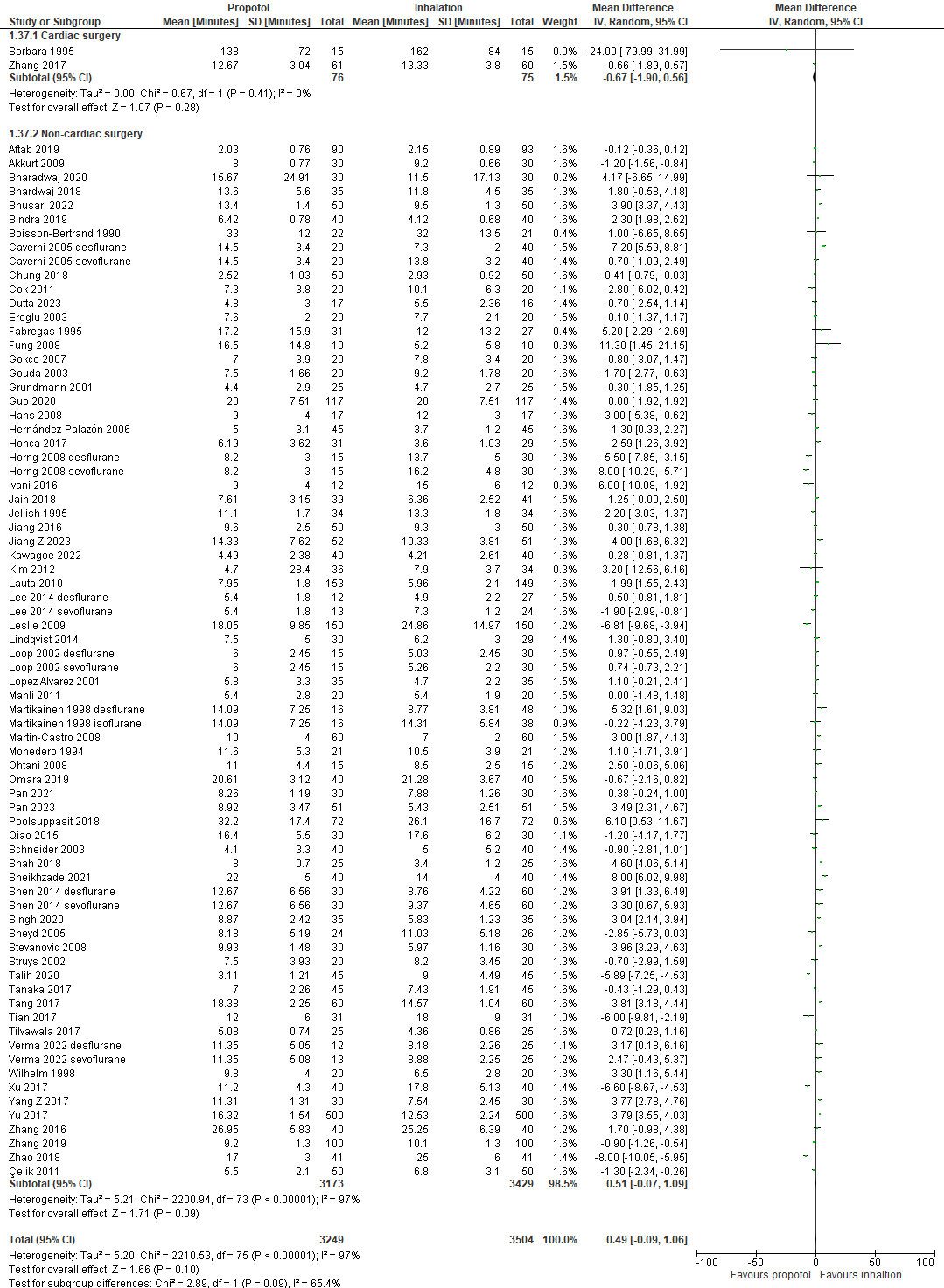


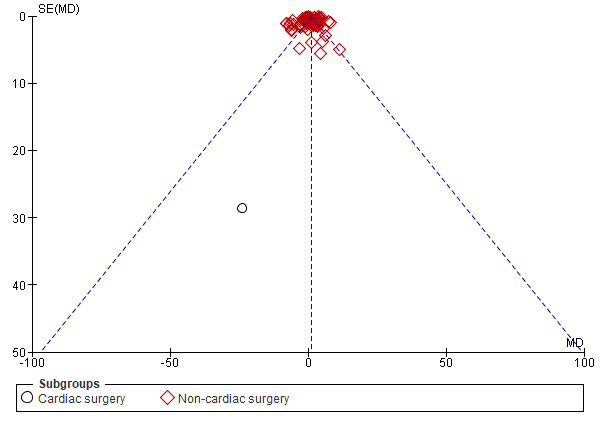


1. Time to follow simple instruction


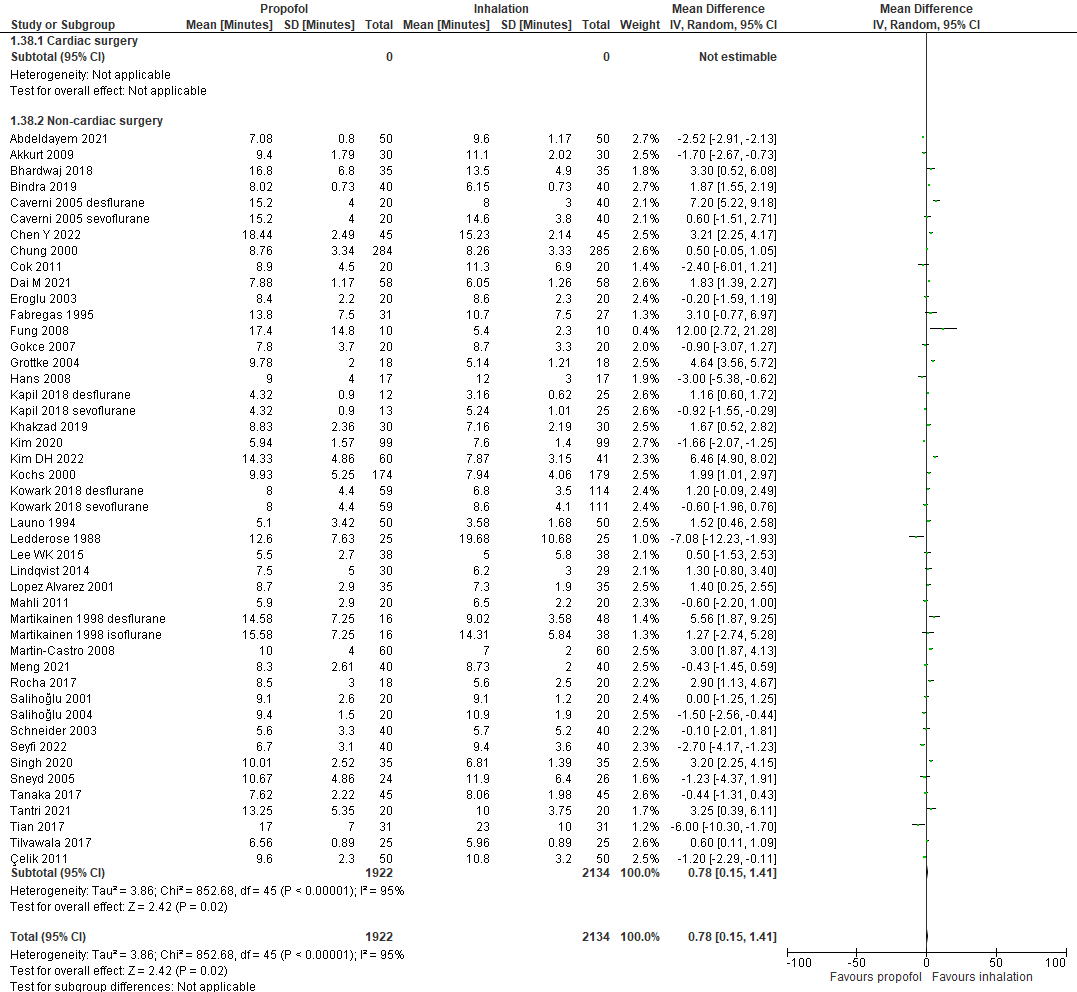


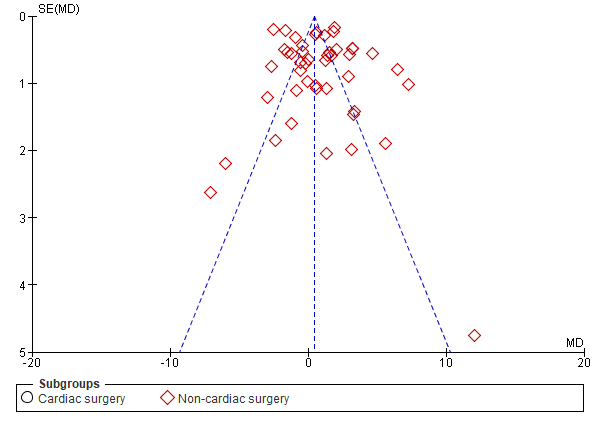


1. Time to respiratory recovery


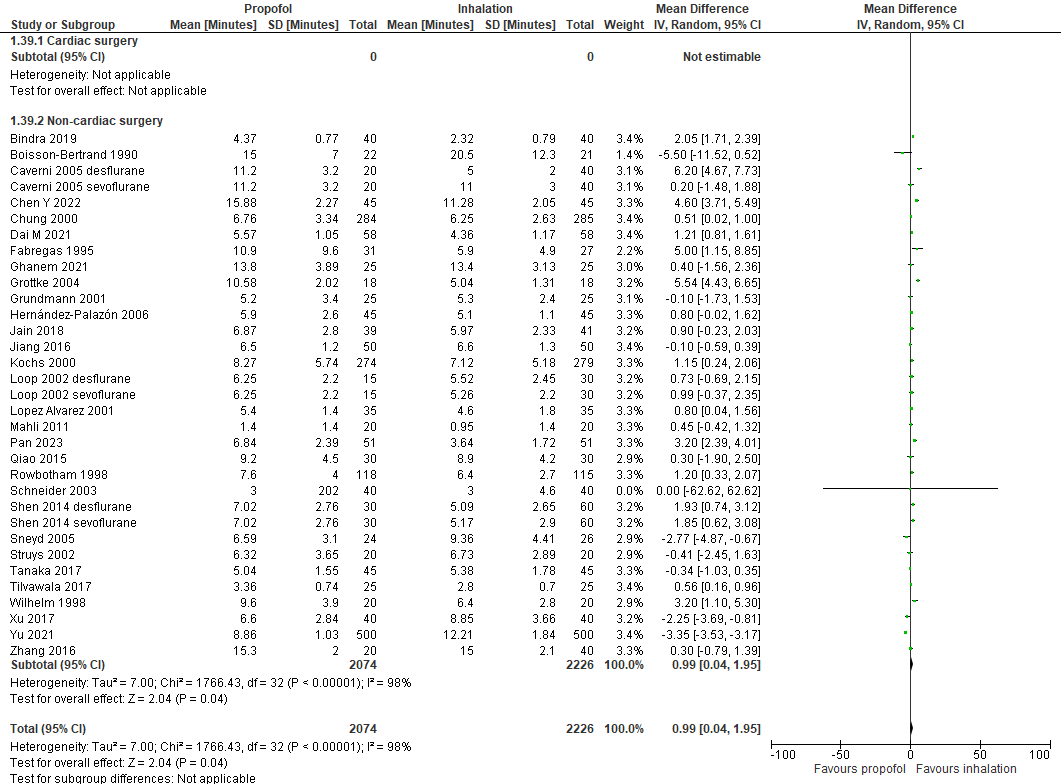


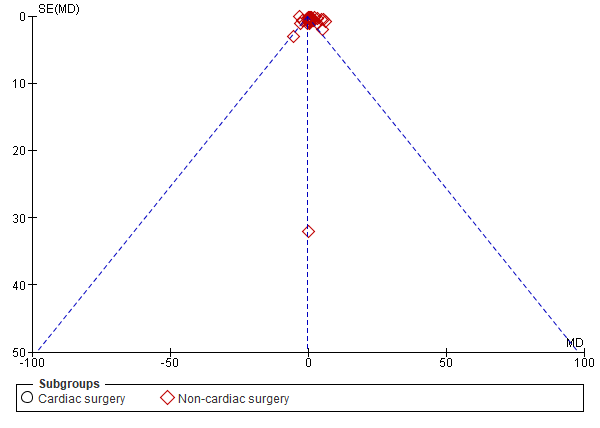


1. Time to reach a recovery score


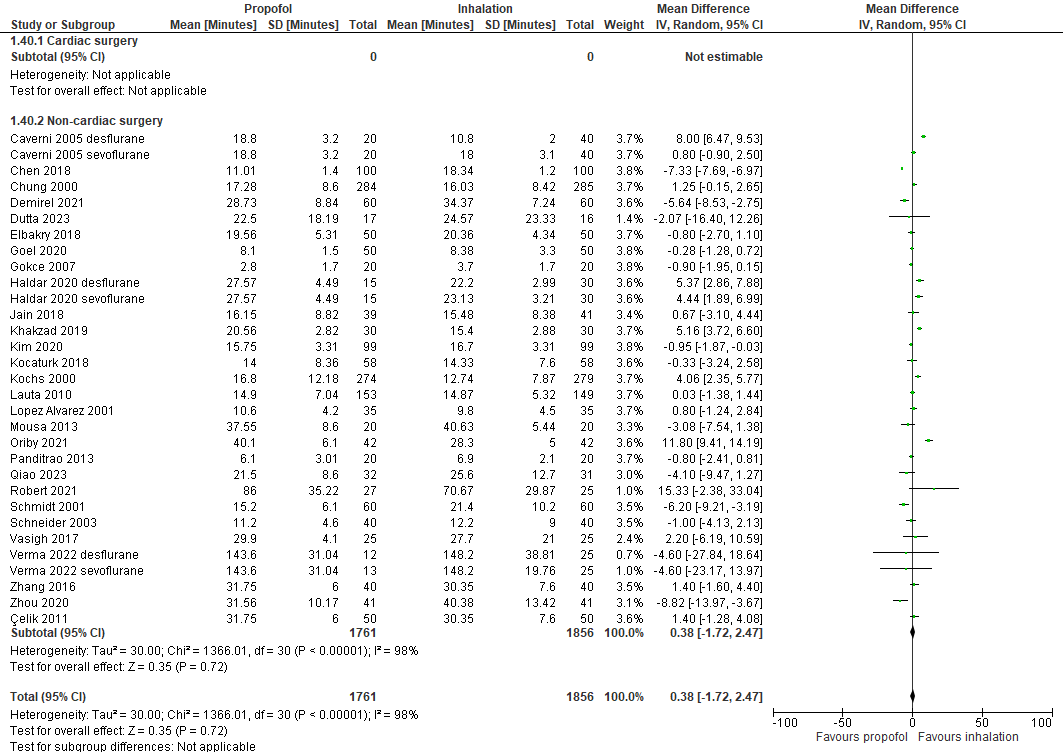


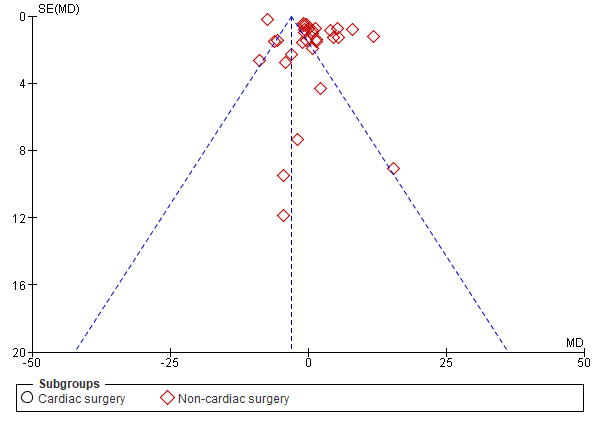


1. Time to orientation


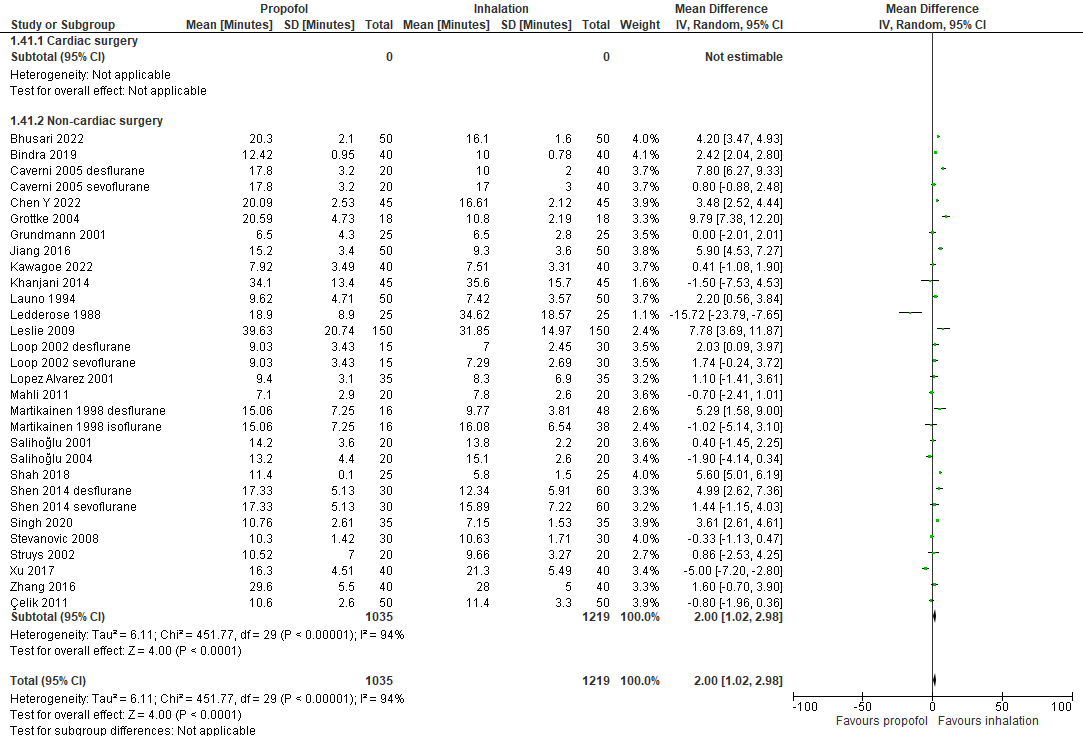


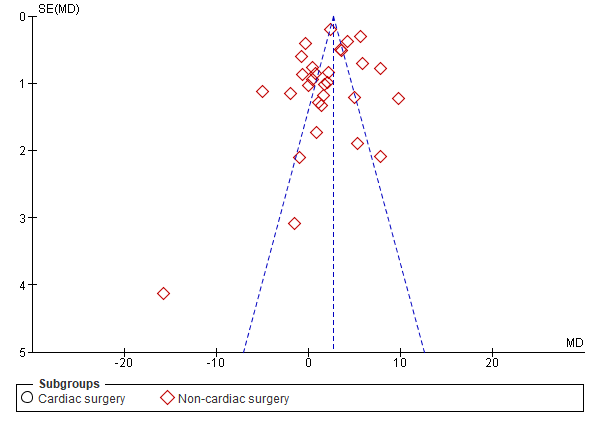


1. PACU time


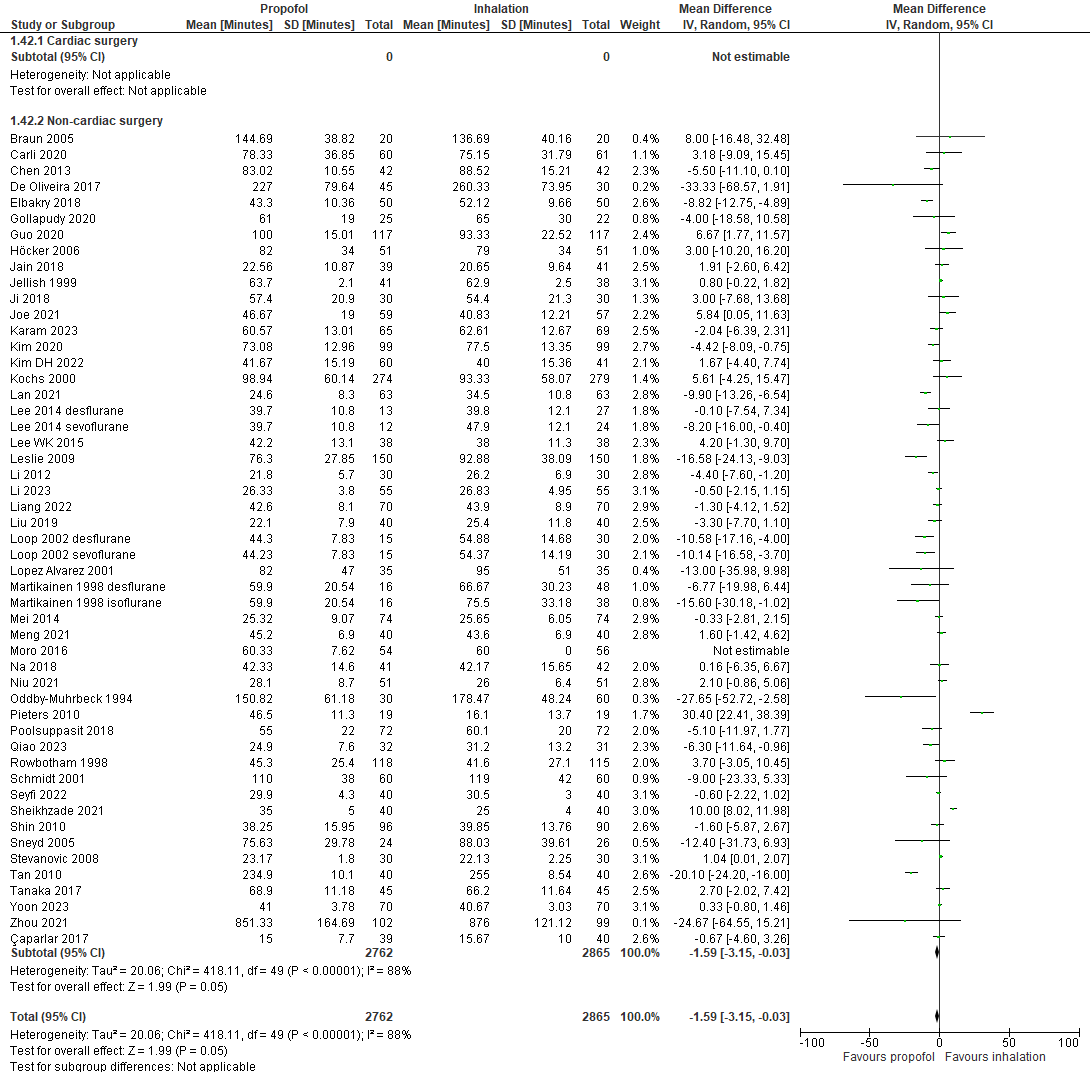


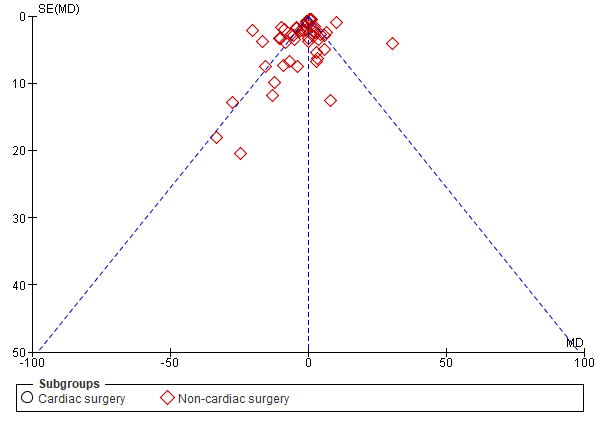


1.
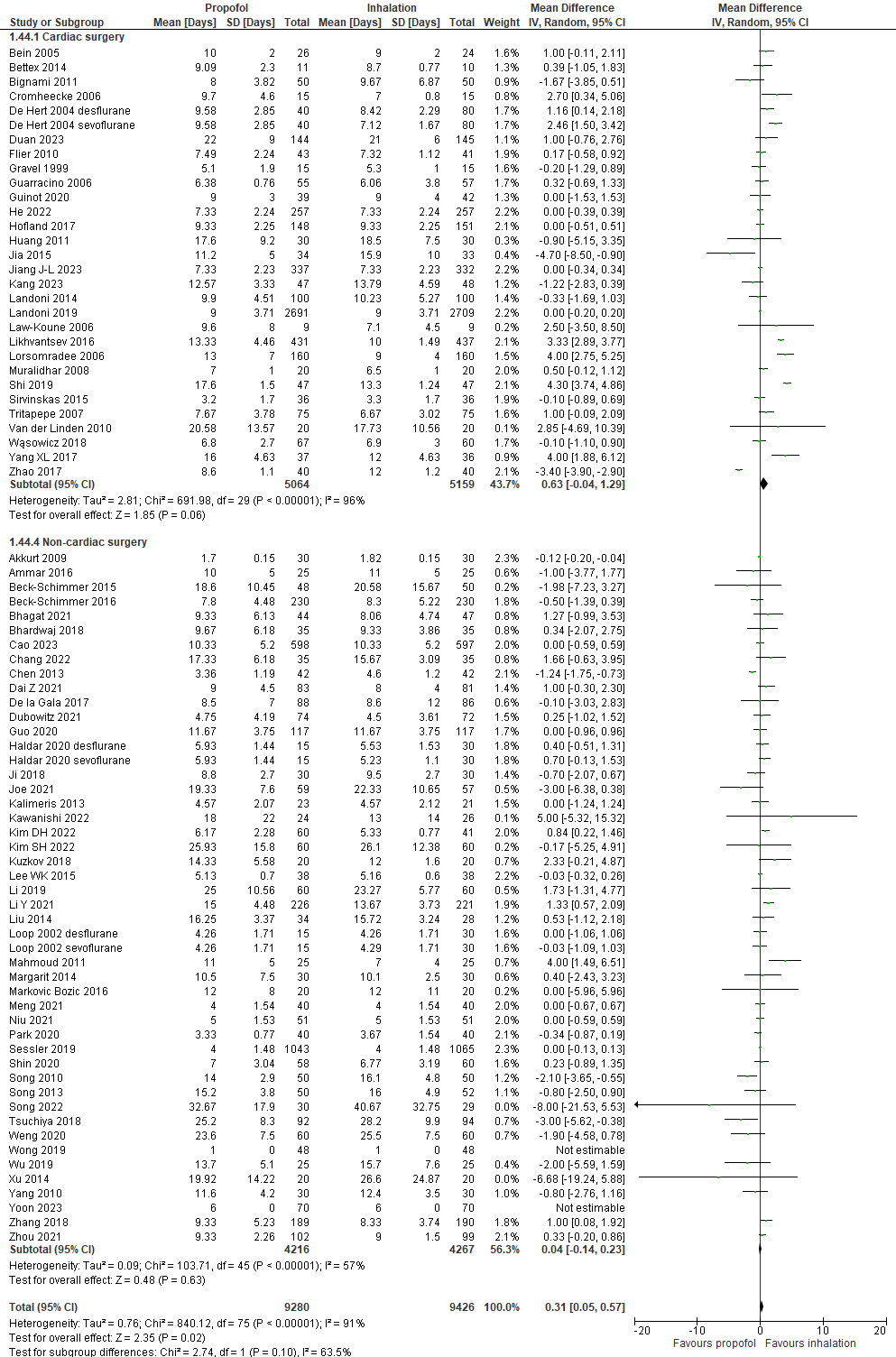
Length of hospital stay / LoS


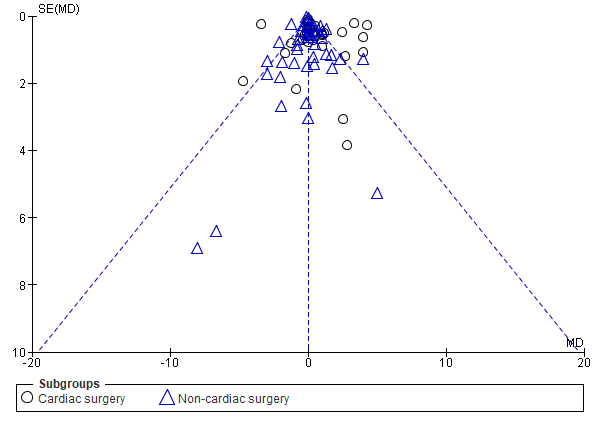


1. Unplanned hospital (re)admissions


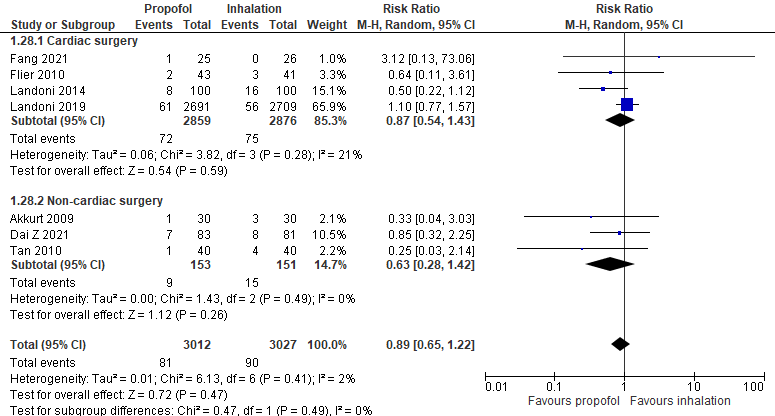


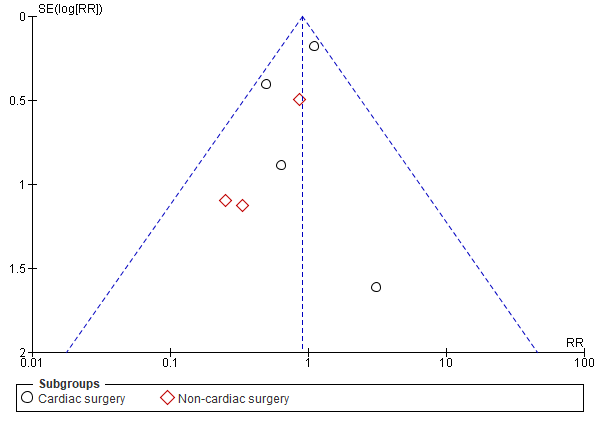


1. Unplanned ICU admissions


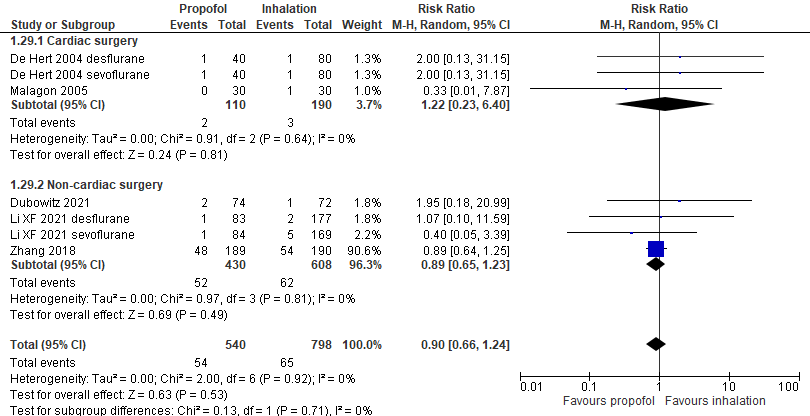


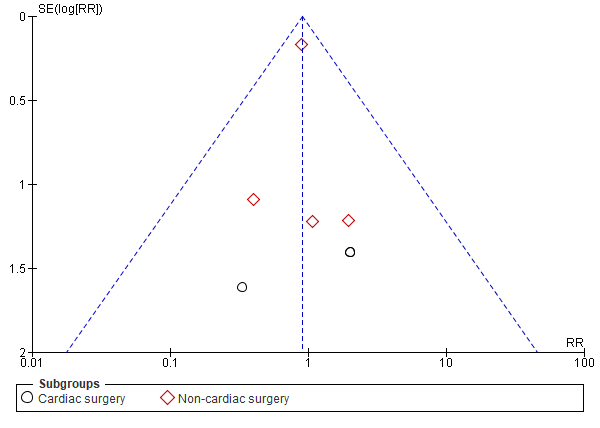


1. Cost analysis


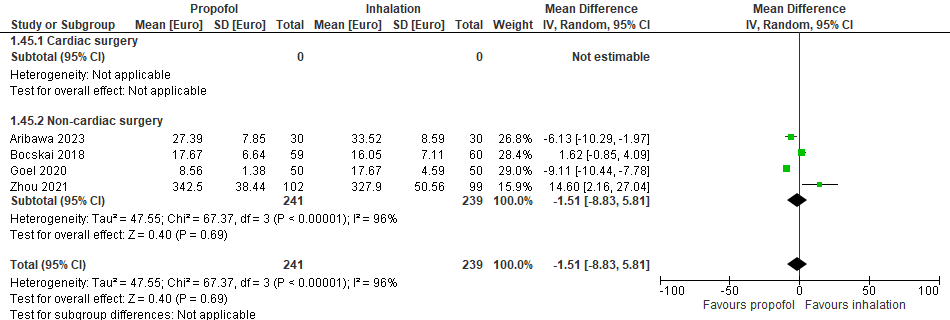


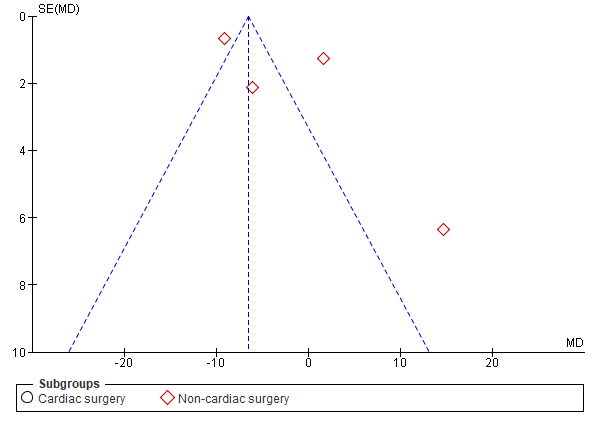

Supplement: Appendix 1–10 [file mmc1.docx]
